# Plant bioactive compounds: extraction, biological activities, immunological, nutritional aspects, food application, and human health benefits—A comprehensive review

**DOI:** 10.3389/fnut.2025.1659743

**Published:** 2025-12-19

**Authors:** Mohamed T. El-Saadony, Ahmed M. Saad, Dina Mostafa Mohammed, Samar Sami Alkafaas, Taia A. Abd El-Mageed, Mohamed A. Fahmy, Ahmed Ezzat Ahmed, Uthman Balgith Algopishi, Abdelghafar Mohamed Abu-Elsaoud, Walid F. A. Mosa, Synan F. AbuQamar, Khaled A. El-Tarabily

**Affiliations:** 1 Department of Agricultural Microbiology, Faculty of Agriculture, Zagazig University, Zagazig, Egypt; 2 Department of Biochemistry, Faculty of Agriculture, Zagazig University, Zagazig, Egypt; 3 Nutrition and Food Sciences Department, National Research Centre, Giza, Egypt; 4 Molecular Cell Biology Unit, Division of Biochemistry, Department of Chemistry, Faculty of Science, Tanta University, Tanta, Egypt; 5 Soils and Water Department, Faculty of Agriculture, Fayoum University, Fayoum, Egypt; 6 Department of Biology, College of Science, King Khalid University, Abha, Saudi Arabia; 7 Department of Biology, College of Science, Imam Mohammad Ibn Saud Islamic University (IMSIU), Riyadh, Saudi Arabia; 8 Plant Production Department (Horticulture-Pomology), Faculty of Agriculture, Saba Basha, Alexandria University, Alexandria, Egypt; 9 Department of Biology, College of Science, United Arab Emirates University, Al Ain, United Arab Emirates

**Keywords:** extraction techniques, feed additives, health prospects, immune system, natural preservatives, phytochemicals, phytocompounds, plant-derived bioactive compounds

## Abstract

In recent years, there has been a growing awareness of the importance of a nutritious diet for maintaining overall health. Among dietary components, plant-derived bioactive compounds have garnered significant attention due to their functional properties and potential to prevent various diseases. These bioactive constituents, although typically present in small quantities, provide substantial health benefits and are considered non-nutritive yet physiologically active components of the diet. Medicinal plants, vegetables, fruits, cereals, sauces, and spices have become focal points in nutritional research, owing to their diverse array of bioactive compounds. These compounds, including polyphenols, glucosinolates, carotenoids, terpenoids, alkaloids, saponins, vitamins, and dietary fibers, are increasingly recognized for their ability to reduce the risk of chronic diseases, as demonstrated by epidemiological studies. These molecules exhibit a broad spectrum of therapeutic activities, including antioxidant, anti-inflammatory, anti-atherogenic, antimicrobial, antithrombotic, cardioprotective, and vasodilatory activities. Despite their promising pharmacological and nutritional potential, the integration of plant-derived bioactive compounds into commercial products remains limited. Importantly, bioactive compounds that possess antioxidant and antimicrobial activities are increasingly acknowledged for their potential application as natural and environmentally sustainable food preservatives. The expanding interest in these applications underscores the critical need for efficient and standardized extraction methods. While conventional extraction techniques have been widely used, they often suffer from limitations such as low yield, degradation of heat-sensitive compounds, and high solvent consumption. To address these challenges, innovative and integrated extraction technologies have been developed, offering advantages such as enhanced extraction efficiency, reduced impurities, and lower environmental impact. These methods often employ reduced solvent use and energy input, aligning with sustainability goals. This review aims to provide a comprehensive overview of bioactive plant compounds by examining their extraction methods, biological and immunological activities, nutritional significance, food applications, and health benefits for humans.

## 1 Introduction

With growing recognition of nutrition as a cornerstone of human health, dietary patterns have emerged as a critical area of research within the global food industry (1). Over recent decades, substantial evidence has established a strong correlation between diet and overall wellbeing, prompting modern consumers to adopt more health-conscious and preventive dietary choices (1). This shift in consumption patterns largely stems from heightened health awareness and a growing demand for an enhanced quality of life (1, 2). This growing interest has catalyzed numerous studies aimed at improving the nutritional quality of foods and investigating the potential benefits of incorporating novel bioactive compounds with targeted functional properties (1, 2). Accumulating scientific evidence indicates that chronic stress, in combination with unhealthy lifestyle choices, can synergistically impair immune function (2). This immunosuppression increases susceptibility to infectious diseases, malignancies, cardiovascular disorders, and a range of chronic health conditions (2).

Consequently, healthcare professionals, food manufacturers, researchers, and consumers alike are increasingly focused on the therapeutic potential of specific dietary components (3). In today's health-conscious society, the timeless adage, “Let food be thy medicine and medicine be thy food,” resonates more strongly than ever, emphasizing the integral role of nutrition in both disease prevention and healing (3). As public awareness of the relationship between nutrition and health continues to increase, there has been a substantial growth in the global market for nutraceuticals and functional foods (4).

Various bioactive compounds have been incorporated into functional foods, nutraceuticals, and pharmaceuticals due to their antimicrobial characteristics and humoral and cell-mediated immune functions, aiding disease prevention and control (5). Functional foods originated in 1980 when Japan's Ministry of Health and Welfare established nutritional criteria for foods with health-enhancing properties (6). These foods are classified as “functional” when they demonstrate scientifically validated benefits that extend beyond basic nutrition, targeting specific physiological functions to improve overall health and reduce disease risk (7, 8).

Functional foods have grown in global demand as consumers increasingly integrate them into regular dietary patterns (9). Market assessments forecast significant growth in this area, with the functional food business anticipated to increase from USD 161.99 billion in 2020 to USD 228.79 billion by 2025, reflecting a compound annual growth rate (CAGR) of almost 8% (9). In both scientific literature and commercial discourse, these products are often referred to interchangeably as “natural health products” or “healthy foods,” reflecting their dual roles in nutrition and disease prevention (10).

Functional foods encompass both natural and processed food products that deliver clinically demonstrated health benefits extending beyond their fundamental nutritional value (11). Historically, phytotherapeutics have been utilized for the treatment of a wide range of ailments across various cultures (11). According to the World Health Organization (WHO), approximately 80% of the global population still relies primarily on traditional medicinal practices for primary healthcare needs. Within this context, plant-derived bioactive compounds are recognized as vital contributors to preventive health strategies (12). The plant kingdom represents a vast reservoir of biologically active molecules, including terpenes, polyphenols, limonoids, carotenoids, and saponins, each exhibiting diverse therapeutic properties, such as antioxidant, anti-inflammatory, antimicrobial, and anticancer activities (12, 13).

Traditionally consumed foods such as cereal grains, millets, fruits, vegetables, spices, and condiments are rich sources of functional compounds that confer physiological benefits beyond basic nutritional requirements (14). Plants synthesize diverse bioactive compounds capable of exerting pharmacological or toxicological effects in humans and animals (15). These phytochemicals, encompassing terpenoids, alkaloids, nitrogenous compounds, organosulfur derivatives, and phenolic compounds, are widely distributed across different plant tissues (16). Extensive research has demonstrated their therapeutic potential, with documented health benefits including enhanced circulatory and digestive functions, as well as anti-inflammatory, antineoplastic, and antidiabetic effects (17).

Epidemiological studies indicate that regular consumption of natural functional foods, particularly fruits, whole grains, and vegetables rich in bioactive phytochemicals, is associated with a significantly reduced risk of chronic diseases, including cancer, metabolic syndrome, type 2 diabetes, obesity, and cardiovascular disorders (18, 19). Protective effects are attributed to key bioactive compounds such as ellagic acid, resveratrol, anthocyanins, epigallocatechin, oleuropein, curcumin, sulforaphane, and quercetin (20). Furthermore, plant-derived bioactive molecules with antioxidant and antimicrobial properties offer a sustainable alternative to synthetic food preservatives (21).

The global agro-industry generates vast raw materials, primarily for energy production and human or animal consumption (22). The agri-food processing industry generates more than 190 million tons of by-products yearly, including plant leaves, seed residues, fruit pomaces, cereal brans, fruit skins, and oilseed meals (23). Effective management of these by-products, through recycling, disposal, or valorization, is critical for sustainable practices (24, 25). Many food by-products hold significant economic potential due to their composition and abundance (26). For instance, they can serve as raw materials for extracting functional food ingredients, aligning with current market trends (27). Rich in lipids, carbohydrates, fiber, vitamins, and phenolics, these by-products offer versatile applications (28, 29). The application of bioactive compounds could enhance economic viability, environmental sustainability, and global food security (23).

Plant-derived bioactive compounds present distinct advantages over animal-based alternatives, including higher abundance, cholesterol-free profiles, suitability for vegetarian markets, and cost-effectiveness (30). Consequently, scientific interest in extracting bioactive compounds from plant-derived by-products has grown significantly across developed and developing nations (31, 32).

Optimizing extraction methodologies for bioactive phytochemicals necessitates careful evaluation, particularly for food and pharmaceutical applications, as the selected technique plays a pivotal role in preserving the functional properties, sensory characteristics, and nutritional quality of the target compounds (33). Conventional chemical extraction approaches raise significant concerns, including potential safety hazards, excessive energy consumption, suboptimal product purity, environmental contamination, and toxicological consequences (34). Consequently, there is a pressing need to develop efficient and optimized extraction protocols to maximize the recovery of bioactive compounds. This is particularly important for plant-derived phytochemicals, where the presence of a rigid cell wall matrix can significantly hinder extraction efficiency and yield (34).

Emerging extraction technologies have enabled novel approaches that significantly improve both the yield and accessibility of bioactive compound recovery (35). Environmentally sustainable methods such as ultrasound-assisted extraction (UAE), microwave-assisted extraction (MAE), and pulsed electric field extraction (PEFE) now enable the production of high-quality plant extracts while minimizing environmental impact (36).

**Figure 1:**
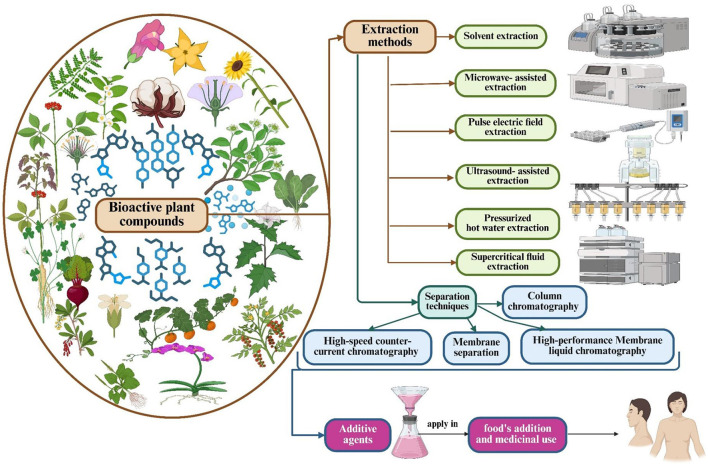
Innovative extraction methods for isolating bioactive compounds from plant by-products for applications in food additives and medicine.

Table 1 illustrates the current conventional and emerging methods for extracting bioactive compounds from plants. Figure 1 depicts advanced novel extraction procedures for isolating bioactive chemicals from plant by-products for applications in food additives and medicine.

**Table 1:** Comparison of conventional and novel emerging extraction technologies for isolating bioactive compounds from plant sources.

**Extraction technique**	**Category**	**Plant extract (bioactive compounds)**	**References**
**Conventional techniques**			
Soxhlet extraction	Conventional	Orange peel (β-carotene)	(248)
		Kinnow peels (flavonoids)	(603)
		Grape peels (catechin, rutin, and epicatechin)	(251)
		Grape skin (anthocyanins)	(249)
		*Arbutus unedo* fruit (catechin)	(253)
**Novel techniques**			
Ultrasound-assisted extraction (UAE)	Novel	Purple eggplant peel (carotenoids)	(604)
		Goji berry peels (gallic acid)	(605)
		Citrus peel (hesperidin, neohesperidin, tangeritin, and diosmin)	(606)
		Jujube peels (quercetin 3-β-D-glucoside, rutin, and kaempferol-3-O-rutinoside)	(607)
		Dragon peel (betacyanin)	(608)
		Plum and grape peels (anthocyanins)	(609)
		Ginger herbal dust (8-gingerol, 6-gingerol, and 6-shogaol)	(610)
		Tomatoes (lycopene)	(611)
		Onion wastes (quercetin)	(531)
		Artichoke leaves (chlorogenic acid)	(612)
		Spinach leaves (β-carotene and lutein)	(613)
Infrared-assisted extraction (IRAE)	Novel	Mango leaves (iso-quercitrin, quercitrin, and rutin)	(348)
		Olive leaves (hydroxytyrosol and oleuropein)	(352)
		*Scutellariae radix* (Wogonin, oroxylin A, and baicalein)	(349)
		Apricot pomace (catechin, epicatechin, and rutin)	(350)
		*Salviae miltiorrhizae* (danshen) (phenolic compounds such as protocatechuic acid, salvianolic acid B, danshensu; aldehyde derivative; diterpenoid quinones such as cryptotanshinone, dihydrotanshinone, tanshinones I/IIA	(351)
		Orange peels (polyphenols)	(614)
Microwave-assisted extraction (MAE)	Novel	*Aloysia citrodora* (phenylpropanoids, iridoids, and flavonoids), which are valuable for their antioxidant and health-promoting properties	(615)
		Cherry pomace (syringic acid, vanillic acid, epicatechin, gentisic acid, and quercetin). These enhance the extract's antioxidant capacity and functional value	(616)
		Asian plants (*Quercus infectoria, Commiphora mukul*, and *Cinnamomum verum*) (tannin and cinnamaldehyde). These contribute potent antioxidant and antimicrobial activities	(617)
		Grape waste (polyphenols and tannins). These are compounds known for their potent antioxidant effects and potential to improve food preservation	(618)
		*Opuntia ficus-indica* (betalain). This is a pigment with significant antioxidant and anti-inflammatory potential	(619)
		Grape peels (anthocyanin). This is a natural colorant with powerful antioxidant properties	(620)
		Mango seeds [pent-O-galloyl-glucoside (PGG)], ethyl gallate, and hamnetin-3-[6-2-butenoilhexoside]. All of which enhance the extract's antioxidant and antimicrobial functions	(621)
		Avocado seeds (catechin, procyanidins dimer B, and epicatechin). These are bioactive compounds that contribute to antioxidant and cardiovascular benefits	(622)
Pulsed electric field extraction (PEFE)	Novel	Rapeseed stems (proteins and polyphenols). These improve nutritional value and antioxidant activity	(623)
		Grape peels and plum (flavonoids, anthocyanins, and phenols). These provide antioxidant, anti-inflammatory, and color-enhancing effects	(609)
		Apple peels (rich in total phenolic acids). These enhance antioxidant capacity	(624)
		Papaya seeds (proteins, carbohydrates, total phenolic acids, and isothiocyanates). These enhance both nutritional and functional properties	(625)
		Pear peel (betanin and isobetanin, pigments). These have antioxidant and health-promoting effects	(626)
		Orange peel (hesperidin and naringin). There are flavonoids with antioxidant and anti-inflammatory activities	(627)
		Grape by-products (anthocyanins). These are natural pigment with strong antioxidant capacity	(628)
Pressurized liquid extraction (PLE)	Novel	Citrus peel waste (p-coumaric acid, rutin, t-ferulic acid, and hesperidin). These all enhance antioxidant and health benefits	(629)
		Waxy barley (β-glucans and phenols). These improve both nutritional quality and antioxidant activity	(630)
		*Phyllanthus niruri* (corilagin and tannin). These bioactive compounds are known for their antioxidant and therapeutic effects	(631)
		*Phyllanthus tenellus* (hydrolysable tannins)	(632)
Supercritical fluid extraction (SFE)	Novel	Apple seed oil (tocopherol). This is a potent antioxidant that enhances oil stability	(633)
		Sage herbal dust extracts (monoterpenes). These contribute to aroma and potential antimicrobial activity	(634)
		Elderberry pomace (total phenolic acids). These boost antioxidant capacity	(635)
		Pomegranate seed (total phenolic acids). These enhance antioxidant and health-promoting properties	(636)
		Potato peels extract (mixture of gallic, chlorogenic, caffeic, protocatechuic, syringic, p-hydroxybenzoic, ferulic, and coumaric acids (o- and p-isomers). These contribute to potent antioxidant and protective effects	(637)
		Winery by-products (total polyphenols and flavonoids). These improve antioxidant potential and functional value	(638)
		Haskap berry pulp (anthocyanins). These are natural pigments with significant antioxidant activity	(639)
		Pomegranate seeds (punicic acid). This is a bioactive fatty acid with anti-inflammatory and health benefits	(640)
		Brazilian cherry extracts (germacrene, terpene, and γ-element). These are compounds known for their aroma and potential bioactivity	(641)
		Strawberry seeds (flavanols and ellagitannins). These enhance antioxidant and health-promoting properties	(642)
Subcritical water extraction (SWE)	Novel	Wheat straw (phenolic compounds). These enhance antioxidant activity	(643)
		Papaya seeds (vanillic acids, ferulic, and mandelic acids). These contribute to antioxidant and antimicrobial functions	(644)
		Sweet passion fruit seeds (tocopherols). This provides antioxidant protection	(645)
		Grape pomace (polyphenols). These support antioxidant capacity and food preservation	(646)
Enzyme-assisted extraction (EAE)	Novel	Tomatoes (lycopene). This is a carotenoid with potent antioxidant and health-promoting properties	(356)
		Bay leaves (essential oils)	(357)
		Turmeric (curcumin)	(358)
		Grape peels (pectin)	(359)
		Passion fruit peels (pectin)	(360)
		Pineapple peel extract (gallic acid, catechin, epicatechin, and ferulic acid)	(361)

This review distinguishes itself from existing literature through its unprecedented breadth and depth, integrating six interrelated domains, extraction methodologies, biological activities, immunological functions, nutritional aspects, food applications, and human health benefits into a single comprehensive framework. Unlike prior reviews that typically focus on one or two facets, the present review systematically compares both traditional and cutting-edge extraction techniques, including natural deep eutectic solvents (NADES), ionic liquid extraction (ILE), and hybrid approaches, while also addressing critical regulatory challenges and evaluating economic costs.

By encompassing a temporal scope from 2000 to 2025 and employing rigorous search strategies across PubMed, Scopus, Google Scholar, and Web of Science, the current review provides historical context and captures the latest advances in artificial intelligence (AI)–driven process optimization, biorefinery integration, and nanotechnology-enhanced delivery systems. Furthermore, this work fills notable methodological gaps: it proposes standardized extraction protocols and characterization methods to address the lack of comparability in current studies, emphasizes industrial scalability and commercial viability often overlooked in academic reviews, and rigorously evaluates environmental sustainability through life cycle assessments.

In contrast to single-compound or single-method analyses prevalent in the literature, the present review offers a holistic examination of bioactive compound extraction and application. It also uniquely highlights regulatory harmonization efforts, delving into divergent global frameworks and providing practical guidance for navigating pre-market approvals, safety assessments, and label-claim substantiation.

This review identifies thirteen future research priorities, including the optimization of hybrid extraction technology, regulatory convergence, AI-driven parameter tuning, and circular economy models, thereby combining existing knowledge and providing a clear roadmap for advancing the field. This review comprehensively examines bioactive compounds derived from various plant sources and their byproducts, focusing on extraction methodologies, health benefits, potential applications, and current technological limitations.

## 2 Methodology

This study provides a comprehensive examination of the pertinent and contemporary literature on bioactive plant compounds, encompassing their classifications, extraction techniques, biological activities, immunological considerations, nutritional properties, and health benefits for humans. To ensure consistency and reproducibility, the precise search approach was used consistently across different databases, including PubMed, Scopus, Google Scholar, and Web of Science.

For PubMed, Scopus, and Web of Science, the following search terms and Boolean operators were used: “bioactive compounds” in combination with terms such as “plant sources,” “agri-food by-products,” “extraction,” “bioactivities,” “health benefits,” “food applications,” and “limitations.” Titles, keywords, and abstracts were initially evaluated for relevance. Full-text publications were then retrieved and included if they were deemed appropriate for an objective and comprehensive evaluation.

For Google Scholar, the search was performed using the same criteria, but without the ability to apply precise filters such as language or date range. Therefore, the results were manually adjusted based on relevance and date.

The present review utilized peer-reviewed articles and reviews, with a date range spanning from 2000 to 2025, modified according to database specifications.

## 3 Bioactive compounds derived from plant by-products

Comprehensive studies have focused on identifying the diverse array of bioactive substances found in fruit and vegetable peels, which are increasingly acknowledged as significant sources of antioxidants, dietary fiber, polyphenols, and other health-enhancing phytochemicals (37–39). Analytical investigations have revealed that phenolic compound levels in fruit peels, specifically from papaya (*Carica papaya*), passion fruit (*Passiflora edulis*), and pomegranate (*Punica granatum*), consistently reach approximately double the concentrations present in their respective seeds and pulp (33). Comparative analyses indicate that papaya peels exhibit particularly favorable nutritional profiles, characterized by higher mineral content, elevated levels of ascorbic acid, and greater antioxidant activity compared to seeds. These nutritional advantages have been consistently observed across various cultivated papaya varieties (33).

Phytochemical investigations have identified six predominant flavonoid compounds in papaya peel and leaf extracts: myricetin, quercetin, kaempferol, morin, apigenin, and luteolin (33). Comparative phytochemical analyses have revealed that mango (*Mangifera indica*) peels contain significantly higher concentrations of phenolic acids, particularly gallic acid, and flavonoid compounds such as quercetin, compared to the peels of other fruit species (40). This trend is consistent among tropical fruits, as demonstrated by Sultana et al. (41). Peels of tropical fruits exhibit significantly higher concentrations of gallotannins and total phenolics compared to their corresponding pulp tissues, with mango, mangosteen, and dragon fruit showing particularly pronounced differences (41).

Wolfe et al. (42) conducted a comprehensive analysis of apple (*Malus domestica*) phytochemicals, revealing that peel tissues contain 3–5 times higher concentrations of flavonoids (particularly quercetin glycosides) and phenolic acids (including chlorogenic acid) than flesh tissues. This pattern is even more pronounced in citrus fruits, where peel phenolic content reaches exceptional levels of 4,500–5,000 mg/g dry weight, approximately 10–15 times greater than concentrations measured in edible portions (43). Citrus fruits (*Citrus* spp.) are rich in two primary classes of bioactive compounds: phenolic derivatives, such as flavonoids and phenolic acids, and terpenoids, including limonoids and carotenoids (44).

Comparative phytochemical analyses have shown that vegetable peels contain higher concentrations of bioactive compounds than their corresponding edible tissues, reflecting similar trends observed in fruit by-products (33, 45). Vegetable processing, particularly of crops such as tomatoes and eggplants, generates substantial waste, with peels and seeds comprising approximately 40%−60% of the total by-products (33). A sustainable strategy for managing this waste involves recovering high-value bioactive compounds (33).

For example, lycopene can be efficiently extracted from tomato peels, while other vegetable residues serve as rich sources of proteins, pigments, dietary fibers, carotenoids, and organic acids, offering significant potential for use in food, nutraceutical, and pharmaceutical applications (33). This method reduces waste accumulation and recovers nutritionally beneficial components for potential reuse (33). Furthermore, lemon seeds were found to possess a diverse flavonoid profile, containing notable levels of gallocatechin, caffeic acid, epicatechin, vitexin, quercetin, and hesperidin (40).

According to Ravichandran et al. (46), the peels of root vegetables, such as beetroot and carrot, are rich sources of bioactive polyphenols, including betagarin, betavulgarin, and cochliophilin A, as well as betalain pigments like betacyanin and betaxanthin. These compounds exhibit potent antioxidant activity, highlighting the nutritional potential of vegetable byproducts (46). According to Cartea et al. (47), cruciferous vegetables, especially broccoli and cauliflower, are rich in bioactive compounds such as dietary fiber, ascorbic acid, flavonoid derivatives (e.g., quercetin, kaempferol, and isorhamnetin), and phenolic acids (including p-coumaric, sinapic, and ferulic acids).

**Figure 2:**
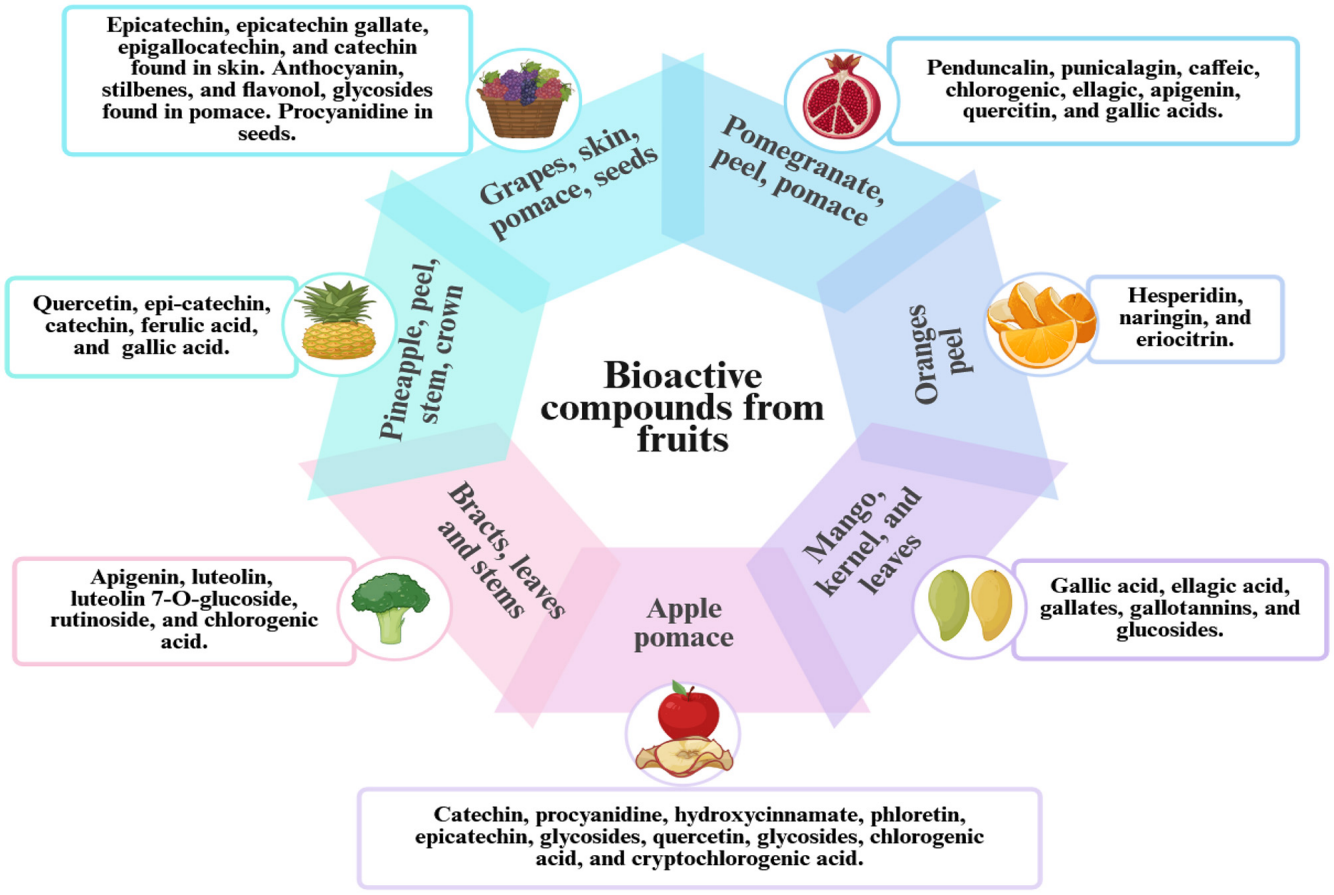
Bioactive compounds derived from fruits and their associated functional properties.

Additionally, studies have shown that lyophilized potato peel extracts demonstrate significant antioxidant activity in various *in vitro* assays (48). Research has shown that phenolic and flavonoid compounds in cucurbitaceae fruit peels effectively reduce lipid peroxidation, as reported by Rajasree et al. (45). Olive leaves contain valuable bioactive compounds, including rutin, tyrosol, hydroxytyrosol, and oleuropein, making them a rich source of beneficial metabolites (49). Similarly, analytical characterization in a recent study (50) demonstrated that olive leaves are a rich source of diverse phenolic compounds, including rutin, tyrosol, luteolin, quercetin, p-coumaric acid, ferulic acid, and caffeic acid (50). Figure 2 illustrates the bioactive compounds obtained from fruits and their corresponding functional properties.

## 4 Plant-based bioactive constituents

Plant-based functional foods are derived from natural or processed plant sources and contain known and unknown bioactive compounds (51). Functional foods can be systematically classified into six principal categories according to their dominant bioactive components: steroidal saponins, polyphenols, flavonoids, alkaloids, polysaccharides, and miscellaneous phytochemicals (52). Their elevated concentrations of bioactive constituents and demonstrated health-promoting properties have driven substantial growth in consumption patterns in recent years (53).

Representative examples encompass oats, citrus fruits (e.g., oranges), grapes, soybeans, garlic, flaxseed, tomatoes, tea, and cruciferous vegetables (e.g., broccoli). These functional foods play a vital role in promoting health, maintaining physiological homeostasis, and reducing the risk of diseases associated with phytochemical imbalances or deficiencies (54). Research on plant-based functional foods with immune-enhancing properties has gained considerable attention, driven by growing public awareness of their disease-preventive potential. As a result, an increasing number of individuals are incorporating these foods into their diets to strengthen immune function and support overall health (55). The populace has attained an enhanced quality of life through consuming vegetables, fruits, and other plant-based foods (56).

**Figure 3:**
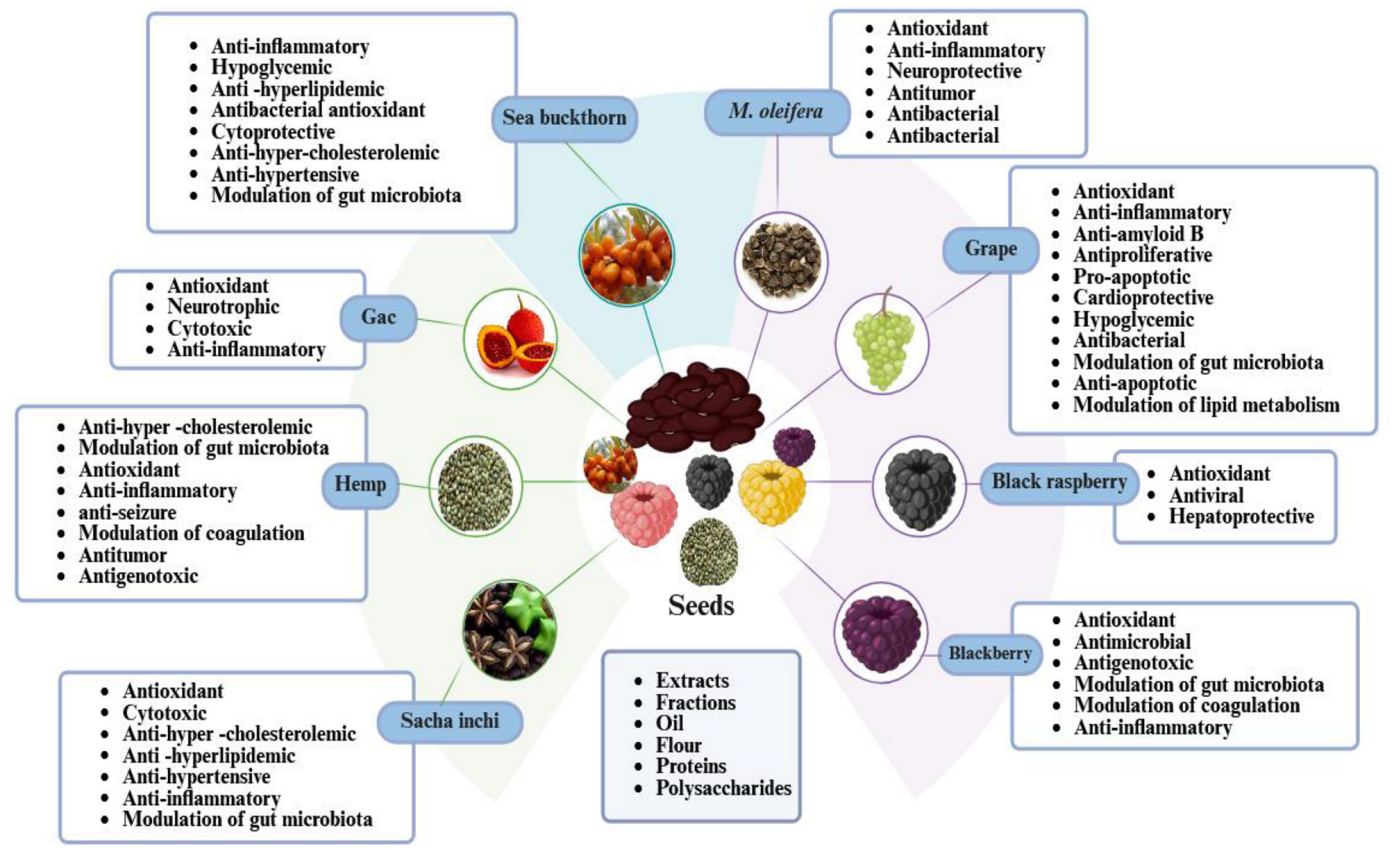
Seed bioactive components have antioxidants, anti-inflammatory, antibacterial, and cardioprotective characteristics.

Figure 3 illustrates seeds as reservoirs of bioactive compounds that demonstrate a variety of biological activities, encompassing antioxidant, anti-inflammatory, antibacterial, and cardioprotective properties.

### 4.1 Spices and condiments

Extensive phytochemical analyses have elucidated the bioactive metabolites present in culinary spices and condiments, particularly alkaloids, flavonoids, and tannins (57). A central objective within this research domain is the characterization of physiologically active constituents in functional foods. Notably, organosulfur compounds derived from garlic (*Allium sativum*) have emerged as key bioactive agents, with well-documented efficacy in lowering low-density lipoprotein (LDL) cholesterol, alleviating hypertension, and contributing to blood pressure regulation (58). Curcumin, the principal bioactive compound in turmeric (*Curcuma longa*), exhibits a wide range of biological activities, including antimicrobial, detoxifying, tonic, and antacid effects. Its mechanisms of action have been extensively characterized through studies involving protein expression profiling and molecular pathway analysis (59, 60).

Cuminaldehyde, the primary bioactive compound in cumin (*Cuminum cyminum*), exhibits a broad spectrum of physiological and therapeutic properties (61). These include lactogenic effects, enhancement of gastrointestinal function, stimulation of appetite, and modulation of taste perception. Clinically, it has been employed in the management of various conditions such as abdominal distension, fluid retention (edema), fever (pyrexia), gastrointestinal disturbances, including nausea, vomiting, and diarrhea, as well as anorexia and postnatal recovery (61, 62). Similarly, clove (*Syzygium aromaticum*) is rich in bioactive constituents, notably eugenol and eugenyl acetate, both of which exhibit potent natural antioxidant properties (63, 64).

Similarly, black pepper (*Piper nigrum*) yields piperine, a bioactive alkaloid with established anti-inflammatory, antioxidant, and chemopreventive properties. Nutmeg (*Myristica fragrans*) contains abundant antioxidant compounds, including flavonoid derivatives, terpenoids, and hydrolyzable tannins (65). Fenugreek (*Trigonella foenum-graecum*) contains valuable phytochemicals, including vitexin, kaempferol, and quercetin, which exhibit analgesic and antidiabetic effects. Zhang et al. (66) employed a series of *in vitro* bioassays to evaluate the functional food potential of coriander (*Coriandrum sativum*) seeds. Their study not only confirmed the nutraceutical value of the seeds but also pioneered a comprehensive phytochemical characterization of roasted coriander specimens. The findings substantiate the classification of roasted coriander as a functional food, supported by systematic bioactivity profiling.

### 4.2 Medicinal plants

These kinds of plants are widely acknowledged for their significant pharmacological potential and long-standing role in traditional and modern therapeutic practices worldwide (67). They exhibit potent antimicrobial activity against pathogenic bacteria, glucose-lowering effects in diabetes management, and clinically significant anti-hyperglycemic and anti-hyperlipidemic properties (67). These botanicals are primarily utilized in preventive healthcare, targeting a broad spectrum of conditions ranging from common infections to chronic diseases, such as cancer. Unlike staple dietary components, their use is typically focused on therapeutic or functional purposes rather than routine nutritional intake (68).

Different plant organs, including stems, roots, leaves, flowers, bark, and fruits, contain abundant bioactive compounds such as phenolic acids (rosmarinic, caffeic, carnosic), flavonoids (quercetin, kaempferol, and luteolin glycosides), terpenoids (oleanolic acid and triterpenoids), and specialized metabolites (anthraquinones, alkaloids, emodin, and eugenol), which have been validated as effective nutraceutical additives (69). Furthermore, plant-derived extracts offer substantial potential for promoting sustainable food production systems, while enhancing ecological and socioeconomic benefits through their application in functional food development (70). Despite these advances, additional research is required to investigate: (1) human sensory perception, (2) novel sources of antimicrobial and antioxidant compounds, (3) optimal extraction parameters, and (4) fundamental mechanisms governing food preservation (71). Rosemary (*Rosmarinus officinalis*) extracts demonstrate versatile applications across multiple domains, including plant-based nutrition, pharmaceutical formulations, functional foods, and natural food preservation systems (72).

As an abundant, economical, and safe botanical resource, rosemary facilitates the commercial adoption of its essential oils and phenolic-rich extracts within the food industry (73, 74). Rosemary (*R. officinalis*) has demonstrated significant antimicrobial activity in various meat products, including cooked beef (75), sausage (76, 77), and beef meatballs (78, 79). Although medicinal plants exhibit well-documented antimicrobial and antioxidant properties with proven health benefits, their use as natural food preservatives remains relatively underexplored compared to other botanically derived sources, such as fruits, vegetables, herbs, and spices, which share similar phytochemical profiles (80). Research indicates that the functional properties of plant materials are attributed mainly to their bioactive constituents, particularly terpenes and phenolic compounds, which play key roles in antioxidant, antimicrobial, and anti-inflammatory activities (81, 82).

### 4.3 Fruits

They are universally acknowledged as prototypical functional foods, owing to their abundant concentrations of bioactive constituents, notably soluble dietary fiber, antioxidant phytochemicals, essential minerals, and vitamins (particularly ascorbic acid, retinol equivalents, and tocopherols) (83). The polyphenolic composition of fruits predominantly comprises two principal classes: (i) non-flavonoid derivatives, including lignans, hydroxycinnamic/phenolic acids, and stilbenes and (ii) flavonoid subclasses, encompassing flavones, flavonols, flavanones, isoflavones, and anthocyanin pigments (84, 85).

Several countries, including the United States, Poland, and New Zealand, have successfully commercialized fruit-based functional beverages, capitalizing on the natural health benefits, sensory appeal, and perceived freshness of their botanical ingredients. These products incorporate a diverse array of fruit varieties, spanning pomaceous fruits (apples), stone fruits (mangoes, peaches, plums, and cherries), berries (blueberries, strawberries, blackcurrants, and cranberries), tropical species (açaí, acerola, kiwifruit, and guarana), and vine crops (grapes and pomegranates) (86, 87). Mango (*Mangifera indica*), often referred to as the “king of fruits,” contains a rich and diverse profile of polyphenolic compounds distributed throughout its various anatomical parts, including the seed, bark, pulp, leaves, peel, and floral tissues, each contributing to its bioactive potential (88). Notably, the xanthonoid mangiferin exhibits exceptional antioxidant capacity and broad-spectrum therapeutic potential. Litchi (*Litchi chinensis*) similarly qualifies as a functional food, with established anti-neoplastic activity demonstrated through both *in vitro* and *in vivo* investigations (89, 90). Meanwhile, globally cultivated peanuts (*Arachis hypogaea*) serve as nutrient-dense ingredients in processed foods, containing essential vitamins, proteins, dietary fiber, and bioactive phytochemicals (91), including phenolic acids, flavonoids, resveratrol, and phytosterols, compounds that have been clinically shown to inhibit intestinal cholesterol absorption (92, 93).

Jujube (*Ziziphus jujuba*) fruit is widely consumed worldwide as both a traditional food and a functional ingredient. It can be eaten fresh as pulp or processed into various food products, including beverages, pickles, compotes, jams, and jellies (94). Additionally, the dried pulp is a versatile functional ingredient in the food industry, incorporated into baked goods (bread, cakes), snacks, Chinese dates, and tea blends. According to Cai et al. (94), *Ziziphus mauritiana* (Indian jujube) fruit juice is rich in phenolic compounds and essential nutrients, underscoring its strong potential as a functional food ingredient. Deng et al. (95) investigated the health benefits of soluble dietary fiber derived from *Rhodomyrtus tomentosa* fruits, highlighting its potential as a functional food component. Their findings suggest that this dietary fiber may inhibit the accumulation of advanced glycation end-products (AGEs) in the body, thereby exerting protective effects against AGE-associated diseases, such as diabetes, cardiovascular disorders, and neurodegenerative conditions (95).

### 4.4 Cereals

Cereals provide 60%−70% of the global daily energy intake and are consumed in whole, processed products, and fully refined derivatives (96). Certain varieties, such as colored rice, maize, wheat, and specific millets, are abundant in bioactive compounds like polyphenols, tocopherols, and antioxidants (97). These functional components play a crucial role in regulating and preventing chronic diseases, including cardiovascular disorders, hypertension, and type 2 diabetes. Furthermore, whole grain cereals have been associated with reduced cancer risk, improved blood pressure regulation, and enhanced glycemic control, contributing to overall chronic disease prevention (98).

Cereals and their derivatives are gaining recognition as functional foods because they provide vital nutrients, including vitamins, minerals, energy-yielding compounds, antioxidants, and dietary fiber (99). Prominent dietary fibers such as β-glucan and arabinoxylan exhibit significant health benefits. As a soluble fiber, β-glucan enhances fluid viscosity, potentially promoting small intestinal fermentation, delaying gastric emptying, slowing intestinal transit, and increasing luminal viscosity (100, 101). Additionally, cereals serve as an optimal fermentable substrate for probiotic bacteria, fostering their proliferation and metabolic activity (102, 103).

These grains are also rich in bioactive compounds, including vitamin E, linoleic acid, dietary fiber, selenium, folate, and phenolic acids, which confer antioxidant properties and may reduce the risk of coronary heart disease (98, 104). Certain cereal-based functional foods not only enhance dietary nutrition but also offer weaning benefits, along with probiotic and prebiotic advantages (97). According to Bora et al. (105), millets are particularly notable for their hypoglycemic effects and dense nutritional profile, positioning them as promising candidates for the development of functional foods (105).

### 4.5 Vegetables

Vegetables are a cornerstone of a balanced diet, providing essential macronutrients and micronutrients, including vitamins, minerals, and dietary fiber, that are critical for maintaining optimal health and preventing nutrient-related disorders (106). Due to their seasonal nature, market demands, and consumer preferences, most vegetables undergo processing, resulting in significant byproducts (107).

As an essential component of a balanced diet, vegetables are rich in diverse bioactive compounds, including polyphenols, glucosinolates, and carotenoids, which have been extensively associated with the prevention and risk reduction of chronic diseases such as cardiovascular disorders, type 2 diabetes, and various forms of cancer (108). Vegetable processing byproducts are a rich source of valuable nutrients, encompassing lipids, proteins, carbohydrates, fiber, essential oils, and bioactive compounds such as flavonoids and phenolics (109). These bioactive constituents frequently exhibit therapeutic properties, such as antibacterial, anti-inflammatory, and antioxidant activities, rendering them potentially effective in the prevention and management of gut-related disorders, including dysbiosis and immune-mediated inflammatory conditions (110). Certain fruits like tomatoes—commonly classified as vegetables in culinary use—contain lycopene as their primary pigment. This compound has shown significant anticancer effects and ranks among the most potent biological antioxidants (111, 112). Similarly, studies on okra (*Abelmoschus esculentus*) have identified beneficial compounds like flavonoids and catechins, which contribute to various health benefits, including anticancer, antidiabetic, antimicrobial, and antihypertensive effects (113). Given their potential health advantages, okra and its derivatives are increasingly used as key ingredients in innovative functional foods (114).

Research on plant-based fermented foods is rapidly expanding, driven by the probiotic potential of their native microbial communities, their versatility across food and pharmaceutical industries, and their growing significance as non-dairy carriers for probiotic delivery, particularly appealing to lactose-intolerant, vegan, and health-conscious consumers (115). Unlike dairy-based options, plant-derived fermentations are suitable for lactose-intolerant individuals, those with milk allergies, or consumers following vegan diets (116). These products are particularly appealing because they provide essential nutrients, including vitamins, minerals, antioxidants, and fibers, while typically containing low sugar levels. Additionally, they may act as novel carriers for traditional dairy probiotics, offering opportunities to expand into new consumer markets (117).

## 5 Functional foods of plant origin rich in bioactive compounds

Plant bioactive compounds are typically distinguished from essential nutrients, as they are classified as secondary metabolites, non-essential for basic growth and development but crucial for plant defense and ecological interactions, and increasingly recognized for their health-promoting effects in humans (118). However, these compounds play vital ecological roles in plant defense, competition, reproduction, and signaling (119). They are often defined as plant-derived secondary metabolites that exert pharmacological or toxicological effects in humans and animals (118, 120–122).

**Figure 4:**
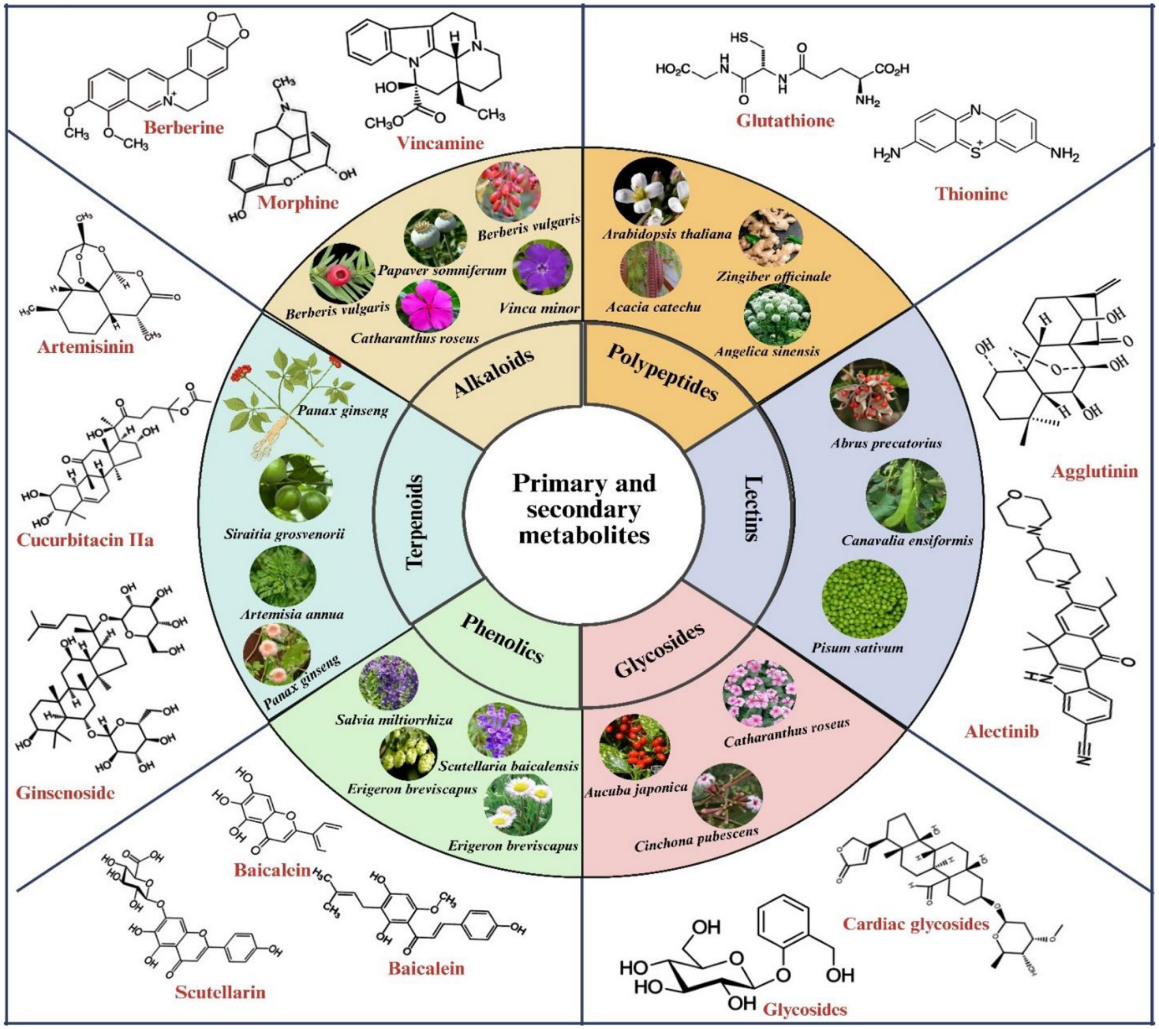
Representative chemical structures of bioactive secondary metabolites derived from medicinal plants, thoroughly categorized based on their biosynthetic origins. The major classes include: (1) phenolic compounds, such as salvianolic acids (*Salvia miltiorrhiza*), xanthohumol (*Humulus lupulus*), scutellarin (*Erigeron breviscapus*), and baicalein (*Scutellaria baicalensis*); (2) terpenoids, including ginsenosides (*Panax ginseng*), cucurbitacin IIa (*Hemsleya chinensis*), artemisinin (*Artemisia annua*), and mogroside V (*Siraitia grosvenorii*); and (3) alkaloids, such as vincristine (*Catharanthus roseus*), morphine (*Papaver somniferum*), vincamine (*Vinca minor*), berberine (*Berberis vulgaris*), and paclitaxel (*Taxus wallichiana*).

**Figure 5:**
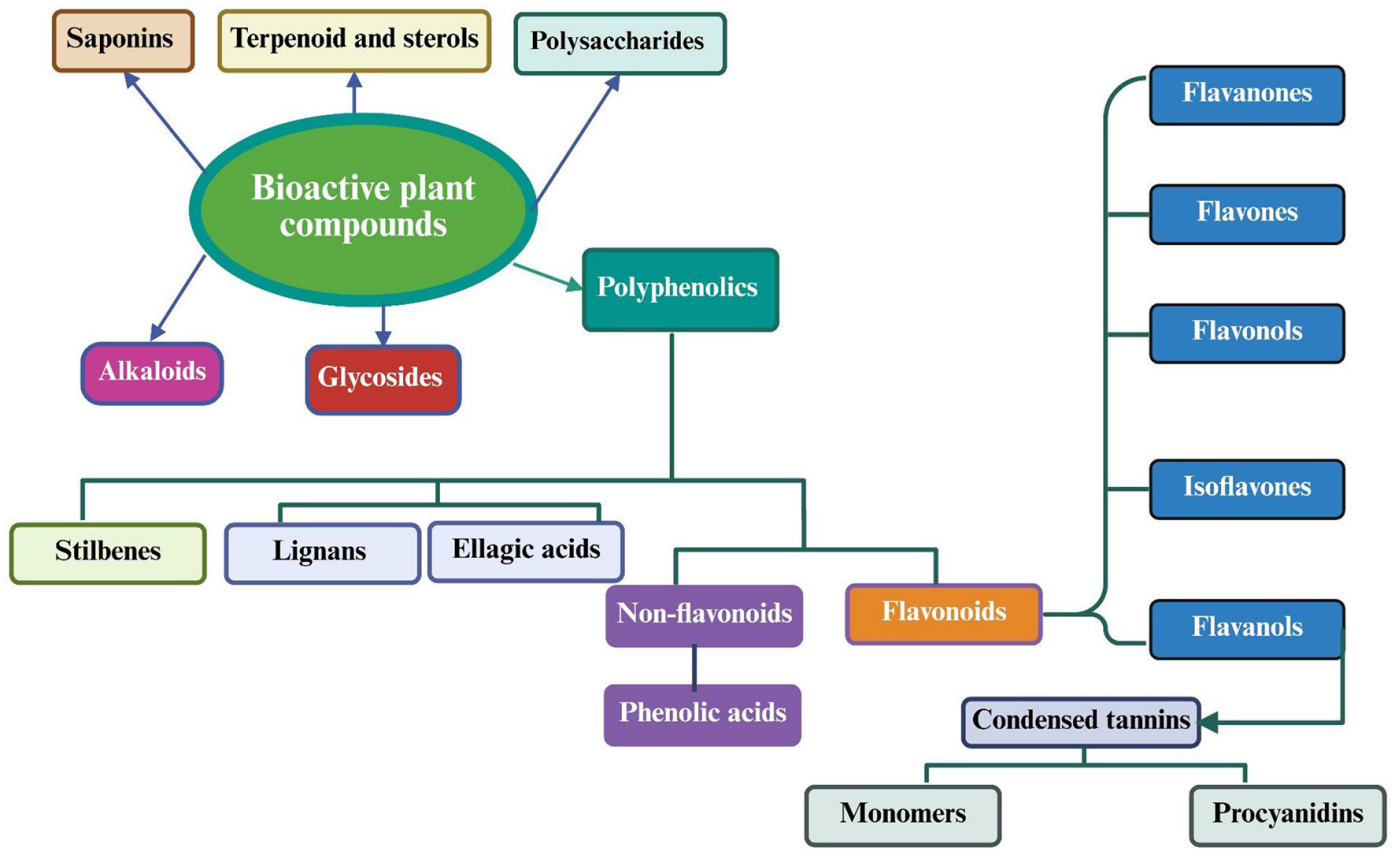
Classification of bioactive plant chemicals according to their chemical structure and biological function, encompassing polyphenols, alkaloids, terpenoids, glycosides, and organosulfur compounds.

Figures 4, 5 illustrate the primary and secondary metabolites in plant-based foods, encompassing spices, sauces, medicinal herbs, cereals, and vegetables, which substantially augment their nutritional profile. These phytochemicals encompass various chemical classes, including polysaccharides, saponins, flavonoids, alkaloids, vitamins, carotenoids, fatty acids, polyphenolic compounds, essential oils, phytosterols, and cannabinoids. Each class demonstrates unique biological activity, capable of inducing specific cellular and physiological responses that confer health benefits.

### 5.1 Polysaccharides

Plant polysaccharides are extensive polymers composed of many similar or varied monosaccharides interconnected *via* α- or β-glycosidic bonds (123). Plants synthesize a wide variety of polysaccharides, including starch, cellulose, and pectin, which exhibit substantial structural diversity in terms of molecular composition, configuration, and molecular weight across different species. This structural variability underlies their widespread distribution throughout the plant kingdom and contributes to their distinct functional roles in both plant physiology and food systems (124).

Polysaccharides include numerous bioactive compounds found in various plant-derived functional foods. Complex carbohydrates are crucial for sustaining human health and are associated with multiple health benefits (125). Extensive research has been dedicated to the extraction and characterization of polysaccharides, a significant class of biomacromolecules, due to their diverse bioactive properties and wide-ranging applications in food, pharmaceutical, and biomedical fields. Naturally derived polysaccharides are generally low in toxicity while exhibiting diverse biological activities, including antibacterial and anti-inflammatory properties (126–128). Plant polysaccharides serve as energy reserves by undergoing hydrolysis, which releases sugars that are utilized in metabolic pathways. These compounds also act as nutritional reservoirs during periods of fasting (129). Among these, starch and glycogen are recognized as the most prominent storage polysaccharides in biological systems (130).

Structural polysaccharides are complex carbohydrates that provide vital mechanical support to various biological systems (131). They help maintain structural stability within the cellular membranes of plants and animals. The two primary forms are cellulose and chitin (132). Mucopolysaccharides (also called mucilage polysaccharides) are naturally occurring compounds present in plant cell walls, cyanobacteria, and intercellular cementing substances (133). Structurally, polysaccharides are composed of various monosaccharide units and sugar derivatives, including galactose and uronic acids, which contribute to their functional diversity and bioactivity (134). A particularly important mucopolysaccharide is pectin, which is predominantly located in the cell walls and intercellular layers of fruits. Citrus peels serve as a rich source, containing 0.5%-3.5% pectin, making them valuable for jelly and jam production (135).

Chemically, pectin is composed of galactose, arabinose, galacturonic acid, and dimethyl galacturonic acid (132). Medicinal plants serve as essential sources of bioactive polysaccharides with diverse therapeutic applications (136). Key examples include *Mactra veneriformis, Acacia tortilis*, various *Dendrobium* species, *Saccharina japonica, Acanthopanax senticosus, Prunus persica*, and *Aloe barbadensis* (134). Non-starch polysaccharides support cardiovascular health by promoting the conversion of cholesterol into bile acids, a process that aids in lowering serum cholesterol levels and may subsequently reduce the risk of acute cardiovascular events (137). Additionally, polysaccharide digestion plays a crucial role in regulating blood glucose and insulin levels (138, 139).

The term “glycemic index” typically measures how quickly available carbohydrates raise blood glucose levels compared to a reference carbohydrate, such as pure glucose (140). In recent years, polysaccharides have emerged as highly promising biomaterials in biomedical applications due to their biocompatibility, versatile physicochemical properties, and biodegradability (141). An expanding body of research indicates that dietary fiber and resistant polysaccharides play a significant role in mitigating risk factors associated with chronic diseases, including cardiovascular disorders and certain types of cancer. These insights present a compelling opportunity for researchers in agricultural and food sciences to develop innovative functional food products that address the growing global burden of diet-related health conditions (142).

### 5.2 Saponins

An expanding body of research indicates that dietary fiber and resistant polysaccharides play a significant role in mitigating risk factors associated with chronic diseases, including cardiovascular disorders and certain types of cancer (143). These insights offer a compelling opportunity for researchers in agricultural and food sciences to develop innovative functional food products aimed at addressing the growing global burden of diet-related health conditions (143). Their applications are broad, ranging from natural food additives to traditional medicine and pharmaceutical uses (144). Saponins exhibit notable therapeutic properties, including cholesterol reduction, blood glucose regulation, asthma relief, antioxidant effects, antihypertensive activity, and antimicrobial action. However, their potential cytotoxicity and other adverse effects must also be considered (143).

The rising demand for saponins has driven increased research into both natural and synthetic production methods to meet market and industrial needs. Processing techniques play a critical role in determining the content and bioavailability of saponins, as they influence the structural integrity and interaction between the aglycone core and attached sugar moieties, which in turn affect their functional and therapeutic properties (145). Studies highlight the therapeutic value of specific saponins, such as platycosides from balloon flower (*Platycodon grandiflorus*), which are widely incorporated into dietary supplements and show promising efficacy in respiratory health management (146).

Research has identified *Bacopa monnieri* (Brahmi) as a saponin-rich medicinal plant used in culinary applications. When incorporated into curry preparations, saponin-containing ingredients can help reduce bitterness while enhancing the overall flavor profile, contributing to improved palatability and sensory appeal (147). Another study demonstrated that daucosterol, a bioactive compound isolated from *Eleocharis dulcis* (water chestnut) peels, exhibits anti-hyperglycemic properties, suggesting its potential as a functional dietary supplement (148). The intensified research focus on saponins is primarily driven by their extensively characterized bioactivities and their widespread presence in commonly consumed dietary sources, including tea, cereals, legumes, and medicinal botanicals. Owing to their natural abundance and diverse pharmacological properties, saponins have become the subject of considerable scientific investigation aimed at elucidating their therapeutic potential and underlying biochemical mechanisms (143).

### 5.3 Flavonoids

Flavonoids are diverse polyphenolic plant compounds, categorized into several subclasses, each with distinct dietary sources. For example, flavonols are abundant in foods such as broccoli, onions, tea, and a variety of fruits (149), while flavones are commonly found in chamomile, tea, parsley, and celery (150). Flavanones occur predominantly in citrus fruits (151), whereas flavanols are richly present in apples, cocoa, grapes, tea, and red wine (152). Anthocyanidins are highly concentrated in red wine and berries (153), and isoflavones are primarily derived from soy-based products (154). Their structural variations determine their ability to influence different metabolic pathways (155).

After ingestion, the bioavailability, distribution, and formation of bioactive flavonoid metabolites are determined by differences in absorption, metabolism, administration, and excretion (156, 157). Soy isoflavones, flavonols, and flavones are among the most prevalent dietary flavonoids. Their concentrations in foods vary depending on environmental conditions (e.g., sunlight exposure, ripeness), genetic factors (e.g., plant variety), and postharvest processing techniques (158). As natural phenolic antioxidants, flavonoids play a critical role in human nutrition by mitigating oxidative stress and contributing to disease prevention. Dietary sources such as leafy greens, olives, fruits, red wine, soybean oil, tea, and dark chocolate provide substantial health benefits attributed mainly to their high flavonoid content and associated antioxidant activity (159). Beyond this, some flavonoids exhibit additional biological effects, including antiallergic, antiviral, anti-inflammatory, and anticancer properties, while also influencing metabolic pathways in mammals (160, 161).

Extensive scientific evidence, from both *in vitro* studies and clinical trials, consistently demonstrates that flavonoid-rich foods such as cocoa, tea, and berries exert beneficial effects on cardiovascular health and metabolic function, including improvements in endothelial function, lipid profiles, and insulin sensitivity (162). Notably, cocoa flavonoids have a modest but measurable impact on key physiological markers, including blood pressure, insulin sensitivity, endothelial function, and lipid profiles (157, 163).

These compounds exert anti-inflammatory effects through multiple molecular pathways, including the inhibition of key enzymes such as cyclooxygenase (COX) and lipoxygenase, as well as the suppression of pro-inflammatory transcription factors like nuclear factor-kappa B (NF-κB), thereby reducing the synthesis of inflammatory mediators (164). Furthermore, as powerful antioxidants, flavonoids neutralize free radicals and prevent their generation. They also play a crucial role in regulating immune cells and inflammatory signaling pathways (161, 165). Multiple studies have demonstrated that flavonoids possess potent antioxidant and anti-inflammatory properties, as well as notable anticancer effects mediated through mechanisms such as free radical scavenging, modulation of cell signaling pathways, and induction of apoptosis in malignant cells (164). Furthermore, flavonoids have been shown to exhibit antiviral and antimicrobial activities, which may contribute to their protective effects against infections and indirectly support the prevention of coronary heart disease by reducing systemic inflammation and pathogen-induced vascular damage (164).

Ongoing and future research will undoubtedly advance our understanding of the critical roles flavonoids play in both nutritional health and therapeutic applications, reinforcing their significance as bioactive constituents in functional foods and pharmacological formulations (165). There is a need to develop a suitable model capable of comprehensively analyzing flavonoid extraction, characterization, bioavailability, and administration (165).

### 5.4 Alkaloids

Alkaloids are nitrogen-containing compounds naturally occurring in various plant and animal species (166, 167). Due to their complex structures and potent physiological effects, these compounds warrant thorough investigation for their potential role in reducing uric acid levels. Recent studies suggest that alkaloids can inhibit xanthine oxidase and adenosine deaminase activity while promoting uric acid excretion and suppressing its reabsorption (168).

Alkaloids, a diverse class of nitrogen-containing secondary metabolites, are categorized into various pharmacological groups and exhibit a wide range of bioactivities, including astringent, adrenergic, toxic, antibiotic, diuretic, stimulant, anti-inflammatory, antihypertensive, antimycotic, analgesic, antigout, expectorant, emetic, and antispasmodic effects (168). In addition, dietary alkaloids hold substantial significance across multiple disciplines, including organic chemistry, food technology, nutraceutical innovation, and pharmaceutical development, due to their diverse bioactivities and structural complexity (168).

Medicinal alkaloids, when ingested in large quantities, have been linked to the onset of several diseases, including cancer and cardiovascular issues (169). The degree of dependence fluctuates according to the individual types of alkaloids and their associated concentrations. Alkaloids are broadly classified into six major categories, with each family exhibiting distinct physicochemical and pharmacological properties derived from its unique chemical structure (170, 171).

Alkaloids exhibit beneficial properties for human health; however, certain compounds, such as cocaine, can have severe adverse effects, including dental enamel erosion and caries formation (172). Excessive caffeine intake has been associated with an increased risk of certain cancers and adverse pregnancy outcomes, including spontaneous abortion, according to epidemiological and clinical studies. Due to these potential risks, alkaloid-containing foods are regulated, as these naturally occurring nitrogenous compounds are present in many dietary sources (173).

### 5.5 Vitamins

Vitamins are essential for cellular function, growth, and development (174). They are broadly classified into two group**s**: fat-soluble (A, D, E, and K) and water-soluble (B-complex and C) vitamins (155). Fat-soluble vitamins are stored in the liver, adipose tissue, and skeletal muscles. In contrast, water-soluble vitamins (except vitamin B12) are not retained in the body and are primarily excreted through urine (175).

Maintaining a balanced diet is essential for sustaining adequate vitamin levels. Research has shown that the oral bioavailability of biotin is relatively low in both humans and animals, highlighting the need for optimized dietary intake and, in some cases, supplementation (176, 177). Vitamin E (DL-α-tocopherol), known as tocopherol, occurs naturally in high concentrations in chlorophyll-containing plant tissues and grass seed embryos. While natural sources are abundant, most commercial vitamin E products consist of its synthetic form, the most extensively studied variant of fat-soluble vitamin E (178). Owing to its well-documented health benefits, tocopherol, along with other antioxidant compounds, is widely utilized across various industries, including pharmaceuticals, cosmetics, and food and animal feed production, where it serves both functional and preservative roles (179).

Current research on bioactive compounds in functional foods is limited in scope and depth, underscoring the need for more comprehensive and interdisciplinary studies to elucidate their health-promoting processes and potential applications thoroughly (180). More thorough analysis and characterization of these functional components could substantially advance the development of next-generation functional food products (180).

Recent advances in biotechnology have significantly enhanced the efficiency of extracting and isolating bioactive compounds, thereby accelerating the diversification of functional ingredients available for use in food, pharmaceutical, and nutraceutical applications. For the natural vitamin E industry, three particularly promising research directions have emerged: (1) process optimization for natural vitamin E extraction, (2) methylation approaches for non-α-tocopherol derivatives, and (3) creation of enhanced downstream applications to increase product value (180).

### 5.6 Carotenoids

Carotenoids represent a diverse group of lipid-soluble pigments widely distributed in plants, playing a crucial role in photoprotection (181). These hydrocarbon compounds contain at least 40 carbon atoms with conjugated double bond systems, existing in both oxygenated and non-oxygenated forms. Epidemiological studies have consistently associated carotenoid-rich diets with a decreased risk of various cancers, likely due to the antioxidant, anti-inflammatory, and immune-modulating properties of these compounds (182, 183). Among these compounds, lutein emerges as the predominant polar carotenoid, contrasting with non-polar counterparts such as lycopene, α-carotene, and β-carotene (184, 185).

As a xanthophyll, lutein typically co-occurs with zeaxanthin, with commercial lutein extracts (derived from *Tagetes erecta*) containing approximately 90% lutein and 5% zeaxanthin. Fruit and vegetable carotenoid concentrations vary significantly depending on storage conditions and ripening stage (186). Notably, lycopene exhibits vigorous chemopreventive activity, primarily through its ability to scavenge reactive oxygen species (ROS), thereby mitigating oxidative stress and reducing DNA damage associated with carcinogenesis.

The global carotenoid market encompasses numerous variants, including lutein, β-carotene, astaxanthin, and lycopene (187). While most staple crops naturally contain limited carotenoid concentrations (186), biotechnological advances have significantly enhanced carotenoid levels in food crops (188). These improvements have been achieved by strategically manipulating carotenoid biosynthetic pathways, targeted gene expression modifications, and microbial fermentation techniques (189).

### 5.7 Fatty acids

Fatty acids constitute an essential category of lipid molecules found throughout biological systems, where they serve crucial functions in numerous physiological processes (190). Structurally, they are categorized into two primary types: saturated fatty acids (SFAs) and unsaturated fatty acids—the latter comprising both monounsaturated (MUFAs) and polyunsaturated (PUFAs) subclasses (191, 192).

SFAs consist of straight hydrocarbon chains without double bonds, typically ranging from 14 to 24 carbon atoms in length. In contrast, PUFAs have shorter chains (16–22 carbons) and contain 2–6 double bonds (193). A distinct subgroup, highly unsaturated fatty acids (HUFAs), is defined by longer chains (≥20 carbons) with three or more double bonds (194, 195). Fatty acids are further classified as medium-chain (MCFA) or long-chain (LCFA) based on their carbon length.

Research by Ramírez et al. (196) indicates that MCFAs are absorbed more efficiently across the intestinal mucosa than LCFAs, due to their shorter carbon chain length and greater solubility. In contrast to saturated fats, unsaturated fats remain liquid at room temperature and are associated with a range of health benefits, including improved lipid profiles and reduced cardiovascular risk (196).

MUFAs are found in olive oil, avocados, nuts (pecans and almonds), peanut oil, canola oil, and pumpkin seeds (197). PUFAs are abundantly found in dietary sources such as sunflower oil, corn, flaxseeds, walnuts, and seafood. Ongoing exploration of emerging technologies, underlying biological mechanisms, and novel applications may further enhance scientific understanding and optimize the health-promoting potential of these essential fatty acids (198).

### 5.8 Polyphenolic components

Phenolic compounds are widely recognized as potent natural antioxidants (199, 200); however, their bioavailability is highly influenced by structural characteristics, such as molecular weight, glycosylation, and degree of polymerization, as well as the complexity of the food matrix and interactions with other dietary components. Phenolic compounds are widely recognized as potent natural antioxidants (152). However, their bioavailability is highly influenced by structural characteristics, such as molecular weight, glycosylation, and degree of polymerization, as well as the complexity of the food matrix and interactions with other dietary components (152).

In plants, leaves function as the primary interface for defense against ultraviolet radiation and pathogenic invasion, while simultaneously playing essential roles in photosynthesis, growth regulation, reproductive development, and pigmentation (201, 202). The antioxidant capacity of phenolic compounds is primarily determined by their molecular structure, particularly the presence of a benzene ring and the number and positioning of hydroxyl (OH) groups. The benzene ring enhances stability by enabling interactions with free radicals (203). A notable example is gallic acid, a phenolic acid featuring three hydroxyl groups and one carboxylic acid group (204). These hydroxyl groups allow gallic acid to act as an antioxidant by generating free radicals that counteract oxidative damage (205).

Plant-derived phenolic extracts have gained considerable attention as effective natural alternatives to synthetic antioxidants for inhibiting lipid oxidation in food systems, thereby enhancing shelf life and preserving nutritional and sensory qualities (206). Research indicates that phenolic compounds obtained from various botanical sources, including agricultural byproducts like peels, stems, and seeds, often perform comparably or surpass traditional antioxidants such as ascorbic acid and tocopherols (201, 207). Emerging evidence indicates that purified phenolic compounds effectively mitigate oxidative degradation and color deterioration in bulk oils, meat products, and lipid-based emulsions by scavenging free radicals and chelating pro-oxidant metal ions. Moreover, these plant-based extracts show promising applications as functional dietary antioxidants (208, 209).

Phenolic compounds represent a significant group of antioxidants acting as free radical scavengers. These compounds effectively suppress lipid oxidation by preventing the initiation phase or interrupting the propagation phase of oxidative chain reactions. Through this mechanism, they minimize the formation of volatile degradation products, particularly ketones and aldehydes, that contribute to food rancidity (210, 211). Nevertheless, as the commercial application of polyphenol-based nanoparticles continues to expand, comprehensive safety assessments must be prioritized throughout their development. Regulatory authorities should establish and enforce standardized evaluation protocols to ensure rigorous safety validation before approving products for consumer use (211).

### 5.9 Essential oils

Essential oils are complex mixtures of volatile, low-molecular-weight compounds, primarily composed of monoterpenes and sesquiterpenes (212). However, they may also contain important non-terpenoid components such as phenylpropanoids and sulfur- and nitrogen-containing compounds (213, 214). These oils play vital roles in plant ecophysiology, contributing to defense mechanisms, environmental adaptation, and pollination. Furthermore, significant advancements have been made in harnessing these compounds for various practical applications (213).

The food industry has effectively incorporated various essential oils approved as safe for human consumption (215). These oils hold Generally Recognized as Safe (GRAS) status from the U.S. Food and Drug Administration (FDA), while the European Commission has similarly authorized specific essential oil components as approved flavoring agents (216, 217). In addition to their flavoring properties, essential oils contribute substantially to food preservation. Their antimicrobial and antioxidant characteristics enable diverse applications, including active food packaging systems that suppress microbial growth and prolong product shelf life (218). Despite existing implementation challenges, essential oils contribute to sustainable food production and align with clean-label initiatives, positioning them as increasingly valuable functional ingredients in contemporary food processing (219).

### 5.10 Phytosterols

Plant sterols (phytosterols and stanols) are bioactive plant compounds recognized for their cholesterol-lowering properties in humans (220). Due to their structural similarity to cholesterol, phytosterols competitively inhibit its absorption in the intestinal lumen, enhancing fecal excretion and subsequently lowering circulating plasma cholesterol levels (221, 222).

While naturally present in unrefined vegetable oils (e.g., olive, sesame, and nut oils), nuts (such as pistachios and macadamias), herbs (such as thyme, oregano, and sage), and other plant foods (223), their endogenous concentrations typically exert limited physiological effects. However, when concentrated and incorporated into functional foods, such as fortified spreads, dairy products, or dressings, phytosterols demonstrate clinically meaningful efficacy in cholesterol management (224).

### 5.11 Cannabinoids

Cannabis is a comprehensive category representing an annual herbaceous plant belonging to the Cannabaceae family (225). The primary species recognized within the genus *Cannabis* include *Cannabis sativa, Cannabis indica*, and, though still subject to taxonomic debate, *Cannabis ruderalis* (226). *C. sativa* produces a diverse array of non-nutritive phytocannabinoids, bioactive compounds that include the well-characterized delta-9-tetrahydrocannabinol (THC) and cannabidiol (CBD) as its most prominent representatives (227). To date, researchers have identified approximately 110 distinct cannabinoids within the *Cannabis* species, each exhibiting unique chemical structures and pharmacological profiles. These specialized metabolites are predominantly biosynthesized and stored in glandular trichomes–secretory structures found on flowering plants, liverworts, and certain fungi (228).

Hemp plants contain numerous non-psychoactive cannabinoids, such as cannabichromene, cannabigerol, and cannabidiol (CBD), as well as a wide range of non-cannabinoid constituents belonging to various classes of naturally occurring phytochemicals, including terpenes, flavonoids, and phenolic compounds (229). Emerging research suggests that specific cannabinoids demonstrate therapeutic potential for managing diverse medical conditions, particularly chronic pain, anxiety disorders, and cachexia (228). These compounds may also serve as appetite stimulants and possess anti-nausea effects (230). In low-THC hemp varieties, fundamental metabolic pathways generate primary metabolites (amino acids, fatty acids, and steroids) that serve as precursors for secondary metabolites. These include terpenoids, flavonoids, alkaloids, lignans, and the distinctive C21 terpenophenolic compounds known as phytocannabinoids (231).

The psychoactive THC originates from the decarboxylation of its acidic precursor THCA, while non-psychoactive CBD forms through analogous decarboxylation of CBDA (227). The contemporary market offers a wide array of products containing cannabis extracts; however, this growing availability also heightens the potential for adverse effects among consumers. A significant proportion of cannabis-infused edibles lack adequate regulatory oversight, posing a notable risk of accidental ingestion, particularly among vulnerable populations such as children (232).

The incidence of such cases has increased in regions where cannabis has been legalized or decriminalized. While most cannabis-infused edibles have not yet received FDA approval, ongoing research is assessing their long-term safety and potential cumulative health effects (233). Updating cannabis regulations requires the implementation of stringent safety measures to mitigate the risk of pediatric cannabis toxicity and prevent unintentional overdoses, particularly in the context of edible and easily accessible cannabis-infused products. Additionally, understanding industry standards and consumer practices—particularly concerning the proper preparation and packaging of cannabis edibles—is crucial to ensuring both safety and an optimal consumption experience (234).

## 6 Traditional and modern extraction techniques of bioactive compounds from plants

The solubility of active compounds is influenced by other solutes, various molecules in the plant matrix, and the solvent employed for solubilization, all of which affect extraction (235). Prior to extraction, plant tissue must be thoroughly homogenized to disrupt cellular structures and enhance the efficiency and yield of bioactive compound recovery (236).

Bioactive natural chemicals are consistently present in plant matrices and are often found in limited quantities in natural sources (237, 238). All components of the plant, including leaves, roots, barks, tubers, wood, gums or oleoresins, exudates, fruits, figs, flowers, rhizomes, berries, and twigs, produce active chemicals in varying quantities and concentrations. To maximize tissue extract yield, selecting the optimal extraction procedure is crucial (239).

The extraction efficiency depends on several key factors, including processing methods, plant matrix properties, solvent selection, and operational parameters, such as temperature, pressure, and duration. As a critical step in herbal product manufacturing, the extraction process profoundly influences both the qualitative composition and quantitative yield of bioactive compounds (240).

Given the vast taxonomic diversity of plant species and the complexity of their phytochemical profiles, a systematic and high-throughput screening approach is essential for the efficient identification and evaluation of bioactive constituents (240). Following efficient extraction, downstream processes such as separation, identification, and structural characterization of bioactive compounds can be carried out systematically and effectively. Multiple variables affect bioactive compound recovery, with critical considerations including solvent choice, starting material quality, and the selection of extraction techniques (241).

Efficiently isolating bioactive compounds from natural sources necessitates strategically implementing optimized extraction methodologies. Contemporary research has increasingly focused on the extraction, characterization, and application of phenolic compounds from plant matrices due to their significant bioactive potential (242). To maximize the recovery of these valuable phytochemicals, an integrated extraction strategy combining multiple complementary techniques has proven more effective than relying on a single method, as it enhances yield, selectivity, and preservation of compound integrity (242).

Recent decades have witnessed significant advancements in extraction technologies, marked by improved environmental sustainability, reduced reliance on synthetic chemicals, shorter processing times, and enhanced extract quality (243). These modern extraction techniques are gaining prominence for improving both the yield and selectivity of bioactive plant compounds (36). Environmentally conscious methods that minimize energy consumption and organic solvent use have been formally recognized as “green technologies” (244). The food industry has enhanced its processing capabilities through advanced extraction technologies, particularly ultrasound-assisted, pulsed electric field, enzymatic, microwave, supercritical fluid, and pressurized liquid extraction (PLE) systems (245).

Researchers have proposed innovative extraction strategies to overcome the constraints of conventional extraction approaches. The food industry has demonstrated growing interest in advanced extraction technologies such as pressurized liquid extraction (PLE), UAE, MAE, subcritical water extraction (SWE), supercritical fluid extraction (SFE), enzyme-assisted extraction (EAE), and PEFE, due to their enhanced efficiency, reduced solvent usage, and ability to preserve thermolabile bioactive compounds (246). Academic research indicates that integrating modern extraction techniques provides a highly effective strategy for achieving both rapid processing and enhanced extraction efficiency, while preserving the structural integrity and bioactivity of target phytochemicals (246). A substantial body of evidence demonstrates that advanced extraction technologies markedly enhance process efficiency and improve the quality, purity, and stability of extracted bioactive compounds (246).

**Figure 6:**
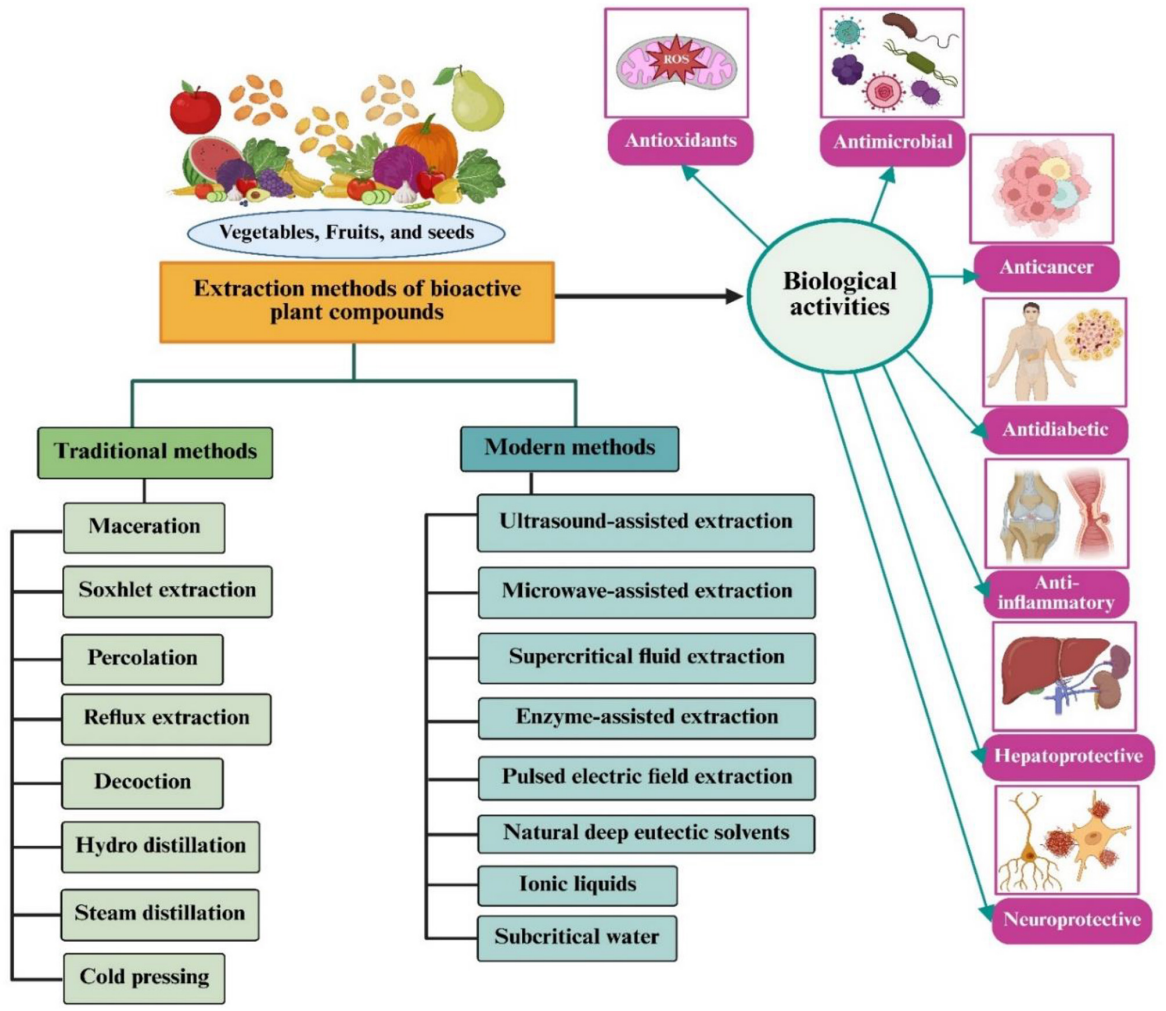
Various extraction techniques (both conventional and innovative) and the biological properties of the extracted bioactive substances.

Table 2 presents a comparative study of conventional and current extraction processes, including their advantages, disadvantages, and limitations. Figure 6 depicts the various extraction methods (both traditional and innovative) and the biological activity of the extracted bioactive compounds.

**Table 2:** Comparative assessment of the effectiveness, limitations, and applications of conventional and novel extraction techniques.

**Extraction method**	**Category**	**Advantages**	**Disadvantages**	**Limitations**	**References**
Maceration	Traditional	Minimal equipment requirements; cost-effective implementation; ambient temperature operation; thermolabile compound preservation; large-scale processing capacity	Extended processing time (48–168 h); substantial solvent volume requirements; low extraction efficiency; microbial contamination susceptibility	Mass transfer inefficiency; extended processing duration; solvent selectivity constraints; time-intensive methodology	(370, 373)
Soxhlet extraction	Traditional	Complete analyte recovery; high extraction efficiency for lipophilic compounds; solvent regeneration capability; continuous extraction process; high purity yields	High energy consumption; extended extraction duration; thermal degradation risk; large solvent volumes; environmental impact concerns	Thermal degradation susceptibility; environmental impact; energy-intensive operation; solvent disposal requirements	(353, 647)
Percolation	Traditional	Enhanced mass transfer dynamics; superior efficiency compared to static maceration; continuous solvent renewal; selective compound extraction	Operational complexity; elevated capital investment; skilled operator requirement; increased solvent consumption; equipment complexity	Operational complexity; higher capital requirements; specialized skill demands; process control requirements	(353, 373)
Reflux extraction	Traditional	Temperature-controlled extraction; improved solubility kinetics; enhanced diffusion rates; solvent conservation; reduced processing time	Energy-intensive operation; thermal treatment requirement; potential compound degradation; complex apparatus setup; solvent vapor losses	Thermal stability requirements; energy demands; solvent vapor losses; needs equipment maintenance	(353, 648)
Decoction	Traditional	Aqueous-based methodology; elimination of organic solvents; traditional pharmaceutical applications; cost-effective processing; environmentally friendly	Limited to hydrophilic compounds; thermal degradation susceptibility; extended boiling time; low efficiency for lipophilic compounds	Compound solubility dependency; thermal stability constraints; limited extraction scope; pH sensitivity	(370, 373)
Hydrodistillation	Traditional	Selective volatile compound recovery; water-based extraction medium; established methodology; simple apparatus configuration	Volatile compound limitation; extended processing time; thermal degradation risk; low efficiency; water co-distillation interference	Volatility requirements; steam distillation co-extraction; time-consuming process; energy-intensive operation	(353, 649)
Steam distillation	Traditional	Reduced thermal stress compared to hydrodistillation; enhanced oil quality preservation; efficient volatile compound isolation	Thermal treatment requirement; limited compound spectrum; potential oil composition alteration; equipment complexity	Thermal sensitivity; compound volatility dependency; oil quality variations; process control requirements	(353, 649)
Cold pressing	Traditional	Solvent-free methodology; mechanical extraction process; preservation of compound integrity; high-quality oil production	Material-specific limitations; mechanical stress application; lower yield potential; physical extraction constraints	Material suitability constraints; mechanical limitations; yield restrictions; quality variations	(370, 373)
Infusion	Traditional	Mild extraction conditions; aqueous extraction medium; minimal thermal degradation; thermosensitive compound preservation	Mild extraction efficiency; low compound recovery; hydrophilic compound limitation; extended contact time requirement	Extraction efficiency limitations; time requirements; solubility constraints; concentration challenges	(370, 373)
Ultrasound-assisted extraction (UAE)	Modern	Accelerated extraction kinetics; reduced solvent consumption; enhanced mass transfer; lower operating temperatures; environmentally sustainable technology	High capital investment; potential compound degradation; heat generation effects; scale-up challenges; parameter optimization complexity	Equipment dependency; process optimization complexity; potential structural modifications; scale-up challenges	(648, 650)
Microwave-assisted extraction (MAE)	Modern	Rapid heating mechanism; improved extraction yields; minimal solvent requirements; thermolabile compound preservation; high processing efficiency	Substantial equipment costs; non-uniform heating potential; limited industrial scalability; reduced efficiency for non-polar compounds	Heating uniformity challenges; temperature control requirements; polar solvent dependency; limited scalability	(651–653)
Supercritical fluid extraction (SFE)	Modern	Non-toxic residue elimination; high selectivity parameters; compound integrity preservation; environmental safety; high-purity extract production	Extremely high capital investment; limited solvent options; high-pressure expertise requirement; energy-intensive operation	Modifier requirements; pressure system complexity; economic viability constraints	(353, 354)
Pressurized liquid extraction (PLE)	Modern	Accelerated extraction process; high efficiency parameters; automated operation; broad compound applicability; reduced solvent usage	High equipment investment; temperature limitations; Specialized training requirements; high maintenance costs	Temperature constraints; pressure system requirements; solvent recovery necessities; operating cost considerations	(353, 354)
Enzyme-assisted extraction (EAE)	Modern	Mild operating conditions; high substrate specificity; bioactivity preservation; environmentally sustainable; enhanced selectivity	Elevated enzyme costs; multiple parameter optimization; limited enzyme commercial availability; environmental sensitivity factors	Enzyme stability requirements; pH and temperature sensitivity; substrate specificity; commercial scale limitations	(353, 354)
Pulsed electric field extraction (PEFE)	Modern	Non-thermal processing; cell membrane permeabilization; rapid treatment times; energy-efficient operation; enhanced extraction yields	High initial capital investment; complex parameter optimization; limited industrial-scale equipment; safety protocol requirements	Equipment availability constraints; parameter interdependency; industrial scale limitations; safety protocol requirements	(371, 372)
Natural deep eutectic solvents extraction (NDESE)	Modern	Biodegradable solvent system; non-toxic formulation; tunable physicochemical properties; high extraction efficiency; sustainable alternative	Limited commercial availability; viscosity-related challenges; water content sensitivity; scale-up difficulties	Preparation complexity; physical property limitations; commercial availability constraints; process standardization challenges	(365, 654)
Ionic liquid extraction (ILE)	Modern	High selectivity; recyclable solvent system; low volatility; thermal stability; tunable physicochemical properties	High synthesis costs; potential toxicity concerns; limited commercial-scale availability; complex recovery processes	Synthesis complexity; purification requirements; cost considerations; regulatory approval necessities	(368, 369)
Subcritical water extraction (SWE)	Modern	Water as primary solvent; high selectivity for polar compounds; elimination of organic solvents; environmentally sustainable; effective polar compound extraction	High temperature requirements; pressure equipment necessity; energy-intensive operation; limited thermostable compound selectivity	Energy requirements; equipment complexity; process control demands; compound stability limitations	(353, 354)
Hydrothermal extraction	Modern	Elevated temperature and pressure operation; enhanced mass transfer kinetics; water-based extraction; thermostable compound suitability; high yield potential	High energy consumption; pressure equipment requirements; temperature control complexity; limited compound selectivity	Process control requirements; energy-intensive operation; equipment complexity; compound selectivity limitations	(353, 354)

### 6.1 Conventional extraction techniques

Conventional extraction techniques remain the gold standard for isolating bioactive compounds from solid food matrices (Tables 1, 2). The most widely employed methods are Soxhlet extraction, heated reflux extraction, and maceration. Soxhlet extraction carries special historical importance, initially developed in 1879 by German chemist Franz Ritter von Soxhlet for lipid extraction (247). The Soxhlet method remains a crucial benchmark for evaluating the performance of modern extraction technologies. In their study, Kodal and Aksu (248) utilized Soxhlet extraction to isolate carotenoid pigments from orange peel. Their results demonstrated optimal carotenoid recovery (4.5 mg carotene/g dry peel) when processing frozen peel material at 79°C using ethanol with a 40:1 liquid-to-solid ratio. However, the researchers noted that the extracted compounds were susceptible to lipid oxidation degradation, resulting in the breakdown into terpene monomers (248).

Similarly, Caldas et al. (249) successfully isolated phenolic compounds—particularly catechin, rutin, and epicatechin—from grape peel using Soxhlet extraction. The heat reflux extraction method, which employs specialized apparatus, substantially enhances extraction efficiency by improving mass transfer between the solvent and solute at elevated temperatures (249). This technique operates by continuously cycling heated solvent vapors through the sample matrix under tightly regulated condensation conditions, thereby facilitating efficient solubilization and extraction of target compounds (250).

Although these extraction techniques are cost-effective and simple, they may degrade thermally unstable compounds. After reducing the solid sample size, maceration is often preferred for heat-sensitive components (251). For instance, methanol maceration at 25°C yielded the highest anthocyanin concentration (300 mg/g) from grape skins. Similarly, Sultana et al. (252) found that methanol maceration was the most effective method for extracting flavonoids from citrus peels, resulting in high yields and purity. Additionally, catechin was extracted from *Arbutus unedo* fruits using maceration with 3.7% diluted ethanol at 79.6°C (253). While maceration typically involves prolonged extraction at room temperature, Soxhlet and heat reflux methods can complete extraction in just a few hours at 90°C (254).

While conventional extraction techniques offer advantages such as operational simplicity, cost efficiency, and proven effectiveness in isolating bioactive compounds, they present several notable drawbacks. Key limitations of this technique include extended processing times, high consumption of potentially hazardous organic solvents, and increased susceptibility of bioactive compounds to degradation due to environmental factors such as oxygen exposure, photolability, and thermal instability.

### 6.2 Novel extraction techniques

Contemporary extraction technologies have emerged to overcome the limitations of conventional methods, offering enhanced efficiency and improved yields of bioactive compounds, as shown in Table 1 and Figure 1. Modern extraction technologies include several advanced methods designed to improve efficiency and selectivity (246). These include as follows: UAE which uses sound waves to break plant cell walls; MAE which heats samples quickly using electromagnetic radiation; infrared-assisted extraction (IRAE), which applies focused thermal energy; PEFE which uses short electrical pulses to increase cell permeability; PLE which operates at high temperatures and pressures to enhance solvent penetration; SFE which uses carbon dioxide in a supercritical state as a solvent; SWE, which alters the properties of water under moderate conditions to improve extraction; and EAE, which employs specific enzymes to break down plant cell structures and release bioactive compounds (246). Each technique offers distinct advantages in terms of extraction efficiency and selectivity, while effectively addressing the inherent limitations associated with conventional methods (Table 2).

#### 6.2.1 SFE

SFE is recognized as an innovative and environmentally sustainable extraction method (255). The supercritical state was first discovered in 1822 by French physicist Baron Charles Cagniard de la Tour, who identified unique alterations in solvent behavior at critical pressure and temperature thresholds (256). Later, in 1869, Thomas Andrews introduced the term “critical point” while studying the effects of pressure and temperature on carbon dioxide in a sealed glass tube. He defined this as the threshold at which the phase equilibrium curve terminates, marked by critical pressure (Pc) and temperature (Tc), beyond which the liquid and gas phases become indistinguishable (256).

Hannay and Hogarth (257) developed the principles of SFE and demonstrated that the supercritical properties of carbon dioxide (CO_2_) could be successfully harnessed, marking a significant advancement in this technology. The first commercial application of supercritical fluid technology was developed in Germany for the decaffeination of green coffee beans using supercritical CO_2_. Subsequently, Australia became a pioneer in employing liquid CO_2_ for the extraction of hop oils in the brewing industry (258). By the 1980s, both techniques had been optimized and widely adopted for industrial applications across multiple countries (258).

SFE technology is currently used to manufacture various popular products across multiple industries, including chemicals, food, pharmaceuticals, and fuels (259). One of the key advantages of SFE is its ability to leave no harmful residues in the final product, making it particularly effective for extraction processes (259). These processes are primarily used for: (1) isolating beneficial bioactive compounds such as pigments, flavors, and other biomolecules, or (2) removing undesirable contaminants like pesticides, toxins, and organic pollutants (260, 261).

Extraction efficiency can be enhanced by incorporating a cellulose matrix into the solid substrate, which remains chemically inert to both the solvent and solute, while facilitating improved mass transfer and increasing overall extraction yields. SFE operates through two distinct phases: first, the supercritical solvent solubilizes target compounds from the solid matrix, followed by their subsequent separation from the solvent during controlled depressurization (262, 263).

CO_2_ has emerged as the preferred supercritical solvent, owing to its favorable physicochemical properties, particularly its relatively low critical temperature (31°C) and moderate critical pressure (74 bar). These characteristics permit effective operation within practical pressure ranges (generally 100–450 bar) while maintaining process efficiency (264, 265). However, a limitation of CO_2_ is its low polarity, which makes it ideal for extracting non-polar compounds (e.g., fats and lipids) but less effective for polar substances like many pharmaceuticals (266, 267).

To overcome this, chemical modifiers can be added to increase CO_2_'s polarity (266–268). The addition of minimal solvent modifiers, such as little as 0.5 ml of dichloromethane (CH_2_Cl_2_), can significantly enhance extraction efficiency, yielding results comparable to those obtained through conventional 4-h hydrodistillation procedures (238, 269). SFE efficiency depends on carefully optimizing multiple operational parameters, which play a pivotal role in successfully isolating bioactive phytochemicals from plant matrices (238). Precise regulation of these critical variables is essential for achieving optimal extraction yields while maintaining process effectiveness (238).

Optimizing extraction parameters is crucial for achieving optimal results with SFE. Seven critical factors govern process efficiency as follows: (1) temperature, (2) pressure, (3) feedstock moisture content, (4) particle size distribution, (5) extraction duration, (6) CO_2_ flow rate, and (7) solvent-to-feed ratio (270). SFE's superiority over conventional extraction methods stems from the unique properties of supercritical fluids, including tunable density, improved mass transfer characteristics (characterized by low viscosity and high diffusivity), and adjustable solvation power (271, 272).

These enhanced transport properties (notably 10–100 times greater diffusivity than liquids) facilitate deeper penetration into solid matrices and accelerated extraction kinetics (273). The method's hallmark characteristic is pressure-dependent density modulation, which enables precise control over solvent strength through manipulation of solubility. Compared to traditional techniques, SFE offers the following four distinct advantages: (1) use of non-toxic, GRAS-certified solvents, (2) increased extraction yields, (3) reduced processing times, and (4) direct compatibility with analytical instrumentation, including gas chromatography and SFC systems (255, 274).

#### 6.2.2 SWE

SWE has gained recognition as an eco-friendly alternative for isolating bioactive compounds from plant and biological matrices (275). This method is particularly attractive for industrial-scale applications due to its high extraction efficiency, cost-effectiveness, operational safety, low energy requirements, and minimal environmental impact (276). These combined advantages—enhanced process efficiency and reduced environmental impact—have fueled growing research interest in SWE technology. The technique has proven effective for recovering valuable bioactive components, including proteins, polysaccharides, polyphenols, and antioxidants (277–279). A unique characteristic of SWE is its ability to modify the molecular structure of extracted compounds, potentially enhancing their biological activity (280).

SWE is an efficient and environmentally sustainable technology widely applicable across various extraction industries (281). SWE differs from conventional extraction methods by utilizing the altered physicochemical properties of water when maintained in its subcritical state, at temperatures between 100 and 374°C and pressures exceeding 22.1 MPa, thereby enhancing its solvating power for a wide range of polar and moderately non-polar compounds. This innovative approach provides an environmentally friendly, economically viable, and inherently safe extraction platform that eliminates the need for organic solvents (282). Water remains liquid at subcritical conditions (100-374°C) due to applied pressure, while its physicochemical properties undergo significant changes. As temperature increases, diffusion improves, while the dielectric constant, viscosity, and surface tension decrease (283).

Additionally, SWE promotes effective mass transfer through convection and diffusion (284). The SWE process proceeds through a series of interconnected stages that collectively enhance extraction efficiency. Initially, as temperature and pressure increase, solutes are desorbed from active binding sites within the plant matrix. These solutes are then solubilized and dispersed throughout the sample. Subsequently, based on their physicochemical properties and interactions with the matrix, the solutes partition into the subcritical water phase (284).

Finally, the extracted compounds are eluted from the extraction cell and collected using chromatographic or other separation techniques to ensure purity and analytical recovery (285–287). SWE method offers a highly tunable and efficient approach to extraction, making it a promising alternative to traditional techniques (286, 287).

#### 6.2.3 UAE

This technique is a versatile energy source with widespread applications in manufacturing, medicine, and navigation (288). Ultrasound consists of sound waves at frequencies beyond human hearing (>20 kHz) and is utilized in industrial processes such as cleaning, degassing, emulsification, extraction, crystallization, and homogenization (289, 290). The UAE offers several advantages, including rapid processing, high selectivity, reproducibility, compatibility with thermolabile compounds, and superior energy efficiency, positioning it as an environmentally sustainable technology consistent with the principles of green chemistry and engineering (291). In recent years, the UAE has become an efficient method for extracting bioactive compounds from natural sources such as fruits, vegetables, algae, and fungi (292).

Unlike conventional extraction techniques, which often require prolonged heating and stirring (taking hours or even days), UAE can achieve comparable or superior results in minutes to a few hours (293). UAE is an advanced technology that outperforms conventional methods due to its efficiency and effectiveness. The mechanism involves ultrasonic waves disrupting cell walls, enhancing solvent penetration, and improving extraction efficiency. This method significantly reduces extraction time, solvent consumption, and energy usage (293). Additionally, the UAE aligns with green chemistry principles by facilitating the replacement of hazardous organic solvents with safer, GRAS alternatives, such as water–ethanol mixtures (294, 295). This shift enhances sustainability and safety in extraction processes while maintaining high yields (295, 296).

UAE offers numerous benefits, including faster processing times, simplified procedures, lower operational temperatures, reduced solvent and energy consumption, and higher extraction yields. This technique enhances mass transfer and leverages the cavitation phenomenon to improve extraction efficiency (297, 298). Several parameters influence the UAE process, such as frequency, sonication power, extraction duration, and ultrasonic wave distribution (299, 300). UAE has demonstrated high efficacy in isolating bioactive compounds from medicinal plants, owing to its ability to enhance mass transfer and disrupt cellular structures through acoustic cavitation (301).

Its key advantages include shorter extraction times, lower energy and solvent requirements, and improved precision. Additionally, the UAE enables faster energy transfer, better mixing, minimized thermal gradients, selective extraction, compact equipment design, quicker process adjustments, rapid startup, higher yields, and reduced unnecessary processing steps (302–304).

#### 6.2.4 MAE

MAE has gained considerable attention as an efficient extraction method due to its minimal solvent requirements, shorter processing times, high reproducibility, improved recovery yields, enhanced selectivity, and reduced sample manipulation (305, 306). Initially introduced in 1986 for chemical synthesis, microwave energy was later adapted for extracting biological samples to analyze organic compounds. Today, MAE is widely applied across various sample types, including biological, environmental, and geological matrices (305).

In recent years, MAE has become increasingly prominent in research and development for extracting bioactive compounds from plant materials (242). MAE enables faster solute recovery compared to conventional techniques while maintaining high extraction efficiency. As a modern and sustainable method, MAE offers several key advantages, including the efficient extraction of thermolabile compounds, significantly reduced processing times, lower solvent usage, and enhanced isolation of bioactive constituents from plant matrices. These benefits position MAE as a valuable tool in green extraction protocols for natural product research and functional food development (307, 308).

Microwave radiation, a non-ionizing electromagnetic energy, spans frequencies from 300 MHz to 300 GHz (309). Two dominant frequency bands are employed in extraction applications: the 2,450 MHz band (standard in domestic microwaves and laboratory systems) and the 915 MHz band (favored in industrial-scale operations for its enhanced material penetration capabilities) (310, 311). In MAE, solvent selection plays a critical role in determining process efficiency, primarily through two key dielectric properties: the dielectric constant, which reflects the solvent's ability to be polarized in an electric field, and the dielectric loss factor, which indicates its capacity to absorb and convert microwave energy into heat. Together, these properties govern the solvent's microwave coupling efficiency and directly impact extraction performance (312).

Strategic solvent blending offers significant advantages in MAE by modulating dielectric properties to enhance the selectivity of the target compound. The use of low-dielectric solvents serves a dual purpose: (1) maintaining reduced temperatures to safeguard thermolabile components from degradation (313), while (2) creating a thermal gradient where the plant matrix preferentially absorbs microwave energy. This differential heating mechanism facilitates the rapid rupture of plant cell structures, promoting the efficient release and transfer of bioactive constituents into the cooler surrounding solvent phase (314, 315). Elevated temperatures in MAE enhance extraction efficiency by increasing molecular mobility and solubility, strengthening solvent-solute interactions, and generating intracellular pressure that disrupts cell walls, thereby facilitating the release of target compounds (293).

Reducing solvent viscosity improves solvent penetration and solute dissolution (316, 317). Additionally, as temperature rises, the solvent's viscosity decreases, improving its fluidity and dissolution capacity, thereby boosting extraction efficiency (318). Sample preparation involves homogenization, grinding, and milling for optimal solvent-cell matrix interaction. This approach is particularly effective in MAE of flavonoids, where MAE has demonstrated comparable or superior efficiency relative to conventional solvent-based techniques, often achieving higher yields in shorter extraction times (318). Extraction efficiency can be substantially improved by integrating advanced technology and refining process parameters (319).

#### 6.2.5 PEFE

PEFE has gained recognition as an innovative and cost-effective processing technology for food and pharmaceutical applications (320). Initially developed for non-thermal microbial and enzymatic inactivation using short bursts of high-voltage electric pulses, this technique preserves product quality by minimizing thermal degradation, making it particularly suitable for heat-sensitive compounds (321). The first application of PEFE was demonstrated by Ganeva and Galutzov (322), who found that pretreating beer yeast with an electric field of 2.75 kV/cm before maceration significantly increased protein extraction yields.

Subsequent research has confirmed that PEFE treatment increases cell membrane permeability by inducing electroporation, thereby enhancing mass transfer and generating significant scientific interest in its application for the extraction of bioactive compounds (33). Numerous studies have since explored PEFE's potential, particularly for extracting bioactive compounds (323). PEFE technology has emerged as an efficient and gentle alternative to traditional cell disruption methods (324, 325).

PEFE involves the application of repetitive, short-duration electrical pulses, typically in the microsecond to millisecond range, at moderate field strengths (0.5–10 kV/cm) and low specific energy inputs (1–10 kJ/kg), targeting plant tissues to induce electroporation and facilitate the release of compounds (326). The treatment selectively increases membrane permeability while maintaining cell wall structure, stimulating the release of intracellular contents without thermal degradation (326, 327). The non-thermal nature of PEFE offers distinct advantages for extraction processes. When combined with mechanical pressing, PEFE pretreatment significantly improves both yield and quality of fruit and vegetable juices, including those from apples, grapes, and carrots (328, 329).

PEFE technology improves extraction efficiency by significantly reducing processing time, minimizing solvent usage, and operating at lower temperatures, thereby preserving thermolabile compounds and supporting environmentally sustainable practices (330). Additionally, this method enhances the extraction yields of high-value bioactive compounds, particularly polyphenols and natural pigments such as anthocyanins, carotenoids, and betaines, which can be efficiently recovered from both raw plant materials and agri-food processing byproducts (330–332).

#### 6.2.6 PLE

PLE is an environmentally sustainable method for obtaining nutraceuticals from food and herbal sources (333). In contrast to conventional extraction methods conducted at ambient conditions, PLE utilizes solvents at elevated temperatures and pressures, thereby enhancing solvent penetration, solute solubility, and mass transfer rates (334). This approach enhances extraction efficiency by exploiting increased solute solubility and accelerated mass transfer rates that occur when solvents are heated above their atmospheric boiling points under pressurized conditions (334–336).

The PLE technique was commercialized in 1995 by Dionex Corporation under the trade name Accelerated Solvent Extraction (ASE^®^). This extraction method is alternatively referred to as pressurized, accelerated, or enhanced solvent extraction in scientific literature (337).

When water is employed as the extraction medium, the process is designated explicitly as either superheated, subcritical, or pressurized hot water extraction (338, 339). PLE offers a greener alternative by reducing solvent consumption while increasing extraction speed. Its adjustable parameters allow for the selective targeting of specific bioactive compounds (340, 341). This method is especially advantageous when employing water or ethanol as solvents, both of which are classified as GRAS, aligning with green chemistry principles and enhancing their suitability for food and pharmaceutical applications (342, 343).

PLE has been successfully applied to extract thermally labile phytochemicals from various plant materials (344). The process operates in a controlled, inert environment, where solvents remain in a subcritical liquid state despite exposure to temperatures well above their boiling points, facilitated by elevated pressures (345). The combined application of high pressure and elevated temperature enhances overall extraction efficiency by maintaining solvent stability, increasing solute solubility, and accelerating desorption kinetics from the plant matrix (346, 347).

#### 6.2.7 IRAE

Infrared radiation encompasses three spectral regions: near (0.78–3 μm), mid (3–50 μm), and far-infrared (50–1,000 μm). Notably, the penetration depth of infrared radiation is inversely proportional to its energy level (348). When applied to plant matrices, infrared radiation induces atomic and molecular vibrations, which are subsequently converted into thermal energy, facilitating the disruption of cell structures and enhancing the release of target compounds (348). This temperature increase promotes solvent evaporation and disrupts the plant matrix structure, thereby facilitating the liberation of target compounds (348). The far-infrared extraction technique provides unique benefits due to the strong absorption of water and organic compounds at wavelengths exceeding 2.5 μm (349).

By leveraging this phenomenon, researchers have successfully employed IRAE for the rapid and cost-effective isolation of flavonoids such as quercitrin, isoquercitrin, and rutin from *Magnolia officinalis* leaves. This approach is particularly valued for its operational simplicity, high extraction efficiency, and ability to produce flavonoid-enriched extracts with elevated concentrations of bioactive constituents (348). In another study, Wang et al. (349) pioneered an infrared-assisted self-enzymolysis extraction technique for the efficient isolation of total flavonoid aglycones, specifically oroxylin A, wogonin, and baicalein, from *Scutellariae radix*. In a comparative study, Cheaib et al. (350) evaluated various extraction techniques, including ultrasonic, microwave, and infrared, for the recovery of polyphenols from apricot pomace. Infrared extraction proved superior, producing the highest levels of total polyphenols (10 mg GAE/g DM), flavonoids (6 mg CE/g DM), and tannins (3.6 mg/L) (350).

IRAE consistently produced higher concentrations of key bioactive compounds, notably epicatechin, catechin, and rutin, demonstrating its efficacy in enhancing the recovery of polyphenolic constituents (351). The method's efficacy was further shown in polyphenol extraction from *Salviae miltiorrhizae* (danshen), where it enhanced the antioxidant capacity by 68% (from 47 to 79%) and increased the polyphenol concentration by 58% (from 0.12 to 0.19 mM) within a 30-min processing window. These results represent a significant improvement over traditional solid-liquid extraction techniques (351).

Chen et al. (351) also effectively isolated eight bioactive polyphenols from danshen (*Salvia miltiorrhiza*), specifically danshensu, protocatechuic acid, protocatechuic aldehyde, salvianolic acid B, dihydrotanshinone, cryptotanshinone, tanshinone I, and tanshinone IIA. This achievement underscores the versatility of modern extraction technologies in obtaining high-value phytochemicals from medicinal botanicals (351).

Similarly, a study by Abi-Khattar et al. (352) demonstrated the effectiveness of IRAE in recovering polyphenolic compounds, particularly oleuropein and hydroxytyrosol, from olive leaves, highlighting its potential as a rapid and efficient alternative to conventional methods (352). Their results showed a 30% increase in total phenolic content using IRAE compared to conventional water bath methods, resulting in a 27% reduction in ethanol consumption (352). Cao et al. (353) showed that IRAE offers distinct advantages over traditional techniques, including faster processing times, cost efficiency, higher extraction yields, and improved environmental sustainability. These benefits arise from the uniform radiative heating of samples, which enhances thermal efficiency and reduces energy waste (353).

#### 6.2.8 EAE

EAE has emerged as a promising alternative to conventional techniques, employing specific hydrolytic enzymes to enhance cell wall degradation and facilitate the efficient release of target phytochemicals from plant matrices. This technique progressively replaces traditional solvent-based extraction due to its superior safety profile, environmental compatibility, and extraction efficiency (354). The method's principal advantage resides in overcoming a fundamental limitation in plant-based extraction which is the structural resistance imposed by cell wall constituents, primarily cellulose, hemicellulose, and pectin (354). The method effectively degrades these structural components through the strategic use of enzymes, such as α-amylase, cellulase, hemicellulase, and pectinase. This breakdown enhances solvent penetration to bioactive compounds, ultimately boosting extraction yield and efficiency (354).

Deng et al. (355) demonstrated that combining short-wave infrared pre-treatment with enzyme-assisted aqueous extraction significantly improved peanut oil recovery efficiency. In a related study, Lenucci et al. (356) reported that pretreatment of freeze-dried tomato samples with glycosidase enzymes before supercritical CO_2_ extraction resulted in a threefold increase in lycopene yield (356). Boulila et al. (357) demonstrated that enzymatic pretreatment using cellulase, hemicellulase, and xylanase—either individually or in combination—significantly improves essential oil recovery from bay leaves. In a separate study, Sahne et al. (358) achieved enhanced curcumin extraction yields from turmeric by applying an α-amylase and amyloglucosidase enzyme cocktail.

In another study, Xu et al. (359) conducted a comparative study evaluating two extraction methods for polysaccharides from grape pomace: conventional ethanol extraction in comparison to enzyme-assisted extraction using a cellulase-pectinase-β-glucosidase cocktail. Their results demonstrated that the enzymatic approach provided dual advantages, increasing the yield of pectin. It preserved higher concentrations of phenolic compounds, particularly anthocyanins, while significantly reducing processing time compared to solvent-based extraction. Similarly, Vasco-Correa and Zapata (360) demonstrated that enzymatic treatment using protopectinase yields significantly higher pectin quantities from passion fruit peel than traditional chemical extraction methods (360). Roda et al. (361) demonstrated that enzyme cocktails comprising cellulase, hemicellulase, and pectinase effectively facilitate vinegar extraction from pineapple peel waste, a valuable byproduct in sugar manufacturing.

However, despite their improved extraction efficiency, advanced enzymatic methods may induce significant structural alterations in target bioactive compounds due to enzymatic bond cleavage, which can potentially affect their stability and biological activity (362, 363).

#### 6.2.9 Natural deep eutectic solvents extraction (NDESE)

NDESE represents a revolutionary green extraction approach using naturally occurring compounds to form eutectic mixtures with unique solvating properties. Recent research by Ristivojević et al. (364) highlighted NDESE as a sustainable alternative to conventional organic solvents, offering biodegradability, low toxicity, and tunable extraction selectivity (364). Other studies also demonstrate that choline chloride-based NDESE can achieve extraction recoveries of 88.91%−98.99% for quercetin from plant sources, making them highly effective for polyphenolic compound extraction (365, 366).

#### 6.2.10 ILE

ILE has gained significant attention due to the tunable physicochemical properties of ionic liquids, allowing for highly selective extraction processes. Recent advances focus on developing environmentally sustainable ionic liquids with reduced synthesis costs and improved recyclability (367). The technology shows particular effectiveness for extracting specific bioactive compound classes while maintaining high selectivity and minimal environmental impact (368, 369).

#### 6.2.11 Emerging hybrid and green technologies

Current extraction research increasingly focuses on hybrid approaches that combine conventional and innovative methodologies (370), as systematically compared in Table 2.

##### 6.2.11.1 Advanced integration strategies

Recent research emphasizes hybrid extraction approaches that combine multiple techniques to maximize efficiency (370). The integration of EAE-UAE has demonstrated remarkable synergistic effects, with enzymes creating porous cellular structures that allow ultrasound to penetrate more effectively (370). These combined approaches yield higher results at lower temperatures and shorter processing times than individual methods (237, 370).

##### 6.2.11.2 Green solvent evolution

The field has witnessed significant advancement in green extraction solvents. Deep eutectic solvents, derived from natural compounds such as choline chloride, organic acids, and sugars, offer environmentally friendly alternatives to traditional volatile organic solvents (366, 370). These solvents demonstrate not only reduced environmental impact but also often enhanced extraction efficiency and compound stability (364, 366, 370).

### 6.3 Industrial scale-up and process optimization: automation and integration of AI

Advanced process optimization, utilizing AI and machine learning, has become crucial for maximizing extraction efficiency (370). Automated process control systems enable real-time monitoring and optimization of extraction parameters, significantly improving reproducibility and throughput (370). These technological advances address traditional challenges of parameter optimization and process control (237, 370).

### 6.4 Economic and environmental considerations

Recent studies emphasize the economic viability of advanced extraction methods through process intensification and energy recovery systems (371). While initial equipment costs remain high for technologies like SFE and PEFE, improved efficiency and reduced environmental impact provide long-term economic benefits (371, 372). The development of modular and scalable equipment designs has made advanced extraction technologies more accessible for various production scales (237, 353).

### 6.5 Future and emerging trends

Biorefinery concepts are increasingly being applied to extraction processes, maximizing the utilization of raw materials and by-products (237). This approach integrates extraction with downstream processing to extract maximum value from plant sources while minimizing waste generation (237, 371). Nanotechnology integration in extraction processes shows promise for enhanced selectivity and efficiency. Nanomaterials can improve adsorption and separation processes, while nanocarriers enable targeted delivery of extracted bioactive compounds (237).

### 6.6 Critical assessment of recent developments

Performance advantages of modern methods over traditional approaches have become more pronounced with recent technological improvements. Modern extraction techniques consistently demonstrate a 50%−80% reduction in extraction time, a 30%−70% decrease in solvent consumption, and a 20%−50% improvement in extraction yields compared to conventional methods (353, 373).

Sustainability metrics have become central to the evaluation of extraction methods, with life cycle assessment studies showing that, despite higher initial investments, modern extraction methods provide superior environmental performance through reduced solvent use, energy consumption, and waste generation (364, 368). Commercial viability continues to improve as equipment costs decrease and production scales increase. The growing availability of industrial-scale equipment for technologies like UAE, MAE, and PEFE has made these methods increasingly practical for commercial applications (353, 370, 372).

## 7 Immobilization of bioactive molecules

Bioactive compounds are non-nutritional food components that modulate metabolic processes and confer health benefits (374). They exhibit diverse therapeutic properties, including pancreatic lipase inhibition for the management of obesity (375), free radical neutralization (376), and anticancer activity (377). However, their practical utilization faces several challenges. Naturally occurring bioactive compounds, such as polyphenols and phytosterols found in fruits and vegetables, often exhibit undesirable sensory characteristics, including bitterness and astringency, which can reduce consumer acceptance. Furthermore, they frequently suffer from poor bioavailability, low bioaccessibility, instability under thermal or light exposure, and high volatility (378). These constraints significantly limit their functional applications (379). For instance, heat- and oxidation-sensitive compounds, such as vitamin C, may degrade during digestion or gastrointestinal transit (379, 380).

Bioactive compounds are highly susceptible to physicochemical degradation during food processing, storage, and digestion, compromising their functionality (381). To address this, microencapsulation has emerged as an effective strategy to stabilize these compounds, mask undesirable sensory properties, and improve bioavailability (382, 383). Recent advancements have facilitated the development of innovative encapsulation technologies designed to enhance the targeted delivery of bioactive compounds to specific physiological sites, thereby improving their stability, bioavailability, and therapeutic efficacy (384).

Emulsion formation, suspension, particle and gel preparation, hydrogel and microgel fabrication, liposome production, and coacervation have been optimized to create tailored delivery systems for bioactive food components (385–387). Compared to other delivery approaches, encapsulated bioactive compounds in particulate form offer distinct advantages due to their small and uniform size, ensuring efficient delivery to target areas. Encapsulation maintains the bioactivity of these compounds during both storage and digestion while improving their stability as they pass through the gastrointestinal tract (200). The process works by incorporating bioactive agents into a protective matrix, commonly known as a wall material or encapsulant (388, 389).

This technique generates micro- or nano-sized capsules, where bioactive compounds (known as the core, payload, or internal phase) act as functional agents, while wall materials (also termed the membrane, shell, coating, matrix, or external phase) create a protective barrier (390). Widely employed in the food and pharmaceutical industries, encapsulation serves as an effective strategy to protect sensitive bioactive compounds, such as polyphenols, micronutrients, enzymes, and antioxidants, from degradation caused by environmental and processing stresses (391). The protective matrix shields these compounds from degradation caused by environmental factors, including light, oxygen, pH variations, moisture, heat, mechanical stress, and other destabilizing conditions (390–392).

### 7.1 Bulk encapsulation assisted by ultrasound

Ultrasound technology is generally classified into two categories based on acoustic intensity: low-intensity ultrasound, primarily used for analytical and imaging purposes, and high-intensity ultrasound, which is applied for physical and chemical modifications, including extraction processes (393). Low-intensity ultrasound (typically using frequencies above 1 MHz at power intensities below 1 W/cm^2^) functions as a non-destructive analytical method for evaluating food components' physicochemical properties (393). This technique provides precise measurements while preserving the structural integrity of the material, making it particularly valuable for non-invasive food characterization (393, 394).

High-intensity ultrasound has become the leading technology for food processing and preservation (289). This method effectively alters the physicochemical properties of food components. Ultrasound technology plays a pivotal role in various applications, including the extraction of bioactive compounds, the modification of crystal structures, the inactivation of enzymes, the disruption of cellular matrices, equipment sanitation, emulsion formation, and other industrial processes (395). Beyond food applications, ultrasound technology exhibits remarkable versatility in creating catalytic and functional materials across diverse sectors (396, 397).

Its applications extend to medical imaging (290, 398), energy generation, and therapeutic/diagnostic medicine (399, 400). These varied applications primarily rely on acoustic cavitation—the generation and implosion of microbubbles induced by ultrasonic waves (401, 402). The versatility of ultrasound technology arises from its broad operational frequency range, which allows precise modulation of cavitation intensity and acoustic frequency (403). This controllability facilitates the fine-tuning of material properties, including particle size distribution, surface morphology, and structural integrity (403). Additionally, ultrasound can enhance drug absorption through encapsulation, a method designed to protect, prolong shelf life, or stabilize encapsulated substances against environmental degradation (403).

This method improves the bioavailability and therapeutic efficacy of drugs and nutrients by facilitating their absorption, stability, and targeted delivery within biological systems. Among various delivery platforms, food emulsions represent a particularly effective system (404, 405). Ultrasound technology has emerged as a preferred extraction technique due to its environmental sustainability, cost efficiency, rapid processing, and high yield of phenolic compounds, advantages primarily derived from the acoustic cavitation phenomenon produced by ultrasonic waves (406, 407).

### 7.2 Mass encapsulation through spray drying

Spray drying is the most established and extensively utilized encapsulation technique in the food industry, owing to its scalability, cost-effectiveness, and ability to produce stable, dry powders containing bioactive compounds (408). This versatile, continuous process offers cost-efficient production of encapsulated particles with diameters ranging from several micrometers to tens of micrometers, while maintaining a consistent particle size distribution (409, 410). Through spray drying encapsulation, bioactive compounds are protected and stabilized, and their solubility and controlled release are improved, ultimately delivering them in a convenient powdered form (411, 412).

As the predominant encapsulation method in food applications, spray drying encapsulates functional compounds within an inert carrier matrix (413, 414). This process improves microbiological stability while reducing storage and transportation costs through moisture reduction and water activity control, thereby minimizing chemical and biological degradation (415, 416). The technique offers notable advantages, including continuous, cost-effective operation with rapid processing times, and utilizes pressure, rotary, or twin-fluid nozzles. However, challenges include inconsistent droplet size distribution, limited control over particle uniformity, and potential nozzle clogging when handling suspensions (417, 418).

During operation, the rapid drying mechanism creates a protective dry layer around bioactive compounds (419). However, the high temperatures required for rapid water evaporation expose sensitive core materials to thermal stress, potentially degrading heat-labile compounds (420). To mitigate this, protective polymeric coatings, often proteins or carbohydrate-based, are combined with the bioactive solution to act as a thermal barrier (421, 422). Although energy-intensive, spray drying remains a highly scalable and efficient encapsulation method, capable of producing nano- to micron-sized particles with a narrow size distribution within a relatively short processing time (409, 423).

Spray drying is a phase-transition process that converts liquid feed into solid particles through atomization and rapid drying. This technique employs atomization to enhance drying efficiency by generating fine sprays, significantly increasing evaporation rates. Unlike conventional methods, spray drying achieves faster drying times (424, 425) while maintaining lower product temperatures due to evaporative cooling. Widely employed in both the food and pharmaceutical industries, spray drying is commercially utilized to produce a diverse range of powdered products, including milk, whey protein isolates, instant coffee, and tea extracts (426).

Beyond simple dehydration, it has evolved into a versatile method for microencapsulation, microbial inactivation, shelf-life extension, and product quality enhancement. However, a key limitation is its reliance on high temperatures, making it unsuitable for heat-sensitive compounds such as volatile aromas or bioactive substances (427, 428).

Extensive research has been dedicated to the encapsulation of bioactive compounds such as flavors, lipids, polyphenols, and pigments (e.g., carotenoids) (429). The efficiency of the encapsulation process and the quality of the final product are primarily influenced by key operational parameters, including inlet and outlet air temperatures, feed temperature, flow rate, and the physicochemical properties of the emulsion (429). These emulsion characteristics are, in turn, governed by multiple factors, including the composition of the oil phase, selection of wall material, core-to-wall ratio, total solids content, fluid viscosity, and the size and stability of dispersed droplets (430, 431).

### 7.3 Big batch encapsulation using spray chilling

Spray chilling involves the atomization and subsequent solidification of droplets to form encapsulated particles, sharing fundamental similarities with spray drying (432). The spray drying process comprises three fundamental components: an atomization unit that converts the liquid feed into fine droplets, a drying chamber where solvent evaporation and particle formation occur, and a collection system for recovering the dried, encapsulated particles (433, 434). During operation, a mixture of bioactive compounds and molten lipid carrier is atomized into a chilled chamber maintained below the lipid's melting point, where contact with cold air promotes rapid solidification into lipid microparticles that effectively encapsulate and preserve the active ingredients (435, 436).

The fundamental distinction between encapsulation techniques occurs during particle formation, where solidification occurs through droplet cooling or solvent evaporation (437). The process begins by dispersing active ingredients (flavors, vitamins, oils, or bioactive compounds) in a liquid matrix (waxes, fats, lipids, or hydrocolloids) before atomization. Upon cooling, the matrix solidifies to form microspheres or multi-core microcapsules (438). While spray freezing shares similar principles with spray drying (439, 440). Spray chilling employs cold air atomization rather than hot air (441, 442).

A notable limitation in encapsulating lipophilic substances is their potential inability to effectively mask undesirable flavors due to miscibility with the matrix (443). This challenge can be mitigated by employing non-miscible carriers such as sugar alcohols; for example, sorbitol was among the first crystallizing agents used to encapsulate and stabilize flavor compounds (443, 444). Spray freezing remains a proven lipid-based microparticle production method using spray dryer equipment (445, 446), while spray cooling with hydrophobic materials is gaining traction in food and pharmaceutical applications for producing smooth, spherical microspheres with uniform active ingredient distribution (447). Unlike many other microencapsulation techniques, spray cooling avoids high temperatures and ensures efficient release. It is known for being fast, user-friendly, and cost-effective, making it a preferred method for encapsulating heat- or moisture-sensitive functional compounds (448, 449).

### 7.4 Post-coating fluidized bed

Fluid bed coating is an encapsulation method that deposits protective layers onto powdered substrates, making it adaptable for both batch and continuous processing (450). The technique atomizes coating material onto fluidized particles to form uniform encapsulations (451, 452). Critical process parameters, such as nozzle atomization pressure, solid circulation rate, coating feed rate, and temperature, play a pivotal role in preventing particle agglomeration and ensuring uniform film formation.

As demonstrated by Guignon et al. (453), these factors ultimately govern coating efficiency and product quality. Therefore, precise optimization of processing parameters is essential to achieve uniform, high-quality coatings when employing fluidized bed technology (453). Various coating materials, such as gums, proteins, and starches, can be employed, making this method increasingly valuable for delivering a wide range of encapsulated food ingredients and additives to the industry (454).

Fluidized-bed coating utilizes different spray configurations, including top-spray, bottom-spray, and tangential spray methods (455). Three primary categories of factors influence the performance of the process: (a) operational parameters, including inlet air temperature, air velocity, spray rate, and atomization pressure; (b) environmental conditions, such as ambient temperature and relative humidity; and (c) thermodynamic variables, including outlet air temperature and moisture content (456).

The technique relies on suspending particles in a gas stream, allowing atomized shell material droplets to coat each particle individually (457, 458). Upon contact, these droplets form a protective layer. The gas stream performs two critical functions simultaneously: sustaining particle fluidization while supplying the energy required for solvent evaporation or coating solidification. This dual mechanism enables the precise regulation of the microcapsules' protective characteristics and release properties (459). Conventional fluidized bed systems typically employ a single-pass gas configuration, where the processing gas circulates through the bed only once before being discharged into the atmosphere (460, 461).

Fluidized bed coating is gaining prominence in the food industry for encapsulating ingredients and additives (454). Unlike pharmaceutical applications, where precision often justifies higher costs, food technologists must prioritize cost-effectiveness, requiring modified approaches for this relatively expensive technology (462, 463). While the pharmaceutical sector has extensively used fluidized-bed coating for drug formulations, creating films with controlled release, taste masking, enteric protection, enhanced stability, and improved appearance, the food industry adapts these principles with greater emphasis on economic feasibility (464, 465).

Fluidized bed coating is a versatile technique for optimizing, controlling, or altering the performance of functional ingredients and additives (466). Its applications span various food components, including processing aids (e.g., leavening agents and enzymes), preservatives (such as acids and salts), nutritional enhancers (like vitamins and minerals), and both natural and artificial flavorings (463). Coating materials, also referred to as shells, walls, or membranes, are composed of a wide range of natural or synthetic film-forming polymers, whose physicochemical properties have been extensively investigated in the context of edible coatings (467, 468).

Microencapsulation offers significant advantages, including extended product stability, taste concealment, easier processing, controlled release, and improved visual appeal, flavor, and coloration (469, 470). While pharmaceutical applications prioritize precision, the food industry's adoption of fluidized-bed coating technology emphasizes cost reduction in production (471).

### 7.5 Encapsulation through bulk lyophilization

Freeze-drying, also known as lyophilization, is a dehydration technique that involves freezing the sample and then removing the ice via sublimation under reduced pressure (472). The process consists of two key phases: primary drying, where sublimation occurs under low temperatures and moderate vacuum, and secondary drying, which involves desorption at higher shelf temperatures and lower chamber pressures (473, 474). Particularly suited for heat-sensitive materials, freeze-drying serves as an excellent method for microencapsulation (456, 475), operating through four key phases: freezing, sublimation, desorption, and storage stabilization (476, 477).

This dehydration technique is particularly valuable for heat-sensitive food and biological materials, utilizing sublimation to achieve extended shelf life while preserving essential qualities such as structural integrity, organoleptic properties (including taste, color, aroma, and texture), and bioactivity (478, 479). The composition and structural characteristics of the wall material predominantly govern the protective efficiency and release kinetics of encapsulated compounds (480, 481), with commonly used encapsulants including gum Arabic, maltodextrin, modified starches, whey proteins, and related biopolymers (481).

Despite its advantages, freeze-drying has notable limitations, including high energy consumption, prolonged processing times (482), and the formation of porous matrices, which may hinder sustained release performance (436, 483). While effective for shelf-life extension, the method suffers from substantial capital/operational expenses and offers limited control over final particle size distribution (484, 485).

## 8 Health benefits of plant materials

**Figure 7:**
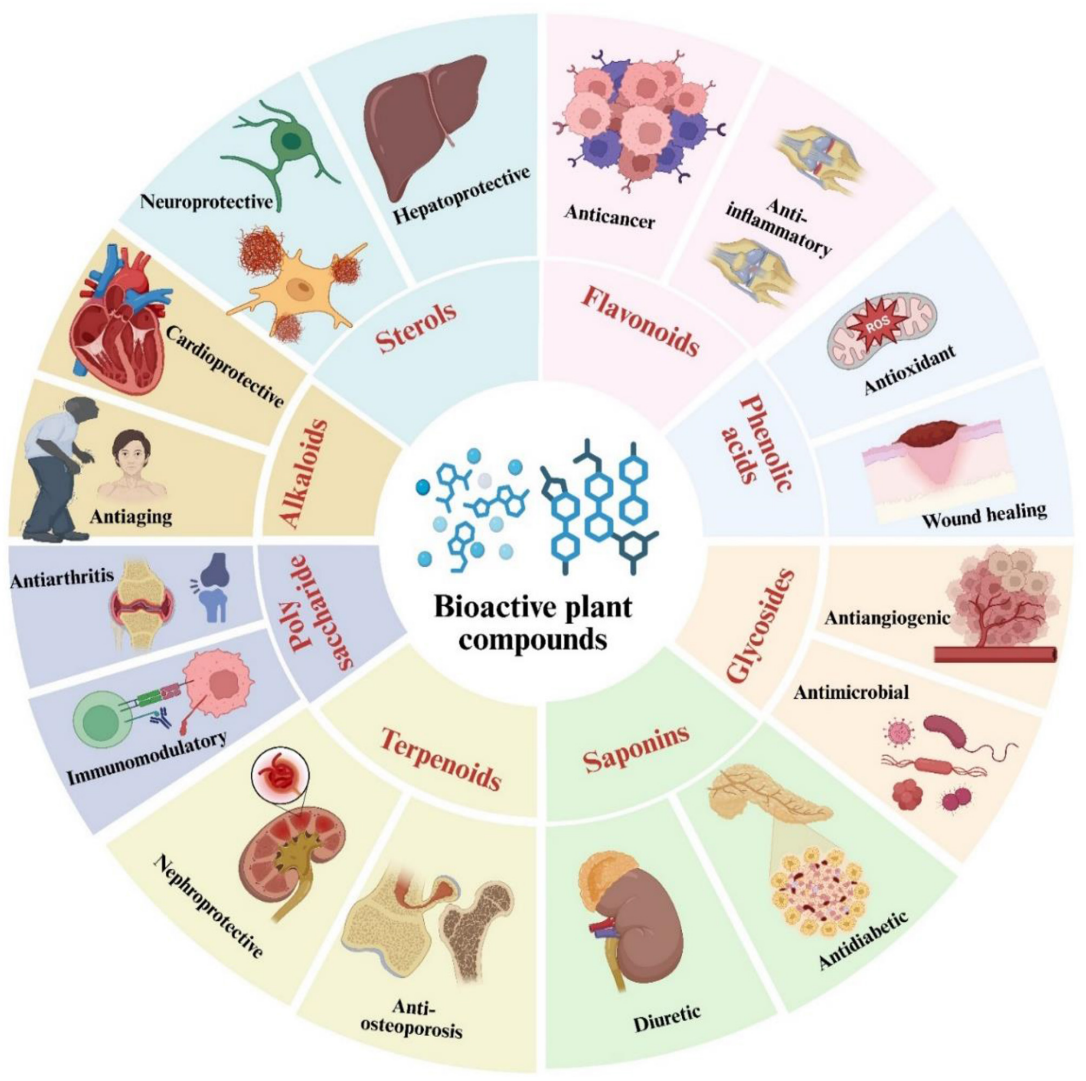
The therapeutic potential of bioactive plant compounds, emphasizing their involvement in disease prevention and management via antioxidant, anti-inflammatory, antibacterial, antidiabetic, neuroprotective, and cardioprotective actions.

Plants synthesize a wide array of bioactive compounds with therapeutic potential, typically characterized by a predominant class of phytochemicals responsible for their principal health-promoting effects (Table 3 and Figure 7). Figure 7 illustrates the therapeutic potential of bioactive plant compounds, highlighting their roles in disease prevention and management through their antioxidant, anti-inflammatory, antibacterial, antidiabetic, neuroprotective, and cardioprotective properties.

**Table 3:** Bioactive components derived from diverse plant sources that enhance health and their corresponding therapeutic benefits.

**Bioactive**	**Constituents**	**Applications/health benefits**	**Mechanism of action**	**References**
Kiwis	• Hydroxycinnamic acids (caffeic acid) • vitamin C, polyphenols	Antioxidant, immune support	Scavenges reactive oxygen species; enhances collagen synthesis	(655)
Plums	• Coumaric acid • anthocyanins, chlorogenic acid	Anti-inflammatory, antioxidant	Modulates cytokines; reduces lipid peroxidation	(655)
Wheat grains	• Caffeic acid • ferulic acid, alkylresorcinols	Colon health, antioxidant	Inhibits oxidative damage; modulates gut microbiota	(656)
Bananas	Phenolic acids (syringic, vanillic, p-coumaric, salicylic, ferulic, sinapic, phydroxybenzoic, and gallic acids)	Antioxidant, mood support	Acts as reactive oxygen species scavenger; modulates neurotransmission	(657)
Green tea and red wine	• Epigallocatechin gallate • catechins and their gallates • catechins, resveratrol	Cardioprotective, anti-obesity, anti-diabetic, anti-carcinogenic	Epigallocatechin gallate (EGCG) activates AMP-activated protein kinase (AMPK), promoting fat oxidation and improving insulin sensitivity. It induces apoptosis in cancer cells, inhibits angiogenesis (via VEGF suppression), and reduces lipid peroxidation, acting as a potent antioxidant. Also, it enhances endothelial function; activates SIRT1	(658)
Celery and red pepper	• Flavones (apigenin, and luteolin) • apigenin, capsaicin	Anti-inflammatory, antioxidant	Inhibits COX-2 and NF-κB	(658)
Maize seed	Anthocyanins (pelargonidin, and cyanidin) phenolic acids, carotenoids	Vision support, antioxidant	Neutralizes free radicals; protects macular pigments.	(512)
Blueberry	Anthocyanins (delphinidin, cyanidin, petunidin, peonidin, and malvidin), flavonols	Cognitive function, anti-aging	Reduces neuroinflammation; protects neurons	(517)
Soybean	Isoflavonoids (genistein, and daidzein)	Cancer treatment	Isoflavones such as genistein and daidzein bind to estrogen receptors (ERα and ERβ), exerting estrogen-like effects. They inhibit tyrosine kinases, modulate the PI3K/Akt signaling pathway, and induce cell cycle arrest and apoptosis in cancer cells	(659)
Lettuce	Chlorogenic acid, polyphenols (cyanidin, and quercetin) Lutein, beta-carotene	Eye health, antioxidant	Filters blue light; neutralizes reactive oxygen species	(660)
Pumpkin	Carotenoids (β-carotene and lycopene) beta-carotene, tocopherols	Prostate support, antioxidant	Quenches singlet oxygen; modulates androgen activity	(498)
Grapes and citrus fruits	Flavanone (naringenin)	Anti-inflammatory, antioxidant	Resveratrol activates SIRT1, a key regulator of metabolic and aging pathways, enhancing mitochondrial function and cellular resilience. It also inhibits inflammatory enzymes (COX-2, iNOS), reduces reactive oxygen species formation, and prevents platelet aggregation and low-density lipoprotein oxidation	(661)
Berries	• Anthocyanins (cyanidin and delphinidin) • polyphenols, ellagic acid	Cognitive protection, antioxidant	Suppresses neuroinflammation; protects DNA	(662)
Black currants	Anthocyanins, including cyanidin-3-glucoside and delphinidin-3-glucoside, along with vitamin C	Eye health, anti-fatigue	Improves microcirculation; enhances retinal function	(519)
Hazelnut	Myricetin, syringetin, proanthocyanidins A and B, vitamin E, oleic acid	Cardiovascular health	Protects low-density lipoprotein from oxidation and supports the endothelium	(663)
Papaya peels	The identified compounds include the phenolic acids: caffeic acid, p-coumaric acid, and ferulic acid, alongside vitamin C	Wound healing, anti-inflammatory	Stimulates tissue repair; inhibits prostaglandin	(33)
Mango peels	The polyphenols gallic acid, chlorogenic acid, syringic acid, catechin, quercetin, and kaempferol	Antidiabetic, antioxidant	Inhibits α-glucosidase; reduces reactive oxygen species	(40)
Apple pomace	The plant is rich in beneficial compounds, including flavonoids such as quercetin, isorhamnetin, and procyanidin catechin, as well as phenolic compounds like chlorogenic acid and p-coumaroylquinic acid	Gut health, antioxidant	Improves digestion; binds toxins	(664)
Tomato peels	polyphenols (p-coumaric acid, chlorogenic acid, and quercetin), and carotenoids (lycopene)	Antioxidant, skin protection	Lycopene quenches singlet oxygen and neutralizes free radicals. It downregulates IGF-1 signaling, known to promote cancer cell growth, and protects DNA from oxidative damage. It also improves endothelial function and inhibits LDL oxidation	(665)
Lemon seeds	A group of flavonoids, including gallocatechin, caffeic acid, epicatechin, vitexin, quercetin, and hesperidin	Antimicrobial, anti-inflammatory	Disrupts bacterial membranes; modulates COX	(666)
Beetroot	Polyphenols (betagarin, betavulgarin, and cochliophilin A), and betalains	Blood pressure regulation, antioxidant	Betalains enhance nitric oxide bioavailability, thereby improving vasodilation and lowering blood pressure. They act as scavengers of reactive species and downregulate pro-inflammatory cytokines, contributing to cardiovascular and anti-inflammatory benefits	(667)
Olive leaves	The phenolic profile is characterized by the presence of rutin, tyrosol, luteolin, p-coumaric acid, ferulic acid, quercetin, oleuropein, and hydroxytyrosol, which are commonly identified in olive-derived matrices and related products	Cardioprotective, anti-inflammatory	Oleuropein inhibits inflammatory mediators, such as NF-κB, and reduces oxidative stress by enhancing the activity of enzymes like superoxide dismutase (SOD). It also shows antibacterial and antiviral properties through membrane disruption and inhibition of viral replication	(50)
Grape pomace	Flavonoids (sinapic acid, rutin, and epicatechin); phenolic acids (caffeic acid, gallic acid, and quercetin)	Antioxidant, gut health	Binds bile acids; promotes beneficial microorganisms	(507, 668)
Potato peel	phenolic acids (vanillic acid, gallic acid, isoferulic acid, chlorogenic acid, and caffeic acid)	Antioxidant, cholesterol-lowering	Inhibits lipid oxidation; reduces low-density lipoprotein	(582)
Jujube peel	Flavonoids (quercetin, and rutin) saponins, triterpenoids	Sedative, immune modulation	Modulates GABA receptors and enhances phagocytosis	(669)
Pomegranate peel	Flavonols (quercetin and kaempferol); Anthocyanins (cyanidin, delphinidin, and pelargonidin glycosides); punicalagins	Antioxidant, anti-inflammatory	Punicalagins exhibit potent antioxidant activity by neutralizing ROS and enhancing endogenous antioxidant enzymes. They suppress NF-κB activation, downregulate inflammatory markers, and trigger apoptosis in various cancer cell lines	(670)
Garlic	Allicin, sulfur compounds	Antimicrobial, antihypertensive, cholesterol-lowering	Inhibits HMG-CoA reductase; disrupts microbial cell membranes	(671)
Broccoli	Sulforaphane, glucosinolates	Detoxification, anticancer, anti-inflammatory	It activates Nrf2, induces phase II enzymes, and inhibits histone deacetylases	(672)
Flaxseed	Lignans, omega-3 fatty acids	Hormonal balance, cardiovascular health, and anticancer	Modulates estrogen metabolism; reduces inflammation	(673)

A notable example is the apple (*Malus domestica* L.), which contains high concentrations of flavonoid antioxidants, including epicatechin, phloretin, and quercetin, as well as phenolic acids like chlorogenic acid and coumaroylquinic acid (486). Mandarin fruit pulp contains high levels of antioxidants (ascorbic acid, carotenoids, and phenolics), along with carbohydrates, minerals, and aromatic amino acids (487), while their peels are an excellent source of polyphenols (488). The health-promoting properties of mandarins are primarily attributed to their bioactive compounds, which exhibit potent antioxidant activity. Similarly, prickly pear (*Opuntia* spp.) represents a nutritionally dense fruit source, containing high levels of polyphenols, betalains, ascorbic acid, essential minerals, and amino acids (489). These constituents demonstrate multiple biological activities, including antioxidant effects, antiatherogenic and antiulcerogenic properties, and inhibition of LDL peroxidation (490, 491). Berry fruits, such as blueberries (*Vaccinium* spp.), blackberries (*Rubus* spp.), strawberries (*Fragaria* × *ananassa*), and grapes (*Vitis* spp.), are similarly rich in antioxidant compounds, with significant concentrations present in their extracts (492).

Research has demonstrated that polyphenols and ascorbic acid exert protective effects against a range of chronic diseases, including pulmonary disorders, rheumatoid arthritis, cardiovascular diseases, Parkinson's disease, and Alzheimer's disease (493, 494). Among vegetables, *Allium* species (garlic, onions, chives, and leeks) are particularly noteworthy due to their rich content of organosulfur compounds, as well as significant concentrations of flavonoids, steroidal saponins, and phytosterols (495). These bioactive components contribute to various therapeutic properties, such as immunomodulation, antiviral activity, blood glucose regulation, oxidative stress reduction, cancer prevention, inflammation suppression, and neural protection (496, 497). Similarly, Montesano et al. (498) reported that pumpkin (*Cucurbita* spp.) is rich in bioactive compounds, particularly terpenoids such as carotenoids, which demonstrate multiple health benefits, including immune system enhancement, a reduced risk of cancer and cardiovascular diseases, and support for prostate health. Similarly, scientific studies have identified rosemary (*R. officinalis* L.) as containing potent bioactive components that exhibit various therapeutic properties, including antifungal, antidepressant, antidiabetic, anti-inflammatory, and antithrombotic effects (499, 500). Extracts derived from medicinal and aromatic plants hold GRAS status, making them viable natural alternatives to synthetic additives (501). Sage (*Salvia officinalis* L.), for instance, demonstrates significant potential as a functional food additive, with documented anti-inflammatory, anticancer, antimicrobial, and antiproliferative activities (502).

Studies have demonstrated that *Salvia officinalis* L. (sage) possesses multiple therapeutic properties, including anti-inflammatory, anticancer, antimicrobial, and antiproliferative effects (502, 503). Wu et al. (504) reported that oregano (*Origanum vulgare*) possesses significant antibacterial and antioxidant capabilities. Similarly, *Thymus vulgaris* L. (thyme) contains several biologically active compounds, including thymol, carvacrol, geraniol, and *p*-cymene, which contribute to its therapeutic properties (505). These compounds exhibit neuroprotective effects, support respiratory health, and possess notable antibacterial activity (506).

Grape pomace, a byproduct of winemaking, is a cost-effective and nutritionally valuable source of bioactive compounds, including flavonoids, phenolic acids, and lignans (507). Research has demonstrated its therapeutic potential in managing various health conditions, including hypertension, atherosclerosis, neurodegenerative disorders, and cardiovascular diseases (507). Similarly, citrus processing generates significant byproducts abundant in flavonoids, limonoids, and essential oils (508).

The antioxidant properties of citrus waste stem from its bioactive components, while its essential oils demonstrate potent antibacterial, antifungal, and antiviral activities (508). *Stevia rebaudiana* serves as a remarkable natural reservoir of bioactive compounds, notably polyphenols, carotenoids, ascorbic acid, and chlorophylls (509, 510). Extensive research has established that stevia extracts exhibit diverse pharmacological effects, including potent antioxidant, antimicrobial, antihypertensive, antineoplastic, immunomodulatory, and anti-inflammatory properties. As reported by Bulotta et al. (511), olive leaf extracts possess multiple therapeutic properties, including antiviral, antitumor, antioxidant, anticancer, antibacterial, and cardiovascular benefits. In another study, Toufektsian et al. (512) examined the cardioprotective effects of anthocyanin-fortified maize in male Wistar rats using an 8-week dietary regimen (20% seed inclusion). Their results indicated a statistically significant (*P* < 0.01) reduction in myocardial infarct size after coronary occlusion-reperfusion injury compared to control diets, supporting the potential cardiovascular benefits of these phytochemicals (512).

Afshari et al. (513) evaluated the anticancer properties of eggplant extract using human gastric cancer cell lines, demonstrating significantly greater cytotoxic effects on malignant cells than normal cell lines. The study attributed these anticancer properties to the extract's potent antioxidant activity and high phenolic content, which may contribute to the neutralization of free radicals. These findings suggest that incorporating eggplant into the diet could serve as a preventive strategy against cancer development (513). Plant-derived polyphenols demonstrate anticancer potential by reversing harmful epigenetic alterations in malignant cells, suppressing tumor growth, blocking metastatic spread, and increasing tumor sensitivity to radiotherapy and chemotherapy (514).

As stated by Sharma et al. (515), pomegranate byproducts and waste extracts exhibit preventive and therapeutic effects against various types of cancer. Specifically, pomegranate extract suppresses prostate cancer cell proliferation and triggers apoptosis by inhibiting the NF-κB pathway (516). Faria et al. (517) reported that anthocyanin-pyruvic acid adducts and blueberry extracts showed notable anticancer activity against MDA-MB-231 and MCF-7 breast cancer cell lines by inhibiting cancer cell invasion and proliferation (517). Plant-derived terpenoids and carotenoids exhibit significant anti-inflammatory and anticancer properties, primarily by inhibiting NF-κB signaling pathways that are pivotal in inflammatory processes and cancer progression (518).

Anthocyanin-rich black currants show potential in managing hyperglycemia, as demonstrated in Caco-2 cell models by Barik et al. (519). Anthocyanins derived from black currants (*Ribes nigrum*) have been shown to primarily regulate postprandial glucose metabolism by inhibiting α-glucosidase activity. Their research further demonstrated that complementary phenolic constituents in black currants modulate several glycemic control mechanisms, including: (1) inhibition of salivary α-amylase activity, (2) regulation of intestinal sugar transporter function, and (3) enhancement of cellular glucose uptake. These synergistic actions may collectively reduce the risk of type 2 diabetes, as evidenced by corroborative studies using streptozotocin (STZ)-induced diabetic murine models. Yang et al. (520) demonstrated the glucose-lowering potential of puerarin (an isoflavone compound), with their 4-week intervention study revealing significantly improved insulin levels and marked hypoglycemic effects in treated subjects.

In complementary research, Anhê et al. (521) investigated the anti-inflammatory properties of cranberry polyphenols in murine models. Their 8-week dietary intervention with polyphenol-rich cranberry extract showed (1) significant enrichment of *Akkermansia* spp. populations, (2) attenuation of high-fat/high-sucrose diet-induced effects, including visceral adiposity, and (3) reduction in both intestinal inflammation and excessive weight gain (521). Whole-cereal grains, rich in phenolic compounds and dietary fiber, demonstrate beneficial modulatory effects on gut microbiota composition, potentially contributing to improved metabolic health, according to Gong et al. (522).

Emerging evidence suggests that properly processed high-cereal diets may offer therapeutic potential for various metabolic disorders. In cancer research, avocado seed extracts show dose-dependent anti-inflammatory and antiproliferative activity against human colorectal carcinoma (HCT)-116 and hepatocellular carcinoma HepG2 cell lines (523). As documented by Mirza et al. (524) and Donga et al. (525), mango peel byproducts have been characterized as a valuable source of bioactive polyphenols, with protocatechuic acid and mangiferin being particularly noteworthy for their demonstrated antimicrobial, antidiabetic, anti-inflammatory, and anticancer properties.

Research has similarly identified fruit and vegetable processing byproducts, including apple (526), cauliflower (527), elderberry (528), citrus (529), and pomegranate (530), that showed significant antimicrobial activity against *Staphylococcus aureus*. Similarly, agricultural byproducts from artichoke (531), banana (532), grape (533), orange (534), pomegranate (535), and tomato (536) showed antimicrobial activity against a range of pathogenic bacteria.

**Figure 8:**
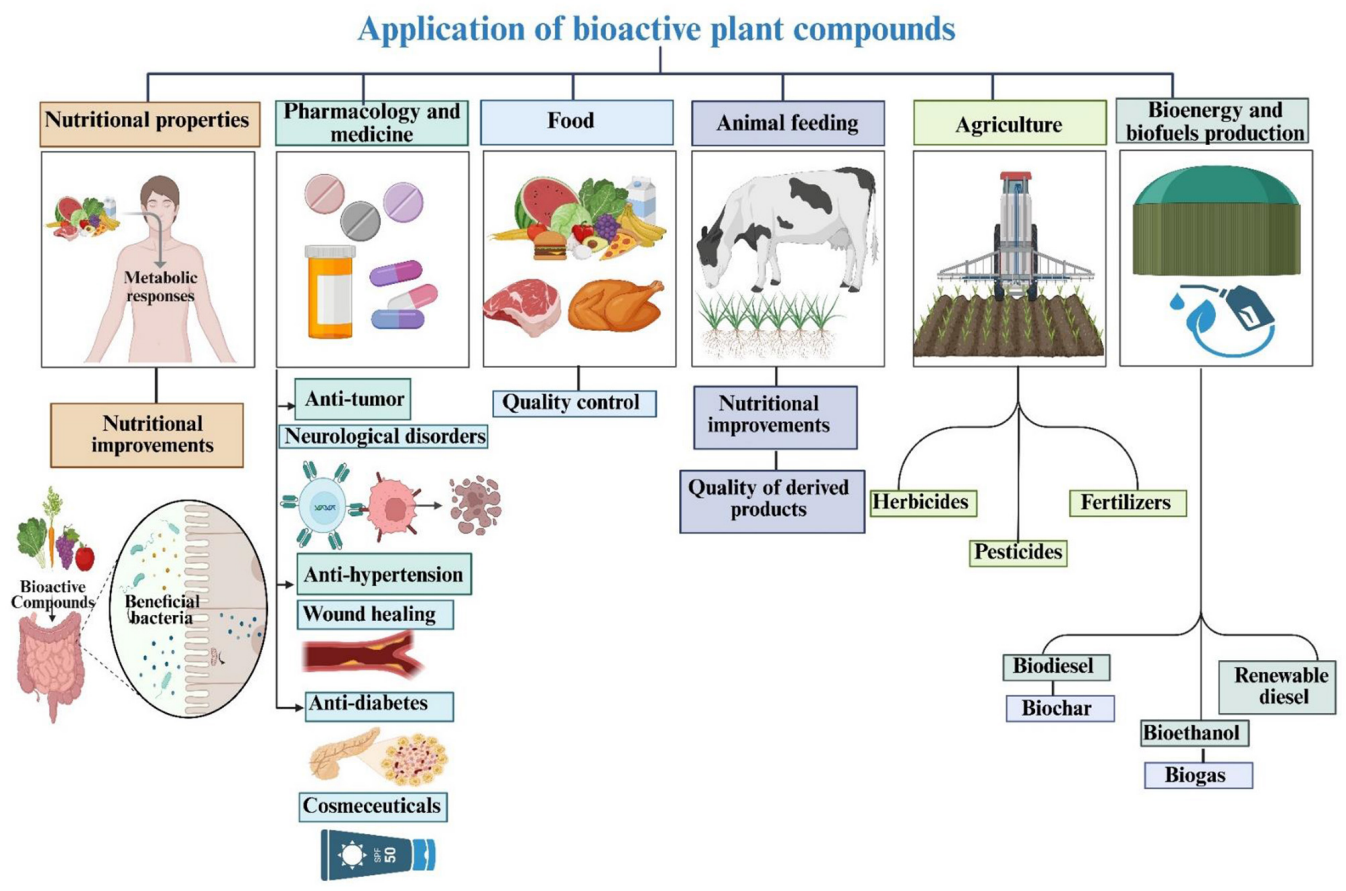
The main application areas of bioactive plant compounds encompass food preservation, functional foods, pharmaceuticals, nutraceuticals, cosmetics, and therapeutic formulations.

Table 3 illustrates bioactive components obtained from various plant sources that promote health and associated therapeutic advantages. Figure 8 demonstrates the main application areas of bioactive plant compounds, encompassing food preservation, functional foods, pharmaceuticals, nutraceuticals, cosmetics, and therapeutic formulations.

## 9 Plant-based functional food for human health

Global health awareness has reached unprecedented levels, accompanied by a growing emphasis on preventive healthcare strategies (537). Contemporary lifestyle challenges, such as occupational stress and irregular dietary habits, have led to nutritional deficiencies and associated health risks, thereby fueling global interest in functional foods and nutraceuticals due to their diverse health-promoting properties (538).

Functional foods are defined as either: (1) whole food ingredients or specific components used for targeted disease prevention and management (539), or (2) conventional foods enhanced with bioactive compounds, such as anthocyanin-enriched purple potatoes or carotenoid-fortified golden varieties (540). These specially formulated foods provide health benefits that extend beyond basic nutrition, including the reduction of chronic disease risk, while retaining the appearance, taste, and convenience of conventional dietary items (541).

Plant-based functional foods hold significant value due to their provision of essential nutrients, antioxidants, and bioactive compounds, which play a pivotal role in promoting and maintaining human health (5, 542, 543). The growing recognition of the disease-preventive potential of plant-based functional foods has stimulated extensive research into their immunomodulatory properties, driving increased consumer interest in adopting such diets for immune enhancement (55).

Scientific investigations have identified numerous bioactive compounds in these foods capable of regulating blood glucose levels (544). The active compounds can be divided into six main groups: flavonoids, steroidal saponins, polysaccharides, alkaloids, polyphenols, and other phytochemicals (544). Research highlights that polysaccharides from *Ganoderma* species enhance immune function by stimulating lymphocytes and myeloid cells to combat tumor development (545). Similarly, milkvetch (*Astragalus*), and ginseng (*Panax*) exhibit potent immunostimulatory effects (546). The key components responsible for strengthening immunity are polysaccharides, saponins, flavonoids, and alkaloids (547).

Natural polysaccharides, carbohydrate polymers formed by glycosidic bonds, exhibit multiple biological activities, including anticancer, immunomodulatory, and anti-inflammatory effects (128). Their low toxicity and minimal side effects make them attractive for immunity enhancement (548). These compounds have demonstrated the ability to concurrently stimulate innate immune responses and antigen-specific immunity, rendering them promising adjuvant candidates (548).

Emerging research confirms their immunostimulatory potential in both *in vitro* and *in vivo* systems, as evidenced by enhanced development of immune organs and increased secretion of immunomodulatory factors (549). Saponins, comprising triterpenoid or steroid aglycones, are increasingly recognized for their health-promoting properties in functional foods (550). Numerous studies have demonstrated the anti-tumor and immunomodulatory potential of plant-derived saponins (52), with pharmacological research confirming their immune-enhancing capabilities (551, 552).

Flavonoids, a major class of plant secondary metabolites, represent key bioactive components in functional foods (553, 554) and have attracted significant research attention for their immunostimulatory effects. Alkaloids are nitrogen-containing phytochemicals distinct from other nitrogenous compounds like proteins and amino acids (555), exhibit complex structures and diverse biological activities contributing to immune modulation (554). These compounds enhance immune function primarily by modulating the proliferation of thymic and splenic lymphocytes and regulating cytokine secretion (547). Other bioactive substances, such as terpenoids, essential oils, and organic acids, have shown notable potential in stimulating immune responses (547).

## 10 Food applications

The growing emphasis on healthy, sustainable nutrition has accelerated the utilization of plant-derived byproducts as sources of bioactive compounds (556). These substances serve multiple functional roles in food systems, including texture modification, antioxidant enhancement, antimicrobial protection, natural pigmentation, and nutritional fortification (557). Numerous studies demonstrate successful incorporation of byproduct-sourced bioactive compounds into diverse food matrices, enhancing both nutritional and functional properties in products such as yogurt (558), dry frozen fish (559), bread (560), petit Suisse cheese (561), beef patties (562), and cured sausage (563).

In food applications, the antioxidant capacity of these extracts correlates strongly with their total phenolic content, measurable through various analytical methods (558, 563, 564). Turgut et al. (564) fortified beef meatballs with pomegranate peel extract at a concentration of 10 g/kg (1,000 mg/100 g) and demonstrated that this addition significantly reduced lipid oxidation during refrigerated storage at 4°C, over 8 days. The 1% extract treatment showed comparable efficacy to synthetic butylated hydroxytoluene (BHT), reducing thiobarbituric acid-reactive substances by 53 and 50%, respectively, compared with the control (564).

Similarly, Choe et al. (565) reported that persimmon peel extract, applied at a concentration of 200 mg/100 g, effectively inhibited lipid oxidation in ground pork during a 12-day storage period at 3°C. Both extract and BHT treatments significantly lowered peroxide values (43 and 34% reduction, respectively) and conjugated diene formation compared to untreated samples, with the natural extract showing superior antioxidant performance (565). In another study, Ergezer and Serdaroğlu (566) investigated the antioxidant effects of artichoke byproduct extract (27 mg/100 g) compared to BHT in beef patties. The natural extract significantly outperformed synthetic BHT in inhibiting lipid and protein oxidation during storage, exhibiting a 42% higher total phenolic content and a 114% greater (2,2-diphenyl-1-picrylhydrazyl) DPPH radical scavenging capacity than the controls. In comparison, BHT showed minimal improvements, 4 and 9%, respectively (566).

Researchers attributed these superior results to the high phenolic content and antioxidant potential of the artichoke byproducts. Andres et al. (567) demonstrated that lamb patties supplemented with grape, olive, tomato, and pomegranate pomace extracts exhibited a 10%−21% reduction in mesophilic bacterial counts after 7 days of refrigerated storage compared to control samples (567). Comparable antimicrobial effects were reported in (1) pomegranate peel-fortified beef sausages and (2) shrimp treated with fruit byproduct-derived marinades (568, 569).

Nishad et al. (570) reported that meatballs formulated with nutmeg and citrus peel extracts (100 mg/100 g) exhibited significant antioxidant activity, effectively inhibiting both lipid and protein oxidation. Additionally, these formulations enhanced key sensory attributes, including color, aroma, flavor, and overall acceptability, throughout storage, compared to the control samples (570). Complementary studies demonstrated that chicken meat wafers incorporated with apple peel (2.5% w/w) or banana peel (2% w/w) exhibited similarly enhanced sensory characteristics, including improved flavor, texture, and overall acceptability (571).

According to Abid et al. (572), tomato pomace extracts were added to butter to evaluate their antioxidant effects. The supplemented butter containing 40 mg of extract per 100 g exhibited the lowest peroxide formation during storage, likely attributable to the high concentrations of lycopene and phenolic compounds in the extract. As reported by Bertolino et al. (573), the incorporation of hazelnut skin powder into yogurt increased its DPPH radical scavenging capacity and total phenolic content by 96 and 31%, respectively, compared to the control (573). Conversely, the growth of probiotics was enhanced when yogurts containing powdered pineapple peel were refrigerated at 4°C (574). Fresh orange juice fortified with banana peel extract (500 mg/100 ml) exhibited significantly enhanced antioxidant activity compared to the unfortified control, demonstrating approximately 21 and 150% higher scavenging capacity in the DPPH and ferric reducing antioxidant power (FRAP) assays, respectively (575).

In a recent study, Zaky et al. (576) investigated pasta formulations incorporating sunflower meal protein isolate (SMPI) at varying concentrations (3%, 6%, 9%). Their results demonstrated significant nutritional improvement in all supplemented samples, with optimal consumer acceptance at the 3%−6% SMPI inclusion levels (576). In complementary research, Kampuse et al. (577) reported that wheat bread enriched with pumpkin pomace powder (5.5-g/100-g dough) showed a 13-fold increase in carotenoid content compared to conventional formulations.

Numerous plant-based sources have demonstrated their capacity to enhance the total phenolic content and antioxidant efficiency in baked goods, including grape pomace (578, 579), plantain peel (580), mango peel (581), potato peel (582), raspberry and cranberry pomaces (583), beetroot pomace (584), apple pomace (585), rosehip, blackcurrant, and elderberry pomaces (586).

Table 4 illustrates the activities, mechanisms, and advantages associated with the use of plant-derived bioactive compounds in the food sector.

**Table 4:** Application of plant-derived bioactive compounds in the food industry: functions, mechanisms, and benefits.

**Plant extract (bioactive compounds)**	**Product application**	**Primary function(s)/benefit(s)**	**References**
Camu-camu (*Myrciaria dubia*) seed	Yogurt	Enhances the ability of food products to neutralize free radicals, which can help prevent spoilage and improve health benefits by reducing oxidative stress	(558)
Pomegranate and grape seeds	Minced fish muscle	Prevents or slows down the degradation of fats and oils in food, preserving flavor, texture, and nutritional quality	(559)
Grape seed	Bread	Boosts the antioxidant properties of the product while maintaining or improving its color, which is important for consumer acceptance	(560)
	Petit Suisse cheese	Improved antioxidant capacity (73%) with favorable sensory acceptability; significantly increases the product's antioxidant levels without compromising taste, texture, or overall sensory appeal	(561)
	Dry-cured sausage “chorizo”	Enhances the taste, aroma, and texture of the product while also reducing the breakdown of lipids	(563)
Pomegranate peel	Beef meatballs	Prevents the degradation of both proteins and fats, which helps maintain nutritional value and shelf life	(564)
Persimmon peel	Ground pork meat	Lowers the rate at which proteins and fats break down, preserving product quality and extending shelf life	(565)
Pomegranate peel	Beef sausages and white shrimp	Enhances the product's ability to inhibit the growth of harmful microorganisms, contributing to food safety and preservation	(568)
Nutmeg and citrus peel	Meatballs	Improves sensory evaluations; retarded lipid and protein oxidation. Leads to better taste, texture, and aroma, while also slowing the breakdown of proteins and fats	(569)
Apple and banana peels	Chicken meat wafers	Enhances the overall taste, aroma, and texture, making the product more appealing to consumers	(570)
Pineapple peel	Yogurt	Helps maintain or increase the number of beneficial probiotic bacteria in the product, supporting gut health	(574)
Banana peel	Fresh orange juice	Improves sensory evaluations and increased antioxidant activity. Results in better taste and texture while boosting the product's ability to fight oxidative damage	(575)
Nendran peel	Cookies	Adds fiber with antioxidant properties, which can improve digestive health and provide additional protection against oxidative stress	(580)
Potato peel	Biscuits	Reduces the formation of harmful compounds resulting from fat oxidation, thus preserving product quality	(581)
Mango peel	Biscuits	Improves the capacity to inhibit lipid peroxidation, and the extract can be used as a food ingredient.	(582)
Red beetroot pomace	Biscuits	Boosts the product's antioxidant content and overall nutritional value	(584)
Apple pomace	Rice-based cracker	Enhances taste, aroma, and texture while also increasing the product's antioxidant capacity	(585)
Blackcurrant, rosehip, and elderberry pomaces	Cookies	Improves the overall sensory experience and increases the ability to neutralize free radicals	(586)
Artichoke by-products	Beef patties	Reduces the breakdown of proteins and the amount of fats, which can be beneficial for health and product stability	(566)
Grape, olive, tomato, and pomegranate pomace	Lamb patties	Decreases the number of microorganisms that thrive at moderate temperatures, improving food safety and extending shelf life	(566)
Tomato pomace	Sauces, ketchup, Butter	Acts as a natural pigment and potent antioxidant; effectively quenches singlet oxygen, thereby significantly decreasing lipid oxidation and enhancing product shelf life	(572)
Pumpkin pomace	Wheat bread	Boosts carotenoid content in wheat bread, contributing to improved nutritional quality and delivering superior sensory attributes such as color, flavor, and texture	(577)
Grape pomace	Bread	Elevates antioxidant activity in breads, while also improving sensory properties such as taste and aroma, making products healthier and more appealing to consumers	(578)
	Biscuits	Reduces lipid peroxidation in biscuits, protecting fats from oxidative damage and helping maintain product freshness and quality over time	(579)
Hull, bur, and leaf chestnut	Beef patties	Decreases lipid oxidation in beef patties, helping preserve flavor and nutritional value, with no adverse effect on consumer sensory acceptance	(562)
Turmeric (curcumin)	Bakery products, dairy	Functions as a potent anti-inflammatory and natural coloring agent in bakery and dairy products; modulates the NF-κB pathway, contributing to health benefits and visual appeal	(559)
Hazelnut skins	Yogurt	Increases antioxidant activity in yogurt, enhancing its health-promoting properties and potentially extending shelf life	(573)
Sunflower meal protein isolate	Pasta	Improves the nutritional value of pasta by increasing protein content and contributing to a more balanced amino acid profile	(576)
Green tea (catechins)	Functional beverages	Provides strong antioxidant activity in functional beverages; efficiently scavenges free radicals and extends the product's shelf life, supporting consumer health	(674)
Beetroot (betalains)	Colorants in dairy and confectionery	Serves as a natural red colorant for dairy and confectionery products; enhances nitric oxide production, which may support cardiovascular health	(675)
Soybean (isoflavones)	Meat substitutes, soy milk	Hormonal balance and bone health bind estrogen receptors	(676)

## 11 Economic cost evaluations

A comparative analysis between conventional and contemporary extraction techniques is essential; however, limited research has comprehensively addressed this topic. Essien et al. (587) conducted an economic assessment of bioactive compound extraction from kanuka leaves using ethanol and subcritical water methods. Their analysis revealed that SWE was more cost-effective, with a manufacturing cost of NZ$4.49 million and a unit cost of NZ$2.14/kg, compared to ethanol extraction, which incurred NZ$4.7 million in manufacturing costs and a unit cost of NZ$5.57/kg (587).

Similarly, Lopeda-Correa et al. (588) reported that UAE (US$3.86/flask) was more economical than Soxhlet extraction (US$5.80/flask) for recovering polyphenols from *Adenaria floribunda* stems. Together, these studies highlight the greater cost-effectiveness of modern extraction techniques (588).

## 12 Challenges and limitations

The growing demands of global food production necessitate the development of improved extraction methods to yield high-quality bioactive compounds for industrial use. Although conventional extraction methods remain widely used, their environmental limitations, such as high energy consumption, reliance on toxic solvents, and thermal degradation of heat-sensitive compounds, pose significant challenges to sustainable production practices (589).

In contrast, emerging green extraction technologies offer faster, more selective, and eco-friendly alternatives with better temperature control, though further validation is needed for large-scale industrial adoption (590). Despite their potential, many emerging extraction technologies face prohibitive costs and scalability challenges, as industrial-scale equipment often remains in prototype stages or requires custom designs (590). However, the food manufacturing sector anticipates advancements to address these limitations. Meanwhile, growing consumer health consciousness has driven demand for nutrient-dense, plant-derived foods, increasing the availability of purified plant extracts for industrial use (591). These extracts, often concentrated or refined into premium nutraceuticals and dietary supplements, offer demonstrated health benefits, including LDL cholesterol reduction, blood pressure management, atherosclerosis mitigation, cognitive enhancement, oxidative stress reduction, and anticancer properties (591).

Commercial grape seed extracts, such as ORAC-15 M™ (Ethical Naturals, Inc., Novato, CA, USA), are standardized to contain 80% polyphenols and exhibit an oxygen radical absorbance capacity (ORAC) of 15,000, and they are marketed for their potential in mitigating oxidative stress. Applied Food Science Inc., Kerrville, Texas, USA, provides a range of premium plant-based extracts, such as GCE-50™ green coffee extract standardized to 50% chlorogenic acids, PureGinger™ organic powder with 2% gingerol content, CoffeeNectar™ cascara fruit extract, and PurTea™ concentrated green tea caffeine (592).

While these phenolic-rich extracts offer superior antioxidant activity, higher concentrations may impart bitterness, aftertaste, or color changes in final products (593). Despite their potential, widespread adoption faces challenges, including limited human clinical trials and animal studies, which hinder assessments of bioavailability and market validation. Further research is needed to evaluate *in vivo* efficacy, safety (including toxicity, cytotoxicity, and allergenicity), and regulatory standards to ensure consumer protection against misleading claims (35).

The inclusion of plant bioactive compounds in functional foods faces a complex landscape of global regulatory challenges. Key obstacles include divergent regional requirements, ingredient safety concerns, labeling constraints, health claim substantiation, and the lack of harmonized international standards (594, 595). Different regions, including the EU, the USA, Asia, and Canada, define, regulate, and categorize functional foods and bioactive ingredients in varying ways. For example, the EU uses a negative list for prohibited botanicals and requires pre-market approval for novel foods, whereas Japan has specific categories, such as Foods for Specified Health Use (FOSHU), that demand government certification and scientifically substantiated claims (595). In the USA, botanical ingredients must comply with GRAS notifications or new dietary ingredient notifications for supplements, but regulatory clarity for functional foods is limited (595, 596).

Authorities typically require extensive safety evidence, including toxicological data and proof of the absence of contaminants, for plant-based ingredients. The variability introduced by different plant species, growing regions, and processing methods makes standardization challenging (594). Establishing maximum daily intake limits and monitoring for potential adverse effects (including food-drug interactions) are mandated in regions like Canada and South Korea, adding to the regulatory burden (594).

Labeling requirements for functional foods containing botanicals vary greatly. In Canada and South Korea, detailed labeling of bioactive content, function, and warnings is compulsory (595). In the EU and Japan, only claims approved after rigorous scientific assessment may be placed on products. Misleading or unsubstantiated health claims are a major concern, with severe penalties and product recalls possible if regulatory standards are breached (594, 595). The absence of global uniform guidelines means companies must navigate fragmented legal frameworks. This often results in increased costs, delays, and product reformulation for cross-border trade. Regulatory requirements for documentation, scientific substantiation, and language of claims further complicate market entry for functional foods with plant bioactive compounds (597).

Regulatory approval for new plant-derived bioactive ingredients, such as those obtained by novel extraction or biotechnological processes, often requires case-by-case risk assessment, further delaying product launches (595). Integrating traditional medicinal plants into mainstream foods also presents a challenge, as authorities scrutinize both the history of use and contemporary scientific evidence (594, 595).

## 13 Gaps, future studies, and conclusion

This comprehensive review addresses critical knowledge gaps in bioactive plant compounds research by identifying three primary areas requiring immediate scientific attention: regulatory harmonization challenges, extraction technology limitations, and commercial scalability barriers. The growing consumer preference for functional foods, driven by rising chronic diseases including obesity, diabetes, and cardiovascular disorders, has created an urgent need for evidence-based solutions that current literature fails to address (598, 599) adequately.

The most significant gap lies in the absence of standardized global regulatory frameworks for functional foods containing bioactive plant compounds. While the European Commission's CLYMBOL Project represents the first multinational effort to examine the influence of health claims on consumer behavior, comprehensive international harmonization remains elusive (599, 600). Current regulations vary dramatically across jurisdictions—from Japan's FOSHU certification system to the EU's negative lists of botanicals and the US GRAS notification requirements—creating substantial barriers to global commercialization. This regulatory fragmentation forces manufacturers to navigate complex, region-specific approval processes, significantly increasing development costs and time-to-market delays (597, 600).

Extraction technology limitations present another critical research gap, particularly in terms of industrial scalability and environmental sustainability. While advanced techniques such as SFE, MAE, and UAE demonstrate superior efficiency in laboratory settings, their commercial implementation faces substantial challenges, including high capital investments, concerns over energy consumption, and difficulties with process standardization. Recent studies indicate that up to 80% of bioactive compound research remains confined to laboratory scales, with limited pilot-scale validation and virtually no large-scale industrial applications (601, 602).

The bioavailability and delivery system challenges represent a third significant gap that this review systematically addresses. Current research predominantly focuses on compound extraction and characterization, while neglecting critical aspects such as bioavailability enhancement, targeted delivery, and stability preservation during food processing and storage. The variability in individual responses due to factors such as age, gender, metabolism, and lifestyle creates additional complexity that the existing literature inadequately addresses (601, 602).

Future research priorities must focus on developing hybrid extraction technologies that combine multiple approaches to optimize efficiency while minimizing environmental impact. The integration of AI and machine learning for parameter optimization represents a particularly promising direction that could address current scalability challenges. Additionally, establishing standardized analytical methods for characterizing bioactive plant compounds and assessing their bioactivity is essential for ensuring reproducibility and facilitating regulatory approval processes (601, 602).

The current review highlights sustainable biorefinery concepts, focusing on the essential requirements for waste valorization and the application of circular economy principles in the synthesis of bioactive compounds. This approach not only reduces environmental impact but also enhances economic viability by maximizing raw material utilization and generating multiple revenue streams from a single extraction process.

Ultimately, the pressing need for international regulatory harmonization necessitates collaborative efforts among global food safety authorities to develop unified standards for functional foods, thereby facilitating seamless cross-border trade while ensuring consumer protection and product safety.

## References

[1] Parvatiyar A Sheth JN. Confronting the deep problem of consumption: why individual responsibility for mindful consumption matters. J Consum Aff. (2023) 57:785–820. doi: 10.1111/joca.12534

[2] Mandelli L Milaneschi Y Hiles S Serretti A Penninx BW. Unhealthy lifestyle impacts on biological systems involved in stress response: hypothalamic–pituitary–adrenal axis, inflammation and autonomous nervous system. Int Clin Psychopharmacol. (2023) 38:127–35. doi: 10.1097/YIC.0000000000000437 36730700 PMC10063190

[3] AlAli M Alqubaisy M Aljaafari MN AlAli AO Baqais L Molouki A . Nutraceuticals: transformation of conventional foods into health promoters/disease preventers and safety considerations. Molecules. (2021) 26:2540. doi: 10.3390/molecules26092540 33925346 PMC8123587

[4] Jedrusek-Golińska A Górecka D Buchowski M Wieczorowska-Tobis K Gramza-Michałowska A Szymandera-Buszka K. Recent progress in the use of functional foods for older adults: a narrative review. Compr Rev Food Sci Food Saf. (2020) 19:835–56. doi: 10.1111/1541-4337.12530 33325174

[5] Mondal S Soumya NPP Mini S Sivan SK. Bioactive compounds in functional food and their role as therapeutics. Bioact Comp Health Dis. (2021) 4:24–39. doi: 10.31989/bchd.v4i3.786

[6] Martirosyan D von Brugger J Bialow S. Functional food science: differences and similarities with food science. Funct Foods Health Dis. (2021) 11:408–30. doi: 10.31989/ffhd.v11i9.831

[7] Essa MM Bishir M Bhat A Chidambaram SB Al-Balushi B Hamdan H . Functional foods and their impact on health. J Food Sci Technol. (2023) 60:820–34. doi: 10.1007/s13197-021-05193-3 36908338 PMC9998796

[8] Vlaicu PA Untea AE Varzaru I Saracila M Oancea AG. Designing nutrition for health—incorporating dietary by-products into poultry feeds to create functional foods with insights into health benefits, risks, bioactive compounds, food component functionality and safety regulations. Foods. (2023) 12:4001. doi: 10.3390/foods12214001 37959120 PMC10650119

[9] Gupta A Sanwal N Bareen MA Barua S Sharma N Olatunji OJ . Trends in functional beverages: functional ingredients, processing technologies, stability, health benefits, and consumer perspective. Food Res Int. (2023) 170:113046. doi: 10.1016/j.foodres.2023.113046 37316029

[10] Rane BR Amkar AJ Patil VS Vidhate PK Patil AR. Opportunities and challenges in the development of functional foods and nutraceuticals. In:Rao TJM Kesharwani RK Keservani RK Sharma AK, editors. Formulations, Regulations, and Challenges of Nutraceuticals. New York, NY: Apple Academic Press (2024). p. 227–54. doi: 10.1201/9781003412496-14

[11] Herdiana Y. Functional food in relation to gastroesophageal reflux disease (GERD). Nutrients. (2023) 15:3583. doi: 10.3390/nu15163583 37630773 PMC10458865

[12] Indriyani NN Anshori JA Permadi N Nurjanah S. Julaeha E. Bioactive components and their activities from different parts of *Citrus aurantifolia* (Christm) Swingle for food development. Foods. (2023) 12:2036. doi: 10.3390/foods12102036 37238855 PMC10217416

[13] Tufail T Fatima S Bader Ul Ain H Ikram A Noreen S Rebezov M . Role of phytonutrients in the prevention and treatment of chronic diseases: a concrete review. ACS Omega. (2025) 10:12724–55. doi: 10.1021/acsomega.4c02927 40224418 PMC11983219

[14] Rawat M Varshney A Rai M Chikara A Pohty AL Joshi A . A comprehensive review on nutraceutical potential of underutilized cereals and cereal-based products. J Agric Food Res. (2023) 12:100619. doi: 10.1016/j.jafr.2023.100619

[15] El-Saadony MT Saad AM Mohammed DM Korma SA Alshahrani MY Ahmed AE . Medicinal plants: bioactive compounds, biological activities, combating multidrug-resistant microorganisms, and human health benefits - A comprehensive review. Front Immunol. (2025) 16:1491777. doi: 10.3389/fimmu.2025.1491777 40375989 PMC12079674

[16] Onuh JO Pathak YV. Introduction to food bioactive phytochemicals. In:Onuh JO Pathak YV, editors. Plant Food Phytochemicals and Bioactive Compounds in Nutrition and Health. Boca Raton, FL: CRC Press (2024). p. 1–14. doi: 10.1201/9781003340201-1

[17] Islam MS Wang H Admassu H Sulieman AA Wei FA. Health benefits of bioactive peptides produced from muscle proteins: antioxidant, anti-cancer, and anti-diabetic activities. Process Biochem. (2022) 116:116–25. doi: 10.1016/j.procbio.2022.03.007

[18] Ambroselli D Masciulli F Romano E Catanzaro G Besharat ZM Massari MC . New advances in metabolic syndrome, from prevention to treatment: the role of diet and food. Nutrients. (2023) 15:640. doi: 10.3390/nu15030640 36771347 PMC9921449

[19] Banwo K Olojede AO Adesulu-Dahunsi AT Verma DK Thakur M Tripathy S . Functional importance of bioactive compounds of foods with potential health benefits: a review on recent trends. Food Biosci. (2021) 43:101320. doi: 10.1016/j.fbio.2021.101320

[20] Rudrapal M Rakshit G Singh RP Garse S Khan J Chakraborty S. Dietary polyphenols: review on chemistry/sources, bioavailability/metabolism, antioxidant effects, and their role in disease management. Antioxidants. (2024) 13:429. doi: 10.3390/antiox13040429 38671877 PMC11047380

[21] Manzoor A Yousuf B Pandith JA Ahmad S. Plant-derived active substances incorporated as antioxidant, antibacterial or antifungal components in coatings/films for food packaging applications. Food Biosci. (2023) 53:102717. doi: 10.1016/j.fbio.2023.102717

[22] Sadh PK Duhan S Duhan JS. Agro-industrial wastes and their utilization using solid state fermentation: a review. Bioresour Bioprocess. (2018) 5:1–15. doi: 10.1186/s40643-017-0187-z

[23] Kumari B Tiwari BK Hossain MB Brunton NP Rai DK. Recent advances on application of ultrasound and pulsed electric field technologies in the extraction of bioactives from agro-industrial by-products. Food Bioprod Process. (2018) 11:223–41. doi: 10.1007/s11947-017-1961-9

[24] de los Ángeles Fernández M Espino M Gomez FJ Silva MF. Novel approaches mediated by tailor-made green solvents for the extraction of phenolic compounds from agro-food industrial by-products. Food Chem. (2018) 239:671–8. doi: 10.1016/j.foodchem.2017.06.150 28873620

[25] Ng HS Kee PE Yim HS Chen PT Wei YH Lan JCW. Recent advances on the sustainable approaches for conversion and reutilization of food wastes to valuable bioproducts. Bioresour Technol. (2020) 302:122889. doi: 10.1016/j.biortech.2020.122889 32033841

[26] Toschi F Sega M. Flowing Matter. Cham: Springer Nature (2019) p. 309. doi: 10.1007/978-3-030-23370-9

[27] Varzakas T Zakynthinos G Verpoort F. Plant food residues as a source of nutraceuticals and functional foods. Foods. (2016) 5:88. doi: 10.3390/foods5040088 28231183 PMC5302437

[28] Coman V Teleky BE Mitrea L Martău GA Szabo K Călinoiu LF . Bioactive potential of fruit and vegetable wastes. Adv Food Nutr Res. (2020) 91:157–225. doi: 10.1016/bs.afnr.2019.07.001 32035596

[29] Lopes FC Ligabue-Braun R. Agro-industrial residues: eco-friendly and inexpensive substrates for microbial pigments production. Front Sustain Food Syst. (2021) 5:589414. doi: 10.3389/fsufs.2021.589414

[30] Lemes AC Egea MB. Oliveira Filho JGd, Gautério GV, Ribeiro BD, Coelho MAZ. Biological approaches for extraction of bioactive compounds from agro-industrial by-products: a review. Front Bioeng Biotechnol. (2022) 9:802543. doi: 10.3389/fbioe.2021.802543 35155407 PMC8829320

[31] Sobhy R Zou X Morsy OM Zaky AA Khalifa I. Date seed polyphenol pills as renewable raw materials showed anti-obesity effects with high digestible antioxidants in 3T3-L1 cells. Appl Sci. (2023) 13:12533. doi: 10.3390/app132212533

[32] Zaky AA Chen Z Qin M Wang M Jia Y. Assessment of antioxidant activity, amino acids, phenolic acids and functional attributes in defatted rice bran and rice bran protein concentrate. Prog Nutr. (2020) 22:e2020069. doi: 10.23751/pn.v22i4.8971

[33] Rifna EJ Misra NN Dwivedi M. Recent advances in extraction technologies for recovery of bioactive compounds derived from fruit and vegetable waste peels: a review. Crit Rev Food Sci Nutr. (2023) 63:719–52. doi: 10.1080/10408398.2021.1952923 34309440

[34] Ünlü AE. Green and non-conventional extraction of bioactive compounds from olive leaves: screening of novel natural deep eutectic solvents and investigation of process parameters. Waste Biomass Valor. (2021) 12:5329–46. doi: 10.1007/s12649-021-01411-3 33727990 PMC7953197

[35] Zaky AA Simal-Gandara J Eun JB Shim JH Abd El-Aty AM. Bioactivities, applications, safety, and health benefits of bioactive peptides from food and by-products: a review. Front Nutr. (2022) 8:815640. doi: 10.3389/fnut.2021.815640 35127796 PMC8810531

[36] Jha AK Sit N. Extraction of bioactive compounds from plant materials using combination of various novel methods: a review. Trends Food Sci Technol. (2022) 119:579–91. doi: 10.1016/j.tifs.2021.11.019

[37] Ben-Othman S Jõudu I Bhat R. Bioactives from agri-food wastes: present insights and future challenges. Molecules. (2020) 25:510. doi: 10.3390/molecules25030510 31991658 PMC7037811

[38] Deo S Sakhale BK. A review on potential of bioactive compounds obtained from processing waste of various fruits and vegetables. Int J Pure Appl Biosci. (2018) 6:680–6. doi: 10.18782/2320-7051.6742

[39] Jeon YA Chung SW Kim SC Lee YJ. Comprehensive assessment of antioxidant and anti-inflammatory properties of papaya extracts. Foods. (2022) 11:3211. doi: 10.3390/foods11203211 37430960 PMC9601897

[40] Suleria HAR Barrow CJ Dunshea FR. Screening and characterization of phenolic compounds and their antioxidant capacity in different fruit peels. Foods. (2020) 9:1206. doi: 10.3390/foods9091206 32882848 PMC7556026

[41] Sultana B Hussain Z Asif M. Munir A. Investigation on the antioxidant activity of leaves, peels, stems, bark, and kernel of mango (*Mangifera indica* L.). J Food Sci. (2012) 77:C849–52. doi: 10.1111/j.1750-3841.2012.02807.x 22860576

[42] Wolfe K Wu X Liu RH. Antioxidant activity of apple peels. J Agric Food Chem. (2003) 51:609–14. doi: 10.1021/jf020782a 12537430

[43] Matsuo Y Miura LA Araki T Yoshie-Stark Y. Proximate composition and profiles of free amino acids, fatty acids, minerals, and aroma compounds in *Citrus natsudaidai* peel. Food Chem. (2019) 279:356–63. doi: 10.1016/j.foodchem.2018.11.146 30611501

[44] Zou Z Xi W Hu Y Nie C Zhou Z. Antioxidant activity of citrus fruits. Food Chem. (2016) 196:885–96. doi: 10.1016/j.foodchem.2015.09.072 26593569

[45] Rajasree RS Sibi PI Francis F William H. Phytochemicals of Cucurbitaceae family—A review. Int J Pharmacogn Phytochem Res. (2016) 8:113–23.

[46] Ravichandran K Saw NMMT Mohdaly AA Gabr AM Kastell A Riedel H . Impact of processing of red beet on betalain content and antioxidant activity. Food Res Int. (2013) 50:670–5. doi: 10.1016/j.foodres.2011.07.002

[47] Cartea ME Francisco M Soengas P Velasco P. Phenolic compounds in *Brassica* vegetables. Molecules. (2010) 16:251–80. doi: 10.3390/molecules16010251 21193847 PMC6259264

[48] Al-Weshahy A El-Nokety M Bakhete M Rao V. Effect of storage on antioxidant activity of freeze-dried potato peels. Food Res Int. (2013) 50:507–12. doi: 10.1016/j.foodres.2010.12.014

[49] Sahin S Samli R Tan ASB Barba FJ Chemat F Cravotto G . Solvent-free microwave-assisted extraction of polyphenols from olive tree leaves: antioxidant and antimicrobial properties. Molecules. (2017) 22:1056. doi: 10.3390/molecules22071056 28672807 PMC6152306

[50] Talhaoui N Gómez-Caravaca AM Roldan C Leon L De la Rosa R Fernandez-Gutierrez A Segura-Carretero A. Chemometric analysis for the evaluation of phenolic patterns in olive leaves from six cultivars at different growth stages. J Agric Food Chem. (2015) 63:1722–9. doi: 10.1021/jf5058205 25613562

[51] Liu Y Ren C Zhan R Cao Y Ren Y Zou L . Exploring the potential of plant-derived exosome-like nanovesicle as functional food components for human health: a review. Foods. (2024) 13:712. doi: 10.3390/foods13050712 38472825 PMC10930737

[52] Gong X Li X Xia Y Xu J Li Q Zhang C . Effects of phytochemicals from plant-based functional foods on hyperlipidemia and their underpinning mechanisms. Trends Food Sci Technol. (2020) 103:304–20. doi: 10.1016/j.tifs.2020.07.026

[53] Mohamad NE Abu N Yeap SK Lim KL Romli MF Sharifuddin SA . Apoptosis and metastasis inhibitory potential of pineapple vinegar against mouse mammary gland cells *in vitro* and *in vivo*. Nutr Metab. (2019) 16:49. doi: 10.1186/s12986-019-0380-5 31372176 PMC6660685

[54] Khairnar SJ Rudrapal M Ahire ED Jagtap MR Kshirsagar SJ. Overview of functional foods. In:Keservani RK Ahire ED, editors. Applications of Functional Foods in Disease Prevention. New York, NY: Apple Academic Press (2024). p. 1–31. doi: 10.1201/9781003395737-1

[55] Davoodvandi A Sahebnasagh R Mardanshah O Asemi Z Nejati M Shahrzad MK . Medicinal plants as natural polarizers of macrophages: phytochemicals and pharmacological effects. Curr Pharm Des. (2019) 25:3225–38. doi: 10.2174/1381612825666190829154934 31465276

[56] Owushi JN Asanga DE. Assessment of human health improved fruits and vegetables: the benefits for growing children. Peerian J. (2024) 27:117–29.

[57] Nayak SN Aravind B Malavalli SS Sukanth BS Poornima R Bharati P . Omics technologies to enhance plant based functional foods: an overview. Front Genet. (2021) 12:742095. doi: 10.3389/fgene.2021.742095 34858472 PMC8631721

[58] Ezeorba TPC Chukwudozie KI Ezema CA Anaduaka EG Nweze EJ Okeke ES. Potentials for health and therapeutic benefits of garlic essential oils: recent findings and future prospects. Pharmacol Res Mod Chin Med. (2022) 3:100075. doi: 10.1016/j.prmcm.2022.100075

[59] Ezzat A Abdelhamid AO El Awady MK Abd El Azeem AS Mohammed DM. The biochemical effects of nano tamoxifen and some bioactive components in experimental breast cancer. Biomed Pharmacother. (2017) 95:571–6. doi: 10.1016/j.biopha.2017.08.099 28869895

[60] Fang HY Chen SB Guo DJ Pan SY Yu ZL. Proteomic identification of differentially expressed proteins in curcumin-treated MCF-7 cells. Phytomedicine. (2011) 18:697–703. doi: 10.1016/j.phymed.2010.11.012 21239154

[61] Fuloria S Mehta J Chandel A Sekar M Rani NNIM Begum MY . comprehensive review on the therapeutic potential of *Curcuma longa* Linn. in relation to its major active constituent curcumin. Front Pharmacol. (2022) 13:820806. doi: 10.3389/fphar.2022.820806 35401176 PMC8990857

[62] Sowbhagya HB. Chemistry, technology, and nutraceutical functions of cumin (*Cuminum cyminum* L.): an overview. Crit Rev Food Sci Nutr. (2013) 53:1–10. doi: 10.1080/10408398.2010.500223 23035918

[63] Abdelmuhsin AA Sulieman AME Salih ZA Al-Azmi M Alanaizi NA Goniem AE . Clove (*Syzygium aromaticum*) pods: revealing their antioxidant potential via GC-MS analysis and computational insights. Pharmaceuticals. (2025) 18:504. doi: 10.3390/ph18040504 40283940 PMC12030067

[64] Lee K-G. Shibamoto T. Antioxidant property of aroma extract isolated from clove buds [*Syzygium aromaticum* (L) Merr et Perry]. Food Chem. (2001) 74:443–8. doi: 10.1016/S0308-8146(01)00161-3

[65] Ashokkumar K Murugan M Dhanya MK Pandian A. Warkentin TD. Phytochemistry and therapeutic potential of black pepper (*Piper nigrum* L.) essential oil and piperine: a review. Clin Phytosci. (2021) 7:52. doi: 10.1186/s40816-021-00292-2

[66] Zhang CR Dissanayake AA Kevseroğlu K Nair MG. Evaluation of coriander spice as a functional food by using *in vitro* bioassays. Food Chem. (2015) 167:24–9. doi: 10.1016/j.foodchem.2014.06.120 25148954

[67] Sabry MO Sedeek M Issa MY Elzalabani S. Plants effective in the control of hyperlipidemia and hypercholesterolemia: a review. Egypt J Chem. (2024) 67:33–41. doi: 10.21608/ejchem.2023.227181.8364

[68] Rizvi SA Einstein GP Tulp OL Sainvil F Branly R. Introduction to traditional medicine and their role in prevention and treatment of emerging and re-emerging diseases. Biomolecules. (2022) 12:1442. doi: 10.3390/biom12101442 36291651 PMC9599697

[69] Sun W Shahrajabian MH. Therapeutic potential of phenolic compounds in medicinal plants—natural health products for human health. Molecules. (2023) 28:1845. doi: 10.3390/molecules28041845 36838831 PMC9960276

[70] Granato D Zabetakis I Koidis A. Sustainability, nutrition, and scientific advances of functional foods under the new EU and global legislation initiatives. J Funct Foods. (2023) 109:105793. doi: 10.1016/j.jff.2023.105793

[71] Nieto G. How are medicinal plants useful when added to foods? Medicines. (2020) 7:58. doi: 10.3390/medicines7090058 32937755 PMC7555097

[72] Qiu K Wang S Duan F Sang Z Wei S Liu H . Rosemary: unrevealing an old aromatic crop as a new source of promising functional food additive—a review. Compr Rev Food Sci Food Saf. (2024) 23:e13273. doi: 10.1111/1541-4337.13273 38284599

[73] Nieto G Ros G. Castillo J. Antioxidant and antimicrobial properties of rosemary (*Rosmarinus officinalis* L.): a review. Medicines. (2018) 5:98. doi: 10.3390/medicines5030098 30181448 PMC6165352

[74] Shankar A Ali A Abdullah HM Balaji J Kaur J Saeed F . Nutritional composition, phytochemical profile, therapeutic potentials, and food applications of rosemary: a comprehensive review. J Food Compos Anal. (2024) 135:106688. doi: 10.1016/j.jfca.2024.106688

[75] Kaur R Gupta TB Bronlund J Kaur L. The potential of rosemary as a functional ingredient for meat products—A review. Food Rev Int. (2023) 39:2212–32. doi: 10.1080/87559129.2021.1950173

[76] Ezzaky Y Zanzan M Elmoslih A Msanda F Achemchem F. Impact of rosemary (*Rosmarinus officinalis*) and thyme (*Thymus satureioides*) essential oils on the physicochemical, microbiological, and sensory properties of Merguez sausage during the fermentation process. Malays J Microbiol. (2024) 20:720. doi: 10.21161/mjm.230358

[77] Pandit VA. Shelef LA. Sensitivity of Listeria monocytogenes to rosemary (*Rosmarinus officinalis* L.). Food Microbiol. (1994) 11:57–63. doi: 10.1006/fmic.1994.1008

[78] Fernandez-Lopez J Zhi N Aleson-Carbonell L Pérez-Alvarez JA Kuri V. Antioxidant and antibacterial activities of natural extracts: application in beef meatballs. Meat Sci. (2005) 69:371–80. doi: 10.1016/j.meatsci.2004.08.004 22062974

[79] Papadochristopoulos A Kerry JP Fegan N Burgess CM Duffy G. Potential use of selected natural anti-microbials to control *Listeria monocytogenes* in vacuum-packed beef burgers and their impact on quality attributes. Microorganisms. (2025) 13:910. doi: 10.3390/microorganisms13040910 40284746 PMC12029336

[80] Abdoon ASS Hegazy AM Abdel-Azeem AS Al-Atrash AM Mohammed DM. The protective effects of some herbs on mitigating HFD-induced obesity via enhancing biochemical indicators and fertility in female rats. Heliyon. (2024) 10:e26611. doi: 10.1016/j.heliyon.2024.e30249 38726161 PMC11078881

[81] Ortega-Ramirez LA Rodriguez-Garcia I Leyva JM Cruz-Valenzuela MR Silva-Espinoza BA Gonzalez-Aguilar GA . Potential of medicinal plants as antimicrobial and antioxidant agents in food industry: a hypothesis. J Food Sci. (2014) 79:R129–37. doi: 10.1111/1750-3841.12341 24446991

[82] Hou T Sana SS Li H Xing Y Nanda A Netala VR Zhang Z. Essential oils and its antibacterial, antifungal and anti-oxidant activity applications: a review. Food Biosci. (2022) 47:101716. doi: 10.1016/j.fbio.2022.101716

[83] Kumar S Bhushan B Wakchaure GC Dutta R Jat BS Meena KK . Unveiling the impact of heat stress on seed biochemical composition of major cereal crops: implications for crop resilience and nutritional value. Plant Stress. (2023) 9:100183. doi: 10.1016/j.stress.2023.100183

[84] Meghwal M Goyal MR. State-of-the-art Technologies in Food Science: Human Health, Emerging Issues and Specialty Topics. New York, NY: Apple Academic Press (2018). p. 396. doi: 10.1201/9781315165271

[85] Oluwole O Fernando WB Lumanlan J Ademuyiwa O Jayasena V. Role of phenolic acid, tannins, stilbenes, lignans and flavonoids in human health – a review. Int J Food Sci Technol. (2022) 57:6326–35. doi: 10.1111/ijfs.15936

[86] Keşa AL Pop CR Mudura E Salanţă LC Pasqualone A Dărab C . Strategies to improve the potential functionality of fruit-based fermented beverages. Plants. (2021) 10:2263. doi: 10.3390/plants10112263 34834623 PMC8623731

[87] Sun-Waterhouse D. The development of fruit-based functional foods targeting the health and wellness market: a review. Int J Food Sci Technol. (2011) 46:899–920. doi: 10.1111/j.1365-2621.2010.02499.x

[88] Yadav D Pal AK Singh SP Sati K. Phytochemicals in mango (*Mangifera indica*) parts and their bioactivities: a review. Crop Res. (2022) 57:79–95. doi: 10.31830/2454-1761.2022.012

[89] Yao P Gao Y Simal-Gandara J Farag MA Chen W Yao D. et al. Litchi (*Litchi chinensis* Sonn): a comprehensive review of phytochemistry, medicinal properties, and product development. Food Funct. (2021) 12:9527–48. doi: 10.1039/D1FO01148K 34664581

[90] Emanuele S Lauricella M Calvaruso G D'Anneo A Giuliano M. Litchi chinensis as a functional food and a source of antitumor compounds: an overview and a description of biochemical pathways. Nutrients. (2017) 9:992. doi: 10.3390/nu9090992 PMC5622752 28885570

[91] Çiftçi S. Suna GÜLEN. Functional components of peanuts (*Arachis hypogaea* L.) and health benefits: a review. Future Foods. (2022) 5:100140. doi: 10.1016/j.fufo.2022.100140

[92] Arya SS Salve AR Chauhan S. Peanuts as functional food: a review. J Food Sci Technol. (2016) 53:31–41. doi: 10.1007/s13197-015-2007-9 26787930 PMC4711439

[93] Ortiz C. Martirosyan D. Bioactive compounds in peanuts (*Arachis hypogaea* L.): a review of their anti-inflammatory and antioxidant effects. Agric Food Bioact Compd. (2024) 1:1–18. doi: 10.31989/afbc.v1i12.1525

[94] Cai W Zhuang H Wang X Fu X Chen S Yao L . Functional nutrients and jujube-based processed products in *Ziziphus jujuba*. Molecules. (2024) 29:3437. doi: 10.3390/molecules29143437 39065014 PMC11279998

[95] Deng Y Liu Y Zhang C Xie P Huang L. Characterization of enzymatic modified soluble dietary fiber from *Rhodomyrtus tomentosa* fruits: a potential ingredient in reducing AGEs accumulation. Food Bioproc Technol. (2023) 16:232–46. doi: 10.1007/s11947-022-02935-9

[96] Thielecke F Lecerf JM Nugent AP. Processing in the food chain: do cereals have to be processed to add value to the human diet? Nutr Res Rev. (2021) 34:159–73. doi: 10.1017/S0954422420000207 32854794

[97] Baniwal P Mehra R Kumar N Sharma S Kumar S. Cereals: functional constituents and its health benefits. Pharma Innov. (2021) 10:343–9. doi: 10.22271/tpi.2021.v10.i2e.5681

[98] Zaib S Hayat A Khan I. Nutritional and health benefits of cereals and grains. Curr Nutr Food Sci. (2024) 20:1205–21. doi: 10.2174/0115734013282127231220103115

[99] Guo H Wu H Sajid A Li Z. Whole grain cereals: the potential roles of functional components in human health. Crit Rev Food Sci Nutr. (2022) 62:8388–402. doi: 10.1080/10408398.2021.1928596 34014123

[100] Singla A Gupta OP Sagwal V Kumar A Patwa N Mohan N. Ankush, Kumar D, Vir O, Singh J, Kumar L. Beta-glucan as a soluble dietary fiber source: origins, biosynthesis, extraction, purification, structural characteristics, bioavailability, biofunctional attributes, industrial utilization, and global trade. Nutrients. (2024) 16:900. doi: 10.3390/nu16060900 38542811 PMC10975496

[101] Saikia D Deka SC. Cereals: from staple food to nutraceuticals. Int Food Res J. (2011) 18:21–30.

[102] Kumari K Kashyap P Chakrabarti P. Germination and probiotic fermentation: a way to enhance nutritional and biochemical properties of cereals and millets. Food Sci Biotechnol. (2024) 33:505–18. doi: 10.1007/s10068-023-01401-2 38274183 PMC10805689

[103] Charalampopoulos D Wang R Pandiella SS Webb C. Application of cereals and cereal components in functional foods: a review. Int J Food Microbiol. (2002) 79:131–41. doi: 10.1016/S0168-1605(02)00187-3 12382693

[104] Achi OK Asamudo NU. Cereal-based fermented foods of Africa as functional foods. In:Mérillon JM Ramawat KG, editors. Bioactive Molecules in Food. Reference Series in Phytochemistry. Cham: Springer (2019). p. 1527–58. doi: 10.1007/978-3-319-78030-6_31

[105] Bora P Ragaee S Marcone M. Characterisation of several types of millets as functional food ingredients. Int J Food Sci Nutr. (2019) 70:714–24. doi: 10.1080/09637486.2019.1570086 30969135

[106] Alija G Dauti M Havziu D Haxhiu Zaimi A Nuhii N Ibrahimi Q. Human health and importance of nutrition. Acta Med Balkan Int J Med Sci. (2024) 9:147–55. doi: 10.62792/ut.amb.v9.i17-18.p2563

[107] Blakeney M. Food Loss and Waste and Food Security. Cheltenham: Edward Elgar Publishing (2019). p. 26. doi: 10.4337/9781788975391

[108] Papastavropoulou K Proestos C. Vegetables as functional foods against cardiovascular diseases. In:Zabetakis I Tsoupras A Lordan R Ramji D, editors. Functional Foods and Their Implications for Health Promotion. Cambridge, MA: Academic Press (2023). p. 3–28. doi: 10.1016/B978-0-12-823811-0.00005-5

[109] Ali EA Mohammed DM Abd El Gawad F Orabi MA Gupta RK Srivastav PP. Valorization of food processing waste byproducts for essential oil production and their application in food system. Waste Manag Bull. (2025) 3:100200. doi: 10.1016/j.wmb.2025.100200

[110] Fotschki J Ogrodowczyk AM Wróblewska B Juśkiewicz J Side. streams of vegetable processing and its bioactive compounds support microbiota, intestine milieu, and immune system. Molecules. (2023) 28:4340. doi: 10.3390/molecules28114340 37298819 PMC10254940

[111] Jiménez Bolaño DC Insuasty D Rodríguez Macías JD Grande-Tovar CD. Potential use of tomato peel, a rich source of lycopene, for cancer treatment. Molecules. (2024) 29:3079. doi: 10.3390/molecules29133079 38999031 PMC11243680

[112] Di Mascio P Kaiser S Sies H. Lycopene as the most efficient biological carotenoid singlet oxygen quencher. Arch Biochem Biophys. (1989) 274:532–8. doi: 10.1016/0003-9861(89)90467-0 2802626

[113] Fatima M Rakha A Altemimi AB Van Bockstaele F Khan AI Ayyub M . Okra: mucilage extraction, composition, applications, and potential health benefits. Eur Polym J. (2024) 215:113193. doi: 10.1016/j.eurpolymj.2024.113193

[114] Agregán R Pateiro M Bohrer BM Shariati MA Nawaz A Gohari G . Biological activity and development of functional foods fortified with okra (*Abelmoschus esculentus*). Crit Rev Food Sci Nutr. (2022) 63:6018–33. doi: 10.1080/10408398.2022.2026874 35037792

[115] Kumar D Lal MK Dutt S Raigond P Changan SS Tiwari RK . Functional fermented probiotics, prebiotics, and synbiotics from non-dairy products: a perspective from nutraceutical. Mol Nutr Food Res. (2022) 66:2101059. doi: 10.1002/mnfr.202101059 35616160

[116] Zaini NSM Khudair AJD Gengan G Rahim MHA Hussin ASM Idris H . Enhancing the nutritional profile of vegan diet: a review of fermented plant-based milk as a nutritious supplement. J Food Compos Anal. (2023) 123:105567. doi: 10.1016/j.jfca.2023.105567

[117] Francis DV Dahiya D Gokhale T Nigam PS. Sustainable packaging materials for fermented probiotic dairy or non-dairy food and beverage products: challenges and innovations. AIMS Microbiol. (2024) 10:320–39. doi: 10.3934/microbiol.2024017 38919715 PMC11194616

[118] Aboueldis GR Abdelazeez WMA Suliman AA Mohammed DM. Therapeutic efficacy of secondary metabolites produced from cell suspension culture of *Vaccinium corymbosum* L. mitigates high-fat-diet-induced metabolic syndrome in rat model. Food Biosci. (2025) 68:106795. doi: 10.1016/j.fbio.2025.106795

[119] Riar CS Panesar PS. Bioactive Compounds and Nutraceuticals from Dairy, Marine, and Nonconventional Sources. New York, NY: Apple Academic Press (2024). p. 324. doi: 10.1201/9781003452768

[120] Walia A Gupta AK Sharma V. Role of bioactive compounds in human health. Acta Sci Med Sci. (2019) 3:25–33.

[121] Dahiya D Terpou A Dasenaki M Nigam PS. Current status and future prospects of bioactive molecules delivered through sustainable encapsulation techniques for food fortification. Sustain Food Technol. (2023) 1:500–10. doi: 10.1039/D3FB00015J

[122] Ghosh S Sarkar T Pati S Kari ZA Edinur HA Chakraborty R. Novel bioactive compounds from marine sources as a tool for functional food development. Front Mar Sci. (2022) 9:832957. doi: 10.3389/fmars.2022.832957

[123] Nasrollahzadeh M Sajjadi M Nezafat Z Shafiei N. Polysaccharide biopolymer chemistry. In:Nasrollahzadeh M, editor. Biopolymer Based Metal Nanoparticle Chemistry for Sustainable Applications. Amsterdam: Elsevier (2021). p. 45–105. doi: 10.1016/B978-0-12-822108-2.00019-3

[124] Chen H Jia Y Guo Q. Polysaccharides and polysaccharide complexes as potential sources of antidiabetic compounds: a review. Stud Nat Prod Chem. (2020) 67:199–220. doi: 10.1016/B978-0-12-819483-6.00006-0

[125] Clemente-Suárez VJ Mielgo-Ayuso J Martín-Rodríguez A Ramos-Campo DJ Redondo-Flórez L Tornero-Aguilera JF. The burden of carbohydrates in health and disease. Nutrients. (2022) 14:3809. doi: 10.3390/nu14183809 36145184 PMC9505863

[126] Yuan D Li C Huang Q Fu X Dong H. Current advances in the anti-inflammatory effects and mechanisms of natural polysaccharides. Crit Rev Food Sci Nutr. (2022) 63:5890–910. doi: 10.1080/10408398.2022.2025535 35021901

[127] Meng F Li Q Qi Y He C Wang C Zhang Q. Characterization and immunoregulatory activity of two polysaccharides from the root of *Ilex asprella*. Carbohydr Polym. (2018) 197:9–16. doi: 10.1016/j.carbpol.2018.05.066 30007662

[128] Liu M Li S Wang X Zhu Y Zhang J Liu H . Characterization, anti-oxidation and anti-inflammation of polysaccharides by *Hypsizygus marmoreus* against LPS-induced toxicity on lung. Int J Biol Macromol. (2018) 111:121–8. doi: 10.1016/j.ijbiomac.2018.01.010 29307806

[129] Xu B Li S Ding W Zhang C Rehman MU Tareen MF . From structure to function: a comprehensive overview of polysaccharide roles and applications. Food Front. (2024) 6:15–39. doi: 10.1002/fft2.490

[130] Monteiro V Colonetti K Pagno CH Schmidt HO Sperb-Ludwig F De Oliveira BM . Potential use of other starch sources in the treatment of glycogen storage disease type Ia – an *in vitro* study. Orphanet J Rare Dis. (2024) 19:1. doi: 10.1186/s13023-024-03201-1 39080776 PMC11289971

[131] Fernandes PAR Coimbra MA. The antioxidant activity of polysaccharides: a structure-function relationship overview. Carbohydr Polym. (2023) 314:120965. doi: 10.1016/j.carbpol.2023.120965 37173007

[132] Arora S Singh D Rajput A Bhatia A Kumar A Kaur H . Plant-based polysaccharides and their health functions. Funct Foods Health Dis. (2021) 11:179–200. doi: 10.31989/ffhd.v11i4.773

[133] Fuertes-Rabanal M Rebaque D Largo-Gosens A Encina A Mélida H. Cell walls, a comparative view of the composition of cell surfaces of plants, algae and microorganisms. J Exp Bot. (2024) 76:2614–45. doi: 10.1093/jxb/erae512 PMC12223506 39705009

[134] Lovegrove A Edwards CH De Noni I Patel H El SN Grassby T . Role of polysaccharides in food, digestion, and health. Crit Rev Food Sci Nutr. (2016) 57:237–53. doi: 10.1080/10408398.2014.939263 25921546 PMC5152545

[135] Negrea M Cocan I Jianu C Alexa E Berbecea A Poiana M-A . Valorization of citrus peel byproducts: a sustainable approach to nutrient-rich jam production. Foods. (2025) 14:1339. doi: 10.3390/foods14081339 40282741 PMC12026442

[136] Benalaya I Alves G Lopes J Silva LR. A review of natural polysaccharides: sources, characteristics, properties, food, and pharmaceutical applications. Int J Mol Sci. (2024) 25:1322. doi: 10.3390/ijms25021322 38279323 PMC10816883

[137] Kalisz G Popiolek-Kalisz J. Polysaccharides: the sweet and bitter impacts on cardiovascular risk. Polymers. (2025) 17:405. doi: 10.3390/polym17030405 39940607 PMC11820192

[138] Yang Q Chang S Zhang X Luo F Li W Ren J. The fate of dietary polysaccharides in the digestive tract. Trends Food Sci Technol. (2024) 150:104606. doi: 10.1016/j.tifs.2024.104606

[139] Warren FJ Royall PG Gaisford S Butterworth PJ Ellis PR. Binding interactions of α-amylase with starch granules: the influence of supramolecular structure and surface area. Carbohydr Polym. (2011) 86:1038–47. doi: 10.1016/j.carbpol.2011.05.062

[140] Flavel M Jois M Kitchen B. Potential contributions of the methodology to the variability of glycaemic index of foods. World J Diabetes. (2021) 12:108–23. doi: 10.4239/wjd.v12.i2.108 33594331 PMC7839170

[141] Zhao JL Zhang M Zhou HL. Microwave-assisted extraction, purification, partial characterization, and bioactivity of polysaccharides from *Panax ginseng*. Molecules. (2019) 24:1605. doi: 10.3390/molecules24081605 31018583 PMC6514599

[142] Muonde NM Olorunsogo NTO Ogugua NJO Maduka NCP Omotayo NO. Global nutrition challenges: a public health review of dietary risks and interventions. World J Adv Res Rev. (2024) 21:1467–78. doi: 10.30574/wjarr.2024.21.1.0177

[143] Sharma K Kaur R Kumar S Saini RK Sharma S Pawde SV . Saponins: a concise review on food related aspects, applications and health implications. Food Chem Adv. (2023) 2:100191. doi: 10.1016/j.focha.2023.100191

[144] Rathee P Sehrawat R Rathee P Khatkar A Akkol EK Khatkar S . Polyphenols: natural preservatives with promising applications in food, cosmetics and pharma industries; problems and toxicity associated with synthetic preservatives; impact of misleading advertisements; recent trends in preservation and legislation. Materials. (2023) 16:4793. doi: 10.3390/ma16134793 37445107 PMC10343617

[145] Zhang Y Hao R Chen J Li S Huang K Cao H . Health benefits of saponins and its mechanisms: perspectives from absorption, metabolism, and interaction with gut. Crit Rev Food Sci Nutr. (2023) 64:9311–32. doi: 10.1080/10408398.2023.2212063 37216483

[146] Shin KC Kim DW Oh YJ Seo MJ Na CS Kim YS. Improved production of deglucosylated platycodin D from saponins from balloon flower leaf by a food-grade enzyme using high hydrostatic pressure. Heliyon. (2021) 7:e08104. doi: 10.1016/j.heliyon.2021.e08104 34660923 PMC8503635

[147] Nichakool B Jamphon A Pootang-On Pootang-On Y Techakriengkrai W Techakriengkrai T. A study about Brahmi (*Bacopa monnieri*) preparation steps on its saponin quantity. Trends Sci. (2021) 18:1439. doi: 10.48048/tis.2021.1439

[148] Gu Y Yang X Shang C Thao TTP Koyama T. Inhibitory properties of saponin from *Eleocharis dulcis* peel against α-glucosidase. RSC Adv. (2021) 11:15400–9. doi: 10.1039/D1RA02198B 35424054 PMC8698979

[149] Shen N Wang T Gan Q Liu S Wang L Jin B. Plant flavonoids: classification, distribution, biosynthesis, and antioxidant activity. Food Chem. (2022) 383:132531. doi: 10.1016/j.foodchem.2022.132531 35413752

[150] Dias MC Pinto DCGA Silva AMS. Plant flavonoids: chemical characteristics and biological activity. Molecules. (2021) 26:5377. doi: 10.3390/molecules26175377 34500810 PMC8434187

[151] Addi M Elbouzidi A Abid M Tungmunnithum D Elamrani A Hano C. An overview of bioactive flavonoids from citrus fruits. Appl Sci. (2021) 12:29. doi: 10.3390/app12010029

[152] Silva A Silva V Igrejas G Aires A Falco V Valentão P . Phenolic compounds classification and their distribution in winemaking by-products. Eur Food Res Technol. (2022) 249:207–39. doi: 10.1007/s00217-022-04163-z

[153] Patra S Makhal P Jaryal S More N Kaki VR. Anthocyanins: plant-based flavonoid pigments with diverse biological activities. Int J Plant Based Pharm. (2022) 2:118–27. doi: 10.62313/ijpbp.2022.22

[154] Chen Q Wang X Yuan X Shi J Zhang C Yan N . Comparison of phenolic and flavonoid compound profiles and antioxidant and α-glucosidase inhibition properties of cultivated soybean (*Glycine max*) and wild soybean (*Glycine soja*). Plants. (2021) 10:813. doi: 10.3390/plants10040813 33924154 PMC8074397

[155] Dixit V Joseph Kamal SW Bajrang Chole P Dayal D Chaubey KK Pal AK . Functional foods: exploring the health benefits of bioactive compounds from plant and animal sources. J Food Qual. (2023) 2023:5546753. doi: 10.1155/2023/5546753

[156] Chen L Cao H Huang Q Xiao J Teng H. Absorption, metabolism and bioavailability of flavonoids: a review. Crit Rev Food Sci Nutr. (2022) 62:7730–42. doi: 10.1080/10408398.2021.1917508 34078189

[157] Mozaffarian D Wu JYH. Flavonoids, dairy foods, and cardiovascular and metabolic health: a review of emerging biologic pathways. Circ Res. (2018) 122:369–84. doi: 10.1161/CIRCRESAHA.117.309008 29348256 PMC5781235

[158] Khan S Dar AH Shams R Aga MB Siddiqui MW Mir SA. Rizvi Queh, Khan SA, Altaf A. Applications of ultraviolet light–emitting diode technology in horticultural produce: a systematic review and meta-analysis. Food Bioprocess Technol. (2022) 15:487–97. doi: 10.1007/s11947-021-02742-8

[159] Gupta L Chauhan M Kumar A Chauhan D Saini P. Flavonoids and cardiovascular diseases. In:Sharma N Saini D Kesharwani RK Gupta PC Keservani RK, editors. Advances In Flavonoids for Human Health and Prevention of Diseases. New York, NY: Apple Academic Press (2024). p. 73–94. doi: 10.1201/9781003369813-4

[160] Rakha A Umar N Rabail R Butt MS Kieliszek M Hassoun A . Anti-inflammatory and anti-allergic potential of dietary flavonoids: a review. Biomed Pharmacother. (2022) 156:113945. doi: 10.1016/j.biopha.2022.113945 36411631

[161] Yao LH Jiang YM Shi J Tomás-Barberán FA Datta N Singanusong R . Flavonoids in food and their health benefits. Plant Foods Hum Nutr. (2004) 59:113–22. doi: 10.1007/s11130-004-0049-7 15678717

[162] Al Mamun A Shao C Geng P Wang S Xiao J. Polyphenols targeting NF-κB pathway in neurological disorders: what we know so far? Int J Biol Sci. (2024) 20:1332. doi: 10.7150/ijbs.90982 38385077 PMC10878147

[163] Kandar JF Rochmanti M Wungu CDK Qurnianingsih E. Cacao, the origin of chocolate, can lower lipid profiles? A systematic review. World J Adv Res Rev. (2024) 21:573–8. doi: 10.30574/wjarr.2024.21.1.0027

[164] Al-Khayri JM Sahana GR Nagella P Joseph BV Alessa FM Al-Mssallem MQ. Flavonoids as potential anti-inflammatory molecules: a review. Molecules. (2022) 27:2901. doi: 10.3390/molecules27092901 35566252 PMC9100260

[165] Hoskin DW Coombs MRP. Immune modulation by flavonoids. Front Immunol. (2022) 13:899577. doi: 10.3389/fimmu.2022.899577 35479091 PMC9035818

[166] Rajput A Sharma R Bharti R. Pharmacological activities and toxicities of alkaloids on human health. Mater Today Proc. (2022) 48:1407–15. doi: 10.1016/j.matpr.2021.09.189

[167] Shi Q Sun H Xu HY Yan GL Han Y Wang XJ. Natural alkaloids: basic aspects, biological roles, and future perspectives. Chin J Nat Med. (2014) 12:401–6. doi: 10.1016/S1875-5364(14)60063-7 24969519

[168] Zhang X Cui J Hou J Wang W. Research progress of natural active substances with uric-acid-reducing activity. J Agric Food Chem. (2022) 70:15647–64. doi: 10.1021/acs.jafc.2c06554 36482671

[169] Borsoi FT Pastore GM Arruda HS. Health benefits of the alkaloids from lobeira (*Solanum lycocarpum* St. Hill*):* a comprehensive review. Plants. (2024) 13:1396. doi: 10.3390/plants13101396 38794466 PMC11124789

[170] Heinrich M Mah J Amirkia V. Alkaloids used as medicines: structural phytochemistry meets biodiversity—an update and forward look. Molecules. (2021) 26:1836. doi: 10.3390/molecules26071836 33805869 PMC8036335

[171] Ranjitha D Sudha K. Alkaloids in foods. Int J Pharm Chem Biol Sci. (2015) 5:896–906.

[172] Luo SC Wei SM Luo XT Yang QQ Wong KH Cheung PC . How probiotics, prebiotics, synbiotics, and postbiotics prevent dental caries: an oral microbiota perspective. NPJ Biofilms Microbiomes. (2024) 10:14. doi: 10.1038/s41522-024-00488-7 38402294 PMC10894247

[173] Akinboye AJ Kim K Choi S Yang I Lee JG. Alkaloids in food: a review of toxicity, analytical methods, occurrence and risk assessments. Food Sci Biotechnol. (2023) 32:1133–58. doi: 10.1007/s10068-023-01295-0 37362815 PMC10290023

[174] Mitra S Paul S Roy S Sutradhar H Bin Emran T Nainu F . Exploring the immune-boosting functions of vitamins and minerals as nutritional food bioactive compounds: a comprehensive review. Molecules. (2022) 27:555. doi: 10.3390/molecules27020555 35056870 PMC8779769

[175] Guéant JL Guéant-Rodriguez RM Alpers DH. Vitamin B12 absorption and malabsorption. Vitam Horm. (2022) 119:241–74. doi: 10.1016/bs.vh.2022.01.016 35337622

[176] Yang L Gao Y Farag MA Gong J Su Q Cao H . Dietary flavonoids and gut microbiota interaction: a focus on animal and human studies to maximize their health benefits. Food Front. (2023) 4:1794–809. doi: 10.1002/fft2.309

[177] Karachaliou CE Livaniou E. Biotin homeostasis and human disorders: recent findings and perspectives. Int J Mol Sci. (2024) 25:6578. doi: 10.3390/ijms25126578 38928282 PMC11203980

[178] Torquato P Marinelli R Bartolini D Galli F. Vitamin E: nutritional aspects. In:Patel VB, editor. Molecular Nutrition. Amsterdam: Academic Press (2020). p. 447–85. doi: 10.1016/B978-0-12-811907-5.00019-1

[179] Xiao S Li J editors. Study on functional components of functional food based on food vitamins. J Phys Conf Ser. (2020) 1549:032002. doi: 10.1088/1742-6596/1549/3/032002

[180] Alongi M Anese M. Re-thinking functional food development through a holistic approach. J Funct Foods. (2021) 81:104466. doi: 10.1016/j.jff.2021.104466

[181] Kumar P Banik SP Ohia SE Moriyama H Chakraborty S Wang CK . Current insights on the photoprotective mechanism of the macular carotenoids, lutein and zeaxanthin: safety, efficacy and bio-delivery. J Am Nutr Assoc. (2024) 43:505–18. doi: 10.1080/27697061.2024.2319090 38393321

[182] Ubago-Guisado E Rodriguez-Barranco M Ching-Lopez A Petrova D Molina-Montes E Amiano P . Evidence update on the relationship between diet and the most common cancers from the European prospective investigation into cancer and nutrition (EPIC) study: a systematic review. Nutrients. (2021) 13:3582. doi: 10.3390/nu13103582 34684583 PMC8540388

[183] Konecki T Juszczak A Cichocki M. Can diet prevent urological cancers? An update on carotenoids as chemopreventive agents. Nutrients. (2022) 14:1367. doi: 10.3390/nu14071367 35405980 PMC9002657

[184] Sanlier N Yildiz E Ozler E. An overview on the effects of some carotenoids on health: lutein and zeaxanthin. Curr Nutr Rep. (2024) 13:828–44. doi: 10.1007/s13668-024-00579-z 39304612

[185] Abuajah CI Ogbonna AC Osuji CM. Functional components and medicinal properties of food: a review. J Food Sci Technol. (2015) 52:2522–9. doi: 10.1007/s13197-014-1396-5 25892752 PMC4397330

[186] Meléndez-Martínez AJ Esquivel P Rodriguez-Amaya DB. Comprehensive review on carotenoid composition: transformations during processing and storage of foods. Food Res Int. (2023) 169:112773. doi: 10.1016/j.foodres.2023.112773 37254377

[187] Zakynthinos G Varzakas T. Carotenoids: from plants to food industry. Curr Res Nutr Food Sci. (2016) 4:38–51. doi: 10.12944/CRNFSJ.4.Special-Issue1.04

[188] Wang L Liu Z Jiang H Mao X. Biotechnology advances in β-carotene production by microorganisms. Trends Food Sci Technol. (2021) 111:322–32. doi: 10.1016/j.tifs.2021.02.077

[189] Stra A Almarwaey LO Alagoz Y Moreno JC Al-Babili S. Carotenoid metabolism: new insights and synthetic approaches. Front Plant Sci. (2023) 13:1072061. doi: 10.3389/fpls.2022.1072061 36743580 PMC9891708

[190] Ali O Szabó A. Review of eukaryote cellular membrane lipid composition, with special attention to the fatty acids. Int J Mol Sci. (2023) 24:15693. doi: 10.3390/ijms242115693 37958678 PMC10649022

[191] Ahamad J Naquvi KJ Uthirapathy S Naim MJ Majedi S. Monounsaturated and polyunsaturated fatty acids. In:Nollet LM Ahamad J, editors. Bioactive Compounds of Edible Oils and Fats. Boca Raton, FL: CRC Press (2024). p. 71–87. doi: 10.1201/9781003450719-6

[192] Gunstone FD. Research highlights: Lipid Technology 2/2010. Lipid Technol. (2010) 22:43–5. doi: 10.1002/lite.200900081

[193] Kapoor B Kapoor D Gautam S Singh R Bhardwaj S. Dietary polyunsaturated fatty acids (PUFAs): uses and potential health benefits. Curr Nutr Rep. (2021) 10:232–42. doi: 10.1007/s13668-021-00363-3 34255301

[194] Zhu S He Y Lei JN Liu YF Xu YJ. The chemical and biological characteristics of fatty acid esters of hydroxyl fatty acids. Nutr Rev. (2025) 83:e427–42. doi: 10.1093/nutrit/nuae005 38412339

[195] Mühlroth A Li K Røkke G Winge P Olsen Y Hohmann-Marriott MF . Pathways of lipid metabolism in marine algae, co-expression network, bottlenecks and candidate genes for enhanced production of EPA and DHA in species of Chromista. Mar Drugs. (2013) 11:4662–97. doi: 10.3390/md11114662 24284429 PMC3853752

[196] Ramírez M Amate L Gil A. Absorption and distribution of dietary fatty acids from different sources. Early Hum Dev. (2001) 65:S95–S101. doi: 10.1016/S0378-3782(01)00211-0 11755040

[197] Ojha PK Poudel DK Rokaya A Maharjan S Timsina S Poudel A . Chemical compositions and essential fatty acid analysis of selected vegetable oils and fats. Compounds. (2024) 4:37–70. doi: 10.3390/compounds4010003

[198] Islam F Imran A Nosheen F Fatima M Arshad MU Afzaal M . Functional roles and novel tools for improving-oxidative stability of polyunsaturated fatty acids: a comprehensive review. Food Sci Nutr. (2023) 11:2471–82. doi: 10.1002/fsn3.3272 37324849 PMC10261796

[199] Shahidi F Peng H. Bioaccessibility and bioavailability of phenolic compounds. J Food Bioact. (2018) 4:11–68. doi: 10.31665/JFB.2018.4162

[200] El-Saadony MT Yang T Saad AM Alkafaas SS Elkafas SS Eldeeb GS . Chemistry, bioavailability, bioactivity, nutritional aspects, and human health benefits of polyphenols: a comprehensive review. Int J Biol Macromol. (2024) 277:134223. doi: 10.1016/j.ijbiomac.2024.134223 39084416

[201] Mohammed DM Maan SA Abou Baker DH Abozed SS. *In vitro* assessments of antioxidant, antimicrobial, cytotoxicity and anti-inflammatory characteristics of flavonoid fractions from flavedo and albedo orange peel as novel food additives. Food Biosci. (2024) 62:105581. doi: 10.1016/j.fbio.2024.105581

[202] Machu L Misurcova L Vavra Ambrozova J Orsavova J Mlcek J Sochor J . Phenolic content and antioxidant capacity in algal food products. Molecules. (2015) 20:1118–33. doi: 10.3390/molecules20011118 25587787 PMC6272319

[203] Parcheta M Swisłocka R Orzechowska S Akimowicz M Choińska R Lewandowski W. Recent developments in effective antioxidants: the structure and antioxidant properties. Materials. (2021) 14:1984. doi: 10.3390/ma14081984 33921014 PMC8071393

[204] Ghouari N Benali-Cherif R Takouachet R Falek W Missaoui D Rahmouni A . Crystal engineering of a new pharmaceutical polymorph of gallic acid monohydrate: a structural comparative study and chemical computational quantum investigations. CrystEngComm. (2023) 25:6279–90. doi: 10.1039/D3CE00766A

[205] Zeb A. Concept, mechanism, and applications of phenolic antioxidants in foods. J Food Biochem. (2020) 44:e13394. doi: 10.1111/jfbc.13394 32691460

[206] Gutiérrez-del-Río I López-Ibáñez S Magadán-Corpas P Fernández-Calleja L Pérez-Valero Á Tuñón-Granda M . Terpenoids and polyphenols as natural antioxidant agents in food preservation. Antioxidants. (2021) 10:1264. doi: 10.3390/antiox10081264 34439512 PMC8389302

[207] Abou Baker DH Mohammed DM. Polyphenolic rich fraction of *Physalis peruviana* calyces and its nano emulsion induce apoptosis by caspase 3 up-regulation and G2/M arrest in hepatocellular carcinoma. Food Biosci. (2022) 50:102007. doi: 10.1016/j.fbio.2022.102007

[208] Rudrapal M Khairnar SJ Khan J Dukhyil AB Ansari MA Alomary MN . Dietary polyphenols and their role in oxidative stress-induced human diseases: insights into protective effects, antioxidant potentials and mechanism(s) of action. Front Pharmacol. (2022) 13:806470. doi: 10.3389/fphar.2022.806470 35237163 PMC8882865

[209] Zhou L Elias RJ. Understanding antioxidant and prooxidant mechanisms of phenolics in food lipids. In:Logan A Nienaber U Pan X, editors. Lipid Oxidation. Cambridge, MA: Academic Press and AOCS Press (2013). p. 297–321. doi: 10.1016/B978-0-9830791-6-3.50012-6

[210] Othón-Díaz ED Fimbres-García JO Flores-Sauceda M Silva-Espinoza BA López-Martínez LX Bernal-Mercado AT. Ayala-Zavala JF. Antioxidants in oak (*Quercus* sp.): potential application to reduce oxidative rancidity in foods. Antioxidants. (2023) 12:861. doi: 10.3390/antiox12040861 37107236 PMC10135015

[211] Shahidi F Ambigaipalan P. Phenolics and polyphenolics in foods, beverages and spices: antioxidant activity and health effects–a review. J Funct Foods. (2015) 18:820–97. doi: 10.1016/j.jff.2015.06.018

[212] Sadgrove NJ Padilla-González GF Phumthum M. Fundamental chemistry of essential oils and volatile organic compounds, methods of analysis and authentication. Plants. (2022) 11:789. doi: 10.3390/plants11060789 35336671 PMC8955314

[213] Zuzarte M Girão H Salgueiro L. Aromatic plant-based functional foods: a natural approach to manage cardiovascular diseases. Molecules. (2023) 28:5130. doi: 10.3390/molecules28135130 37446792 PMC10343196

[214] Zuzarte M Salgueiro L. Essential oils chemistry. In:de Sousa D, editor. Bioactive Essential Oils and Cancer. Cham: Springer (2015). p. 19–61. doi: 10.1007/978-3-319-19144-7_2

[215] Al-Maqtari QA Rehman A Mahdi AA Al-Ansi W Wei M Yanyu Z . Application of essential oils as preservatives in food systems: challenges and future prospectives–a review. Phytochem Rev. (2022) 21:1209–46. doi: 10.1007/s11101-021-09776-y

[216] Tsitlakidou P Tasopoulos N Chatzopoulou P Mourtzinos I. Current status, technology, regulation and future perspectives of essential oils usage in the food and drink industry. J Sci Food Agric. (2023) 103:6727–51. doi: 10.1002/jsfa.12695 37158299

[217] Jackson-Davis A White S Kassama LS Coleman S Shaw A Mendonca A . A review of regulatory standards and advances in essential oils as antimicrobials in foods. J Food Prot. (2023) 86:100025. doi: 10.1016/j.jfp.2022.100025 36916569

[218] Carpena M Nuñez-Estevez B Soria-Lopez A Garcia-Oliveira P Prieto MA. Essential oils and their application on active packaging systems: a review. Resources. (2021) 10:7. doi: 10.3390/resources10010007

[219] Saeed K Pasha I Chughtai MFJ Ali Z Bukhari H Zuhair M. Application of essential oils in food industry: challenges and innovation. J Essent Oil Res. (2022) 34:97–110. doi: 10.1080/10412905.2022.2029776

[220] Barkas F Bathrellou E Nomikos T Panagiotakos D Liberopoulos E Kontogianni MD. Plant sterols and plant stanols in cholesterol management and cardiovascular prevention. Nutrients. (2023) 15:2845. doi: 10.3390/nu15132845 37447172 PMC10343346

[221] Nattagh-Eshtivani E Barghchi H Pahlavani N Barati M Amiri Y Fadel A . Biological and pharmacological effects and nutritional impact of phytosterols: a comprehensive review. Phytother Res. (2022) 36:299–322. doi: 10.1002/ptr.7312 34729825

[222] Piironen V Lampi AM. Occurrence and levels of phytosterols in foods. In:Dutta PC, editor. Phytosterols as Functional Food Components and Nutraceuticals. Boca Raton, FL: CRC Press (2003). p. 9–13. doi: 10.1201/9780203913413.ch1

[223] Gylling H Simonen P. Phytosterols, phytostanols, and lipoprotein metabolism. Nutrients. (2015) 7:7965–77. doi: 10.3390/nu7095374 26393644 PMC4586569

[224] Salehi B Quispe C Sharifi-Rad J Cruz-Martins N Nigam M Mishra AP . Phytosterols: from preclinical evidence to potential clinical applications. Front Pharmacol. (2021) 11:599959. doi: 10.3389/fphar.2020.599959 33519459 PMC7841260

[225] Pant SP Joshi S Bisht D Bisht M. Exploring the historical, botanical, and taxonomical foundations of cannabis: a review. In:Shukla R Handa M Singh DP Dhir A, editors. Cannabis and its Derivatives. Amsterdam: Academic Press (2024). p. 3–36. doi: 10.1016/B978-0-443-15489-8.00001-3

[226] Montserrat-De La Paz S Marín-Aguilar F García-Gimenez MD. Fernández-Arche MA. Hemp (*Cannabis sativa* L.) seed oil: analytical and phytochemical characterization of the unsaponifiable fraction. J Agric Food Chem. (2014) 62:1105–10. doi: 10.1021/jf404278q 24422510

[227] Krüger M van Eeden T Beswa D. Cannabis sativa cannabinoids as functional ingredients in snack foods—historical and developmental aspects. Plants. (2022) 11:3330. doi: 10.3390/plants11233330 PMC9739163 36501366

[228] Gülck T Møller BL. Phytocannabinoids: origins and biosynthesis. Trends Plant Sci. (2020) 25:985–1004. doi: 10.1016/j.tplants.2020.05.005 32646718

[229] ElSohly MA Radwan MM Gul W Chandra S Galal A. Phytochemistry of *Cannabis sativa* L. In:Douglas Kinghorn A Falk H Gibbons S Kobayashi J, editors. Phytocannabinoids: Unraveling the Complex Chemistry and Pharmacology of Cannabis sativa. Cham: Springer (2017). p. 1–36. doi: 10.1007/978-3-319-45541-9_1 28120229

[230] Morales P Hurst DP Reggio PH. Molecular targets of the phytocannabinoids: a complex picture. Prog Chem Org Nat Prod. (2017) 103:103–31. doi: 10.1007/978-3-319-45541-9_4 28120232 PMC5345356

[231] Radwan MM Chandra S Gul S ElSohly MA. Cannabinoids, phenolics, terpenes and alkaloids of cannabis. Molecules. (2021) 26:2774. doi: 10.3390/molecules26092774 34066753 PMC8125862

[232] Conerney C Steinmetz F Wakefield J Loveridge S. Cannabis and children: risk mitigation strategies for edibles. Front Psychiatry. (2024) 15:1285784. doi: 10.3389/fpsyt.2024.1285784 PMC10876888 38380122

[233] Peng H Shahidi F. Cannabis and cannabis edibles: a review. J Agric Food Chem. (2021) 69:1751–74. doi: 10.1021/acs.jafc.0c07472 33555188

[234] Fordjour E Manful CF Khalsamehta TS Armah A Cheema M Thomas R. Cannabis-infused foods: phytonutrients, health, and safe product innovations. Compr Rev Food Sci Food Saf. (2024) 23:e70021. doi: 10.1111/1541-4337.70021 39267188

[235] Lajoie L Fabiano-Tixier AS Chemat F. Water as green solvent: methods of solubilization and extraction of natural products—past, present and future solutions. Pharmaceuticals. (2022) 15:1507. doi: 10.3390/ph15121507 36558959 PMC9788067

[236] Romano G Costantini M Sansone C Lauritano C Ruocco N Ianora A. Marine microorganisms as a promising and sustainable source of bioactive molecules. Mar Environ Res. (2017) 128:58–69. doi: 10.1016/j.marenvres.2016.05.002 27160988

[237] Bhadange YA Carpenter J Saharan VK. A comprehensive review on advanced extraction techniques for retrieving bioactive components from natural sources. ACS Omega. (2024) 9:31274–97. doi: 10.1021/acsomega.4c02718 39072073 PMC11270575

[238] Azmir J Zaidul ISM Rahman MM Sharif KM Mohamed A Sahena F . Techniques for extraction of bioactive compounds from plant materials: a review. J Food Eng. (2013) 117:426–36. doi: 10.1016/j.jfoodeng.2013.01.014

[239] Paczkowska-Walendowska M Cielecka-Piontek J. Chitosan as a functional carrier for the local delivery anti-inflammatory systems containing *Scutellariae baicalensis* radix extract. Pharmaceutics. (2022) 14:2148. doi: 10.3390/pharmaceutics14102148 36297583 PMC9611887

[240] Cannavacciuolo C Pagliari S Celano R Campone L Rastrelli L. Critical analysis of green extraction techniques used for botanicals: trends, priorities, and optimization strategies—a review. TrAC Trends Anal Chem. (2024) 173:117627. doi: 10.1016/j.trac.2024.117627

[241] Lefebvre T Destandau E Lesellier E. Selective extraction of bioactive compounds from plants using recent extraction techniques: a review. J Chromatogr A. (2021) 1635:461770. doi: 10.1016/j.chroma.2020.461770 33310280

[242] Chuo SC Nasir HM Mohd-Setapar SH Mohamed SF Ahmad A Wani WA . A glimpse into the extraction methods of active compounds from plants. Crit Rev Anal Chem. (2022) 52:667–96. doi: 10.1080/10408347.2020.1820851 32954795

[243] Shrivastav G Prava Jyoti T Chandel S Singh R. Eco-friendly extraction: innovations, principles, and comparison with traditional methods. Sep Purif Rev. (2025) 54:241–57. doi: 10.1080/15422119.2024.2381605

[244] Chemat F Vian MA Fabiano-Tixier AS Nutrizio M Jambrak AR Munekata PE . A review of sustainable and intensified techniques for extraction of food and natural products. Green Chem. (2020) 22:2325–53. doi: 10.1039/C9GC03878G

[245] Picot-Allain C Mahomoodally MF Ak G Zengin G. Conventional versus green extraction techniques—a comparative perspective. Curr Opin Food Sci. (2021) 40:144–56. doi: 10.1016/j.cofs.2021.02.009

[246] Usman M Nakagawa M Cheng S. Emerging trends in green extraction techniques for bioactive natural products. Processes. (2023) 11:3444. doi: 10.3390/pr11123444

[247] Chen SY Urban PL. On-line monitoring of Soxhlet extraction by chromatography and mass spectrometry to reveal temporal extract profiles. Anal Chim Acta. (2015) 881:74–81. doi: 10.1016/j.aca.2015.05.003 26041522

[248] Kodal SP Aksu Z. Optimization of carotene pigment production by Soxhlet extraction from waste orange peels. Food Chem. (2001) 72:145–71.

[249] Caldas TW Mazza KE Teles AS Mattos GN Brígida AIS Conte-Junior CA . Phenolic compounds recovery from grape skin using conventional and non-conventional extraction methods. Ind Crops Prod. (2018) 111:86–91. doi: 10.1016/j.indcrop.2017.10.012

[250] Tian B Qiao YY Tian YY Xie KC Li DW. Effect of heat reflux extraction on the structure and composition of a high-volatile bituminous coal. Appl Therm Eng. (2016) 109:560–8. doi: 10.1016/j.applthermaleng.2016.08.104

[251] Romero-Cascales I Fernández-Fernández JI López-Roca JM Gómez-Plaza E. The maceration process during winemaking extraction of anthocyanins from grape skins into wine. Eur Food Res Technol. (2005) 221:163–7. doi: 10.1007/s00217-005-1144-1

[252] Sultana B Anwar F Asi MR Chatha SAS. Antioxidant potential of extracts from different agro wastes: stabilization of corn oil. Grasas Aceites. (2008) 59:205–17. doi: 10.3989/gya.2008.v59.i3.510

[253] Albuquerque BR Prieto MA Barreiro MF Rodrigues A Curran TP Barros L . Catechin-based extract optimization obtained from *Arbutus unedo* L. fruits using maceration/microwave/ultrasound extraction techniques. Ind Crops Prod. (2017) 95:404–15. doi: 10.1016/j.indcrop.2016.10.050

[254] Khoddami A Wilkes MA Roberts TH. Techniques for analysis of plant phenolic compounds. Molecules. (2013) 18:2328–75. doi: 10.3390/molecules18022328 23429347 PMC6270361

[255] Herzyk F Piłakowska-Pietras D Korzeniowska M. Supercritical extraction techniques for obtaining biologically active substances from a variety of plant byproducts. Foods. (2024) 13:1713. doi: 10.3390/foods13111713 38890941 PMC11171758

[256] Branch JA Bartlett PN. Electrochemistry in supercritical fluids. Philos Trans R Soc A Math Phys Eng Sci. (2015) 373:20150007. doi: 10.1098/rsta.2015.0007 26574527 PMC4650015

[257] Hannay JB Hogarth J. VI. On the solubility of solids in gases. Proc R Soc Lond. (1879) 29:324–6. doi: 10.1098/rspl.1879.0054

[258] Raventós M Duarte S Alarcón R. Application and possibilities of supercritical CO_2_ extraction in food processing industry: an overview. Food Sci Technol Int. (2002) 8:269–84. doi: 10.1106/108201302029451

[259] Aili Q Cui D Li Y Zhige W Yongping W Minfen Y . Composing functional food from agro-forest wastes: selectively extracting bioactive compounds using supercritical fluid extraction. Food Chem. (2024) 455:139848. doi: 10.1016/j.foodchem.2024.139848 38823122

[260] Magalhães S Fernandes C Pedrosa JF Alves L Medronho B Ferreira PJ . Eco-friendly methods for extraction and modification of cellulose: an overview. Polymers. (2023) 15:3138. doi: 10.3390/polym15143138 37514527 PMC10386580

[261] Rodrigues VM Sousa EM Monteiro AR Chiavone-Filho O Marques MO Meireles MAA. Determination of the solubility of extracts from vegetable raw material in pressurized CO_2_: a pseudo-ternary mixture formed by cellulosic structure + solute + solvent. J Supercrit Fluids. (2002) 22:21–36. doi: 10.1016/S0896-8446(01)00108-5

[262] Sodeifian G Usefi MMB. Solubility, extraction, and nanoparticles production in supercritical carbon dioxide: a mini-review. ChemBioEng Rev. (2023) 10:133–66. doi: 10.1002/cben.202200020

[263] Pereira CG Meireles MAA. Supercritical fluid extraction of bioactive compounds: fundamentals, applications and economic perspectives. Food Bioprod Process. (2010) 3:340–72. doi: 10.1007/s11947-009-0263-2

[264] Shi J Kang X Mao L Jiang Y Zhao S Liu Y . Supercritical CO_2_-applied equipment for chemical synthesis and transformation: current status and perspectives. Chem Eng J. (2023) 459:141608. doi: 10.1016/j.cej.2023.141608

[265] Temelli F Guculu-Ustundag O. Supercritical technologies for further processing of edible oils. In:Shahidi F, editor. Bailey's Industrial Oil and Fat Products. Hoboken, NJ: John Wiley & Sons, Inc. (2005). p. 397–432. doi: 10.1002/047167849X.bio057

[266] Pal N Zhang X Ali M Mandal A Hoteit H. Carbon dioxide thickening: a review of technological aspects, advances and challenges for oilfield application. Fuel. (2022) 315:122947. doi: 10.1016/j.fuel.2021.122947

[267] Lang Q Wai CM. Supercritical fluid extraction in herbal and natural product studies—A practical review. Talanta. (2001) 53:771–82. doi: 10.1016/S0039-9140(00)00557-9 18968166

[268] Ghafoor K Park J. Choi YH. Optimization of supercritical fluid extraction of bioactive compounds from grape (*Vitis labrusca* B.) peel by using response surface methodology. Innov Food Sci Emerg Technol. (2010) 11:485–90. doi: 10.1016/j.ifset.2010.01.013

[269] Jaimand K Rezaee MB Azimi R Fekri-Qomi S Yahyazadeh M Karimi S . A major loss of phenyl ethyl alcohol by the distillation procedure of *Rosa damascene* Mill. J Med Plants By-Prod. (2023) 12:1–10. doi: 10.22034/jmpb.2023.358636.1473

[270] Ibañez E Herrero M Mendiola JA Castro-Puyana M. Extraction and characterization of bioactive compounds with health benefits from marine resources: macro and micro algae, cyanobacteria, and invertebrates. In:Hayes M, editor. Marine Bioactive Compounds: Sources, Characterization and Applications. Boston, MA: Springer (2011). p. 55–98. doi: 10.1007/978-1-4614-1247-2_2

[271] Vafaei N Rempel CB Scanlon MG Jones PJ Eskin MN. Application of supercritical fluid extraction (SFE) of tocopherols and carotenoids (hydrophobic antioxidants) compared to non-SFE methods. ApplChem. (2022) 2:68–92. doi: 10.3390/appliedchem2020005

[272] Dashtian K Kamalabadi M Ghoorchian A Ganjali MR Rahimi-Nasrabadi M. Integrated supercritical fluid extraction of essential oils. J Chromatogr A. (2024) 1733:465240. doi: 10.1016/j.chroma.2024.465240 39154494

[273] Versteeg FA Picchioni F Versteeg GF. On the mass transfer of supercritical fluids, specifically supercritical CO_2_: an overview. Chem Eng J. (2024) 493:152521. doi: 10.1016/j.cej.2024.152521

[274] Da Silva RP Rocha-Santos TA Duarte AC. Supercritical fluid extraction of bioactive compounds. TrAC Trends Anal Chem. (2016) 76:40–51. doi: 10.1016/j.trac.2015.11.013

[275] Jin Y Hu D Chen Q Shi C Ye J Dai Z . Water-based green and sustainable extraction protocols for value-added compounds from natural resources. Curr Opin Green Sustain Chem. (2023) 40:100757. doi: 10.1016/j.cogsc.2023.100757

[276] Nastić N Švarc-Gajić J Delerue-Matos C Barroso MF Soares C Moreira MM . Subcritical water extraction as an environmentally-friendly technique to recover bioactive compounds from traditional Serbian medicinal plants. Ind Crops Prod. (2018) 111:579–89. doi: 10.1016/j.indcrop.2017.11.015

[277] Aminzai MT Yabalak E Akay S Kayan B. Recent developments in subcritical water extraction of industrially important bioactive substances from plants, microorganisms, and organic wastes. Biomass Convers Biorefin. (2024) 15:17927–49. doi: 10.1007/s13399-024-06392-6

[278] Herrero M Cifuentes A Ibañez E. Sub- and supercritical fluid extraction of functional ingredients from different natural sources: plants, food-by-products, algae and microalgae: a review. Food Chem. (2006) 98:136–48. doi: 10.1016/j.foodchem.2005.05.058

[279] Zakaria SM Kamal SMM. Subcritical water extraction of bioactive compounds from plants and algae: applications in pharmaceutical and food ingredients. Food Eng Rev. (2016) 8:23–34. doi: 10.1007/s12393-015-9119-x

[280] Getachew AT Chun BS. Molecular modification of native coffee polysaccharide using subcritical water treatment: structural characterization, antioxidant, and DNA protecting activities. Int J Biol Macromol. (2017) 99:555–62. doi: 10.1016/j.ijbiomac.2017.03.034 28283450

[281] Zhang J Wen C Zhang H Duan Y Ma H. Recent advances in the extraction of bioactive compounds with subcritical water: a review. Trends Food Sci Technol. (2020) 95:183–95. doi: 10.1016/j.tifs.2019.11.018

[282] Thivya P Malini B Karunanithi S Gupta RK. Effect of sub-and supercritical fluid on oil extraction and its quality. In:Srivastav PP Karunanithi S, editors. Emerging Methods for Oil Extraction from Food Processing Waste. Boca Raton, FL: CRC Press (2024). p. 1–21. doi: 10.1201/9781003408567-11

[283] Gbashi S Adebo OA Piater L Madala NE Njobeh PB. Subcritical water extraction of biological materials. Sep Purif Rev. (2017) 46:21–34. doi: 10.1080/15422119.2016.1170035

[284] Xu D Huang C Wang S Guo Y. Characteristics analysis of water film in transpiring wall reactor. Int J Heat Mass Transf. (2016) 100:559–65. doi: 10.1016/j.ijheatmasstransfer.2016.04.090

[285] AlYammahi J Rambabu K Thanigaivelan A Bharath G Hasan SW Show PL . Advances of non-conventional green technologies for phyto-saccharides extraction: current status and future perspectives. Phytochem Rev. (2023) 22:1067–88. doi: 10.1007/s11101-022-09831-2

[286] Petromelidou S Alampanos V Haj-Yahya A Lazarides T Lambropoulou DA. A green method for the determination of PFAS in environmental water matrices: dispersive solid phase extraction using MOF NH2-UiO-66 and high-resolution mass spectrometry analysis. Green Anal Chem. (2025) 12:100235. doi: 10.1016/j.greeac.2025.100235

[287] Ong ES Cheong JSH Goh D. Pressurized hot water extraction of bioactive or marker compounds in botanicals and medicinal plant materials. J Chromatogr A. (2006) 1112:92–102. doi: 10.1016/j.chroma.2005.12.052 16388815

[288] Naz S Xu TB. A comprehensive review of piezoelectric ultrasonic motors: classifications, characterization, fabrication, applications, and future challenges. Micromachines. (2024) 15:1170. doi: 10.3390/mi15091170 39337830 PMC11433840

[289] Chavan P Sharma P Sharma SR Mittal TC Jaiswal AK. Application of high-intensity ultrasound to improve food processing efficiency: a review. Foods. (2022) 11:122. doi: 10.3390/foods11010122 35010248 PMC8750622

[290] Huang H Zheng Y Chang M Song J Xia L Wu C . Ultrasound-based micro-/nanosystems for biomedical applications. Chem Rev. (2024) 124:8307–472. doi: 10.1021/acs.chemrev.4c00009 38924776

[291] Anastas PT Zimmerman JB. Peer reviewed: design through the 12 principles of green engineering. Environ Sci Technol. (2003) 37:94A. doi: 10.1021/es032373g 12666905

[292] Kumar K Srivastav S Sharanagat VS. Ultrasound assisted extraction (UAE) of bioactive compounds from fruit and vegetable processing by-products: a review. Ultrason Sonochem. (2021) 70:105325. doi: 10.1016/j.ultsonch.2020.105325 32920300 PMC7786612

[293] Zhao F Wang Z Huang H. Physical cell disruption technologies for intracellular compound extraction from microorganisms. Processes. (2024) 12:2059. doi: 10.3390/pr12102059

[294] Usman I Hussain M Imran A Afzaal M Saeed F Javed M . Traditional and innovative approaches for the extraction of bioactive compounds. Int J Food Properties. (2022) 25:1215–33. doi: 10.1080/10942912.2022.2074030

[295] Lavilla I Bendicho C. Fundamentals of ultrasound-assisted extraction. In:Dominguez González H González Muñoz MJ, editors. Water Extraction of Bioactive Compounds. Amsterdam: Elsevier (2017). p. 291–316. doi: 10.1016/B978-0-12-809380-1.00011-5

[296] Tabaraki R Heidarizadi E. Benvidi A. Optimization of ultrasonic-assisted extraction of pomegranate (*Punica granatum* L.) peel antioxidants by response surface methodology. Sep Purif Technol. (2012) 98:16–23. doi: 10.1016/j.seppur.2012.06.038

[297] Cauduro VH Gohlke G da Silva NW Cruz AG Flores EM. A review on scale-up approaches for ultrasound-assisted extraction of natural products. Curr Opin Chem Eng. (2025) 48:101120. doi: 10.1016/j.coche.2025.101120

[298] Vilkhu K Mawson R Simons L Bates D. Applications and opportunities for ultrasound assisted extraction in the food industry—A review. Innov Food Sci Emerg Technol. (2008) 9:161–9. doi: 10.1016/j.ifset.2007.04.014

[299] Sharayei P Azarpazhooh E Zomorodi S. Ramaswamy HS. Ultrasound assisted extraction of bioactive compounds from pomegranate (*Punica granatum* L.) peel LWT. Food Sci Technol. (2019) 101:342–50. doi: 10.1016/j.lwt.2018.11.031

[300] Chemat F Rombaut N Sicaire A-G Meullemiestre A Fabiano-Tixier A-S Abert-Vian M. Ultrasound assisted extraction of food and natural products. Mechanisms, techniques, combinations, protocols and applications: a review. Ultrason Sonochem. (2017) 34:540–60. doi: 10.1016/j.ultsonch.2016.06.035 27773280

[301] Islam M Malakar S Rao MV Kumar N Sahu JK. Recent advancement in ultrasound-assisted novel technologies for the extraction of bioactive compounds from herbal plants: a review. Food Sci Biotechnol. (2023) 32:1763–82. doi: 10.1007/s10068-023-01346-6 37781053 PMC10541372

[302] Gaber MAFM Juliano P. The use of low-and high-frequency ultrasound energy in food separation. In:Bermudez-Aguirre D, editor. Innovative Food Packaging and Processing Technologies. Cambridge, MA: Academic Press (2025). p. 109–48. doi: 10.1016/B978-0-323-91742-1.00005-2

[303] Chemat F Tomao V Virot M. Ultrasound-assisted extraction in food analysis. In:Ötles S, editor. Handbook of Food Analysis Instruments. Boca Raton, FL: CRC Press (2008). p. 85–103. doi: 10.1201/9781420045673.ch5

[304] Khadhraoui B Ummat V Tiwari B Fabiano-Tixier A Chemat F. Review of ultrasound combinations with hybrid and innovative techniques for extraction and processing of food and natural products. Ultrason Sonochem. (2021) 76:105625. doi: 10.1016/j.ultsonch.2021.105625 34147916 PMC8225985

[305] Nonglait DL Gokhale JS. Review insights on the demand for natural pigments and their recovery by emerging microwave-assisted extraction (MAE). Food Bioprocess Technol. (2024) 17:1681–705. doi: 10.1007/s11947-023-03192-0

[306] Alara OR Abdurahman NH Abdul Mudalip SK. Optimizing microwave-assisted extraction conditions to obtain phenolic-rich extract from *Chromolaena odorata* leaves. Chem Eng Technol. (2019) 42:1733–40. doi: 10.1002/ceat.201800462

[307] Nour AH Oluwaseun AR Nour AH Omer MS Ahmed N. Microwave-assisted extraction of bioactive compounds. In:Churyumov GI, editor. Microwave Heating—electromagnetic Fields Causing Thermal and Non-thermal Effects. London: IntechOpen (2021). p. 1–31. doi: 10.5772/intechopen.96092

[308] Deo S Janghel A Raut P Bhosle D Verma C Kumar SS . Emerging microwave assisted extraction (MAE) techniques as an innovative green technologies for the effective extraction of the active phytopharmaceuticals. Res J Pharm Technol. (2015) 8:655. doi: 10.5958/0974-360X.2015.00104.3

[309] Maqbool M. An Introduction to Non-ionizing Radiation. Singapore: Bentham Science Publishers (2023). p. 377. doi: 10.2174/97898151368901230101

[310] Radoiu M Mello A. Technical advances, barriers, and solutions in microwave—assisted technology for industrial processing. Chem Eng Res Des. (2022) 181:331–42. doi: 10.1016/j.cherd.2022.03.029

[311] Routray W Orsat V. Microwave-assisted extraction of flavonoids: a review. Food Bioprocess Technol. (2012) 5:409–24. doi: 10.1007/s11947-011-0573-z

[312] Hu Q He Y Wang F Wu J Ci Z Chen L . Microwave technology: a novel approach to the transformation of natural metabolites. Chin Med. (2021) 16:1–22. doi: 10.1186/s13020-021-00500-8 34530887 PMC8444431

[313] Ullah N Tuzen M. A comprehensive review on recent developments and future perspectives of switchable solvents and their applications in sample preparation techniques. Green Chem. (2023) 25:1729–48. doi: 10.1039/D3GC00020F

[314] López-Salazar H Camacho-Díaz BH Ocampo MA Jiménez-Aparicio AR. Microwave-assisted extraction of functional compounds from plants: a review. Bioresources. (2023) 18:6614. doi: 10.15376/biores.18.3.Lopez-Salazar

[315] Kaufmann B Christen P. Recent extraction techniques for natural products: microwave-assisted extraction and pressurized solvent extraction. Phytochem Anal. (2002) 13:105–13. doi: 10.1002/pca.631 12018022

[316] Airouyuwa JO Souka U. Maqsood S. Utilization of accelerated solvent extraction and deep eutectic solvents as synergistic green extraction technique for the recovery of bioactive compounds from date palm (*Phoenix dactylifera* L.) seeds. J Mol Liq. (2025) 425:127185. doi: 10.1016/j.molliq.2025.127185

[317] Khajeh M Reza Akbari Moghaddam A Sanchooli E. Application of Doehlert design in the optimization of microwave-assisted extraction for determination of zinc and copper in cereal samples using FAAS. Food Anal Methods. (2010) 3:133–7. doi: 10.1007/s12161-009-9099-7

[318] Jha D Maheshwari P Singh Y Haider MB Kumar R Balathanigaimani M . comparative review of extractive desulfurization using designer solvents: ionic liquids and deep eutectic solvents. J Energy Inst. (2023) 110:101313. doi: 10.1016/j.joei.2023.101313

[319] Sridhar A Vaishampayan V Kumar PS Ponnuchamy M Kapoor A. Extraction techniques in food industry: insights into process parameters and their optimization. Food Chem Toxicol. (2022) 166:113207. doi: 10.1016/j.fct.2022.113207 35688271

[320] Naliyadhara N Kumar A Girisa S Daimary UD Hegde M Kunnumakkara AB. Pulsed electric field (PEF): avant-garde extraction escalation technology in food industry. Trends Food Sci Technol. (2022) 122:238–55. doi: 10.1016/j.tifs.2022.02.019

[321] Yan LG He L Xi J. High intensity pulsed electric field as an innovative technique for extraction of bioactive compounds—a review. Crit Rev Food Sci Nutr. (2017) 57:2877–88. doi: 10.1080/10408398.2015.1077193 26462547

[322] Ganeva V Galutzov B. Electropulsation as an alternative method for protein extraction from yeast. FEMS Microbiol Lett. (1999) 174:279–84. doi: 10.1111/j.1574-6968.1999.tb13580.x 10339820

[323] Puértolas E de Marañón IM. Olive oil pilot-production assisted by pulsed electric field: impact on extraction yield, chemical parameters and sensory properties. Food Chem. (2015) 167:497–502. doi: 10.1016/j.foodchem.2014.07.029 25149017

[324] Gao X Wang Z Sun G Zhao Y Tang S Zhu H . Pulsed electric field (PEF) technology for preserving fruits and vegetables: applications, benefits, and comparisons. Food Rev Int. (2025) 1–26. doi: 10.1080/87559129.2025.2489754

[325] Donsì F Ferrari G Pataro G. Applications of pulsed electric field treatments for the enhancement of mass transfer from vegetable tissue. Food Eng Rev. (2010) 2:109–30. doi: 10.1007/s12393-010-9015-3

[326] Abelbaevich BT Zamzagul M Zhaksylykovna MB Zhanabayevna AL Sholpan T Yerkin Y . Enhancing food safety and quality through high-pressure processing and PEF technologies: comparative analysis. Casp J Environ Sci. (2024) 22:513–20. doi: 10.22124/CJES.2024.7742

[327] Bobinaitė R Pataro G Lamanauskas N Šatkauskas S Viškelis P Ferrari G. Application of pulsed electric field in the production of juice and extraction of bioactive compounds from blueberry fruits and their by-products. J Food Sci Technol. (2014) 52:5898–905. doi: 10.1007/s13197-014-1668-0 26345006 PMC4554608

[328] Brito IPC Silva EK. Pulsed electric field technology in vegetable and fruit juice processing: a review. Food Res Int. (2024) 184:114207. doi: 10.1016/j.foodres.2024.114207 38609209

[329] Grimi N Mamouni F Lebovka N Vorobiev E Vaxelaire J. Impact of apple processing modes on extracted juice quality: pressing assisted by pulsed electric fields. J Food Eng. (2011) 103:52–61. doi: 10.1016/j.jfoodeng.2010.09.019

[330] Chatzimitakos T Athanasiadis V Kalompatsios D Mantiniotou M Bozinou E Lalas SI. Pulsed electric field applications for the extraction of bioactive compounds from food waste and by-products: a critical review. Biomass. (2023) 3:367–401. doi: 10.3390/biomass3040022

[331] Puértolas E Cregenzán O Luengo E Álvarez I Raso J. Pulsed-electric-field-assisted extraction of anthocyanins from purple-fleshed potato. Food Chem. (2013) 136:1330–6. doi: 10.1016/j.foodchem.2012.09.080 23194531

[332] Bocker R Silva EK. Pulsed electric field assisted extraction of natural food pigments and colorings from plant matrices. Food Chem X. (2022) 15:100398. doi: 10.1016/j.fochx.2022.100398 36211728 PMC9532718

[333] Perez-Vazquez A Carpena M Barciela P Cassani L Simal-Gandara J Prieto MA. Pressurized liquid extraction for the recovery of bioactive compounds from seaweeds for food industry application: a review. Antioxidants. (2023) 12:612. doi: 10.3390/antiox12030612 36978860 PMC10045370

[334] Andreu V Picó Y. Pressurized liquid extraction of organic contaminants in environmental and food samples. TrAC Trends Anal Chem. (2019) 118:709–21. doi: 10.1016/j.trac.2019.06.038

[335] Camel V. Recent extraction techniques for solid matrices—supercritical fluid extraction, pressurized fluid extraction and microwave-assisted extraction: their potential and pitfalls. Analyst. (2001) 126:1182–93. doi: 10.1039/b008243k 11478658

[336] Richter BE Jones BA Ezzell JL Porter NL Avdalovic N Pohl C. Accelerated solvent extraction: a technique for sample preparation. Anal Chem. (1996) 68:1033–9. doi: 10.1021/ac9508199

[337] Ganjeh AM Saraiva JA Pinto CA Casal S Silva AM. Emergent technologies to improve protein extraction from fish and seafood by-products: an overview. Appl Food Res. (2023) 3:100339. doi: 10.1016/j.afres.2023.100339

[338] Dmitrienko S Apyari V Tolmacheva V Gorbunova M Furletov A Tsizin G . Methods for extraction of organic compounds from solid samples: 2. Sub-and supercritical extraction Matrix solid-phase dispersion QuEChERS method Review of reviews. J Anal Chem. (2024) 79:1167–87. doi: 10.1134/S1061934824700540

[339] Mustafa A Turner C. Pressurized liquid extraction as a green approach in food and herbal plants extraction: a review. Anal Chim Acta. (2011) 703:8–18. doi: 10.1016/j.aca.2011.07.018 21843670

[340] Machado APDF Pasquel-Reátegui JL Barbero GF Martínez J. Pressurized liquid extraction of bioactive compounds from blackberry (*Rubus fruticosus* L.) residues: a comparison with conventional methods. Food Res Int. (2015) 77:675–83. doi: 10.1016/j.foodres.2014.12.042

[341] Carabias-Martínez R Rodríguez-Gonzalo E Revilla-Ruiz P Hernández-Méndez J. Pressurized liquid extraction in the analysis of food and biological samples. J Chromatogr A. (2005) 1089:1–17. doi: 10.1016/j.chroma.2005.06.072 16130765

[342] Prasad W Wani AD Khamrui K Hussain SA Khetra Y. Green solvents, potential alternatives for petroleum based products in food processing industries. Cleaner Chem Eng. (2022) 3:100052. doi: 10.1016/j.clce.2022.100052

[343] Monrad JK Howard LR King JW Srinivas K Mauromoustakos A. Subcritical solvent extraction of anthocyanins from dried red grape pomace. J Agric Food Chem. (2010) 58:2862–8. doi: 10.1021/jf904087n 20148515

[344] Wijngaard H Hossain MB Rai DK Brunton N. Techniques to extract bioactive compounds from food by-products of plant origin. Food Res Int. (2012) 46:505–13. doi: 10.1016/j.foodres.2011.09.027

[345] Barp L Višnjevec AM Moret S. Pressurized liquid extraction: a powerful tool to implement extraction and purification of food contaminants. Foods. (2023) 12:2017. doi: 10.3390/foods12102017 37238835 PMC10217656

[346] Goulart AC Rodrigues AAZ Heleno FF de Faria AM Goulart SM de Queiroz MELR. Liquid-liquid and solid-liquid extractions with low-temperature partitioning–a review. Anal Chim Acta. (2024) 1316:342795. doi: 10.1016/j.aca.2024.342795 38969398

[347] Pronyk C Mazza G. Design and scale-up of pressurized fluid extractors for food and bioproducts. J Food Eng. (2009) 95:215–26. doi: 10.1016/j.jfoodeng.2009.06.002

[348] Huang G Zhang M Sun J Bai Y Li L Xue Z . Determination of flavonoids in *Magnolia officinalis* leaves based on response surface optimization of infrared assisted extraction followed by high-performance liquid chromatography (HPLC). Anal Lett. (2020) 53:2145–59. doi: 10.1080/00032719.2020.1732401

[349] Wang L Duan H Jiang J Long J Yu Y Chen G . simple and rapid infrared-assisted self enzymolysis extraction method for total flavonoid aglycones extraction from *Scutellariae radix* and mechanism exploration. Anal Bioanal Chem. (2017) 409:5593–602. doi: 10.1007/s00216-017-0497-1 28730309

[350] Cheaib D El Darra N Rajha HN El-Ghazzawi I Mouneimne Y Jammoul A . Study of the selectivity and bioactivity of polyphenols using infrared assisted extraction from apricot pomace compared to conventional methods. Antioxidants. (2018) 7:174. doi: 10.3390/antiox7120174 30486336 PMC6315536

[351] Chen Y Duan G Xie M Chen B Li Y. Infrared-assisted extraction coupled with high-performance liquid chromatography for simultaneous determination of eight active compounds in *Radix Salviae miltiorrhizae*. J Sep Sci. (2010) 33:2888–97. doi: 10.1002/jssc.201000234 20730830

[352] Abi-Khattar AM Rajha HN Abdel-Massih RM Maroun RG Louka N Debs E. Intensification of polyphenol extraction from olive leaves using Ired-Irrad^®^, an environmentally-friendly innovative technology. Antioxidants. (2019) 8:227. doi: 10.3390/antiox8070227 31323872 PMC6680986

[353] Cao S Liang J Chen M Xu C Wang X Qiu L . Comparative analysis of extraction technologies for plant extracts and absolutes. Front Chem. (2025) 13:1536590. doi: 10.3389/fchem.2025.1536590 40099208 PMC11911331

[354] Awad AM Kumar P Ismail-Fitry MR Jusoh S Ab Aziz MF Sazili AQ. Green extraction of bioactive compounds from plant biomass and their application in meat as natural antioxidant. Antioxidants. (2021) 10:1465. doi: 10.3390/antiox10091465 34573097 PMC8466011

[355] Deng BX Li B Li XD Zaaboul F Jiang J Li JW . Using short-wave infrared radiation to improve aqueous enzymatic extraction of peanut oil: evaluation of peanut cotyledon microstructure and oil quality. Eur J Lipid Sci Technol. (2018) 120:1700285. doi: 10.1002/ejlt.201700285

[356] Lenucci MS De Caroli M Marrese PP Iurlaro A Rescio L Böhm V . Enzyme-aided extraction of lycopene from high-pigment tomato cultivars by supercritical carbon dioxide. Food Chem. (2015) 170:193–202. doi: 10.1016/j.foodchem.2014.08.081 25306335

[357] Boulila A Hassen I Haouari L Mejri F Amor IB Casabianca H . Enzyme-assisted extraction of bioactive compounds from bay leaves (*Laurus nobilis* L.). Ind Crops Prod. (2015) 74:485–93. doi: 10.1016/j.indcrop.2015.05.050

[358] Sahne F Mohammadi M Najafpour GD Moghadamnia AA. Enzyme-assisted ionic liquid extraction of bioactive compound from turmeric (*Curcuma longa* L.): isolation, purification and analysis of curcumin. Ind Crops Prod. (2017) 95:686–94. doi: 10.1016/j.indcrop.2016.11.037

[359] Xu C Yagiz Y Borejsza-Wysocki W Lu J Gu L Ramírez-Rodrigues MM . Enzyme release of phenolics from muscadine grape (*Vitis rotundifolia* Michx.) skins and seeds. Food Chem. (2014) 157:20–9. doi: 10.1016/j.foodchem.2014.01.128 24679747

[360] Vasco-Correa J Zapata ADZ. Enzymatic extraction of pectin from passion fruit peel (*Passiflora edulis f. flavicarpa*) at laboratory and bench scale LWT. Food Sci Technol. (2017) 80:280–5. doi: 10.1016/j.lwt.2017.02.024

[361] Roda A De Faveri DM Giacosa S Dordoni R Lambri M. Effect of pre-treatments on the saccharification of pineapple waste as a potential source for vinegar production. J Clean Prod. (2016) 112:4477–84. doi: 10.1016/j.jclepro.2015.07.019

[362] Ameer K Shahbaz HM Kwon JH. Green extraction methods for polyphenols from plant matrices and their byproducts: a review. Compr Rev Food Sci Food Saf. (2017) 16:295–315. doi: 10.1111/1541-4337.12253 33371540

[363] Dzah CS Duan Y Zhang H Boateng NAS Ma H. Latest developments in polyphenol recovery and purification from plant by-products: a review. Trends Food Sci Technol. (2020) 99:375–88. doi: 10.1016/j.tifs.2020.03.003

[364] Ristivojević P Krstić Ristivojević M Stanković D Cvijetić I. Advances in extracting bioactive compounds from food and agricultural waste and by-products using natural deep eutectic solvents: a circular economy perspective. Molecules. (2024) 29:4717. doi: 10.3390/molecules29194717 39407645 PMC11478183

[365] Freitas DS Rocha D Castro TG Noro J Castro VI Teixeira MA . Green extraction of cork bioactive compounds using natural deep eutectic mixtures. ACS Sustain Chem Eng. (2022) 10:7974–89. doi: 10.1021/acssuschemeng.2c01422

[366] Huang MM Yiin CL Lock SSM Chin BLF Othman I Chan YH. Natural deep eutectic solvents (NADES) for sustainable extraction of bioactive compounds from medicinal plants: recent advances, challenges, and future directions. J Mol Liq. (2025) 425:127202. doi: 10.1016/j.molliq.2025.127202

[367] Ravimoorthy R Pottail L Sharma SC. Ionic liquids-based extraction of natural products from plants– An overview. J Mol Liq. (2025) 425:127226. doi: 10.1016/j.molliq.2025.127226

[368] Aggarwal N. Ionic liquid-based green solvents for extraction and purification of natural plant products. Curr Phys Chem. (2024) 14:184–93. doi: 10.2174/0118779468304352240423084047

[369] Verdía Barbár P Choudhary H Nakasu PS Al-Ghatta A Han Y Hopson C . Recent advances in the use of ionic liquids and deep eutectic solvents for lignocellulosic biorefineries and biobased chemical and material production. Chem Rev. (2025) 125:5461–583. doi: 10.1021/acs.chemrev.4c00754 40479538 PMC12203485

[370] Sun S Yu Y Jo Y Han JH Xue Y Cho M . Impact of extraction techniques on phytochemical composition and bioactivity of natural product mixtures. Front Pharmacol. (2025) 16:1615338. doi: 10.3389/fphar.2025.1615338 40808686 PMC12343529

[371] Bocker R Silva EK. Pulsed electric field technology as a promising pre-treatment for enhancing orange agro-industrial waste biorefinery. RSC Adv. (2024) 14:2116–33. doi: 10.1039/D3RA07848E 38196909 PMC10775899

[372] Radnia MR Mahdian E Sani AM Hesarinejad MA. Comparison of microwave and pulsed electric field methods on extracting antioxidant compounds from Arvaneh plant (*Hymenocrater platystegius* Rech. F). Sci Rep. (2024) 14:25903. doi: 10.1038/s41598-024-77380-z 39472490 PMC11522389

[373] Chongo Y. Extraction methods of bioactive compounds: a sustainability approach. J Food Sci Gastron. (2025) 3:29–37. doi: 10.5281/zenodo.14610634

[374] Napiórkowska-Baran K Treichel P Dardzińska A Majcherczak A Pilichowicz A Szota M . Immunomodulatory Effects of selected non-nutritive bioactive compounds and their role in optimal nutrition. Curr Issues Mol Biol. (2025) 47:89. doi: 10.3390/cimb47020089 39996810 PMC11854453

[375] Alshafei MM Mabrouk AM Hanafi EM Ramadan MM Korany RM Kassem SS . Prophylactic supplementation of microencapsulated *Boswellia serrata* and probiotic bacteria in metabolic syndrome rats. Food Biosci. (2023) 51:102325. doi: 10.1016/j.fbio.2022.102325

[376] Mohammed DM Yang X El-Messery TM Jiang X Zahran HA Gebremeskal YH . Bioactive *Moringa oleifera* and *Nigella sativa* oils microcapsules alleviate high-fat-diet induced hepatic oxidative damage and inflammation in rats. Food Biosci. (2025) 64:105873. doi: 10.1016/j.fbio.2025.105873

[377] El-Messery TM El-Said MM Salama HH Mohammed DM Ros G. Bioaccessibility of encapsulated mango peel phenolic extract and its application in milk beverage. Int J Dairy Sci. (2021) 16:29–40. doi: 10.3923/ijds.2021.29.40

[378] Capelezzo AP Mohr LC Dalcanton F de Mello JMM Fiori MA. β-Cyclodextrins as encapsulating agents of essential oils. In:Arora P Dhingra N, editors. Cyclodextrin-a Versatile Ingredient. London: IntechOpen (2018). p. 169–200. doi: 10.5772/intechopen.73568

[379] Drewnowski A Gomez-Carneros C. Bitter taste, phytonutrients, and the consumer: a review. Am J Clin Nutr. (2000) 72:1424–35. doi: 10.1093/ajcn/72.6.1424 11101467

[380] Dima S Dima C Iordăchescu G. Encapsulation of functional lipophilic food and drug biocomponents. Food Eng Rev. (2015) 7:417–38. doi: 10.1007/s12393-015-9115-1

[381] Li K Liu L McClements DJ Liu Z Liu X Liu F . review of the bioactive compounds of kiwifruit: Bioactivity, extraction, processing and challenges. Food Rev Int. (2024) 40:996–1027. doi: 10.1080/87559129.2023.2212033

[382] Soliman TN Mohammed DM El-Messery TM Elaaser M Zaky AA Eun JB . Microencapsulation of plant phenolic extracts using complex coacervation incorporated in ultrafiltered cheese against AlCl3-induced neuroinflammation in rats. Front Nutr. (2022) 9:929977. doi: 10.3389/fnut.2022.929977 35845781 PMC9278961

[383] Mehta N Kumar P Verma AK Umaraw P Kumar Y Malav OP . Microencapsulation as a noble technique for the application of bioactive compounds in the food industry: a comprehensive review. Appl Sci. (2022) 12:1424. doi: 10.3390/app12031424

[384] Bamidele OP Emmambux MN. Encapsulation of bioactive compounds by “extrusion” technologies: a review. Crit Rev Food Sci Nutr. (2021) 61:3100–18. doi: 10.1080/10408398.2020.1793724 32729723

[385] Sabry BA Badr AN Ahmed KA Desoukey MA Mohammed DM. Utilizing lemon peel extract and its nano-emulsion to control aflatoxin toxicity in rats. Food Biosci. (2022) 50:101998. doi: 10.1016/j.fbio.2022.101998

[386] Soliman TN Karam-Allah AAK Abo-Zaid EM Mohammed DM. Efficacy of nanoencapsulated *Moringa oleifera* L. seeds and *Ocimum tenuiflorum* L. leaves extracts incorporated in functional soft cheese on streptozotocin-induced diabetic rats. Phytomed Plus. (2024) 4:100598. doi: 10.1016/j.phyplu.2024.100598

[387] Anal AK Shrestha S Sadiq MB. Biopolymeric-based emulsions and their effects during processing, digestibility and bioaccessibility of bioactive compounds in food systems. Food Hydrocoll. (2019) 87:691–702. doi: 10.1016/j.foodhyd.2018.09.008

[388] Manzoor S Hussain SZ Amin T Bashir O Naseer B Jabeen A . The use of extrusion technology for encapsulation of bioactive components for their improved stability and bioavailability. Nutr Food Sci. (2023) 53:959–76. doi: 10.1108/NFS-04-2022-0125

[389] Zabot GL Schaefer Rodrigues F Polano Ody L Vinícius Tres M Herrera E Palacin H . Encapsulation of bioactive compounds for food and agricultural applications. Polymers. (2022) 14:4194. doi: 10.3390/polym14194194 36236142 PMC9571964

[390] Devi N Sarmah M Khatun B Maji TK. Encapsulation of active ingredients in polysaccharide–protein complex coacervates. Adv Colloid Interface Sci. (2017) 239:136–45. doi: 10.1016/j.cis.2016.05.009 27296302

[391] Alu'datt MH Alrosan M Gammoh S Tranchant CC Alhamad MN Rababah T . Encapsulation-based technologies for bioactive compounds and their application in the food industry: a roadmap for food-derived functional and health-promoting ingredients. Food Biosci. (2022) 50:101971. doi: 10.1016/j.fbio.2022.101971

[392] Shavronskaya DO Noskova AO Skvortsova NN Adadi P Nazarova EA. Encapsulation of hydrophobic bioactive substances for food applications: carriers, techniques, and biosafety. J Food Process Preserv. (2023) 2023:5578382. doi: 10.1155/2023/5578382

[393] Carrillo-Lopez LM Garcia-Galicia IA Tirado-Gallegos JM Sanchez-Vega R Huerta-Jimenez M Ashokkumar M . Recent advances in the application of ultrasound in dairy products: effect on functional, physical, chemical, microbiological and sensory properties. Ultrason Sonochem. (2021) 73:105467. doi: 10.1016/j.ultsonch.2021.105467 33508590 PMC7840480

[394] McClements DJ Gunasekaran S. Ultrasonic characterization of foods and drinks: principles, methods, and applications. Crit Rev Food Sci Nutr. (1997) 37:1–46. doi: 10.1080/10408399709527766 9067087

[395] Silva EK Zabot GL Hijo AAT Meireles MAA. Encapsulation of bioactive compounds using ultrasonic technology. In:Bermudez-Aguirre D, editor. Ultrasound: Advances for Food Processing and Preservation. Amsterdam: Elsevier (2017). p. 323–50. doi: 10.1016/B978-0-12-804581-7.00013-0

[396] Córdova A Henríquez P Nuñez H Rico-Rodriguez F Guerrero C Astudillo-Castro C . Recent advances in the application of enzyme processing assisted by ultrasound in agri-foods: a review. Catalysts. (2022) 12:107. doi: 10.3390/catal12010107

[397] Chemat F Khan MK. Applications of ultrasound in food technology: processing, preservation and extraction. Ultrason Sonochem. (2011) 18:813–35. doi: 10.1016/j.ultsonch.2010.11.023 21216174

[398] Goldberg BB Liu JB Forsberg F. Ultrasound contrast agents: a review. Ultrasound Med Biol. (1994) 20:319–33. doi: 10.1016/0301-5629(94)90001-9 8085289

[399] Tang Q Cao S Ma T Xiang X Luo H Borovskikh P . Engineering biofunctional enzyme-mimics for catalytic therapeutics and diagnostics. Adv Funct Mater. (2021) 31:2007475. doi: 10.1002/adfm.202007475

[400] Suslick KS Price GJ. Applications of ultrasound to materials chemistry. Annu Rev Mater Sci. (1999) 29:295–326. doi: 10.1146/annurev.matsci.29.1.295

[401] Zhu X Das RS Bhavya ML Garcia-Vaquero M Tiwari BK. Acoustic cavitation for agri-food applications: mechanism of action, design of new systems, challenges and strategies for scale-up. Ultrason Sonochem. (2024) 105:106850. doi: 10.1016/j.ultsonch.2024.106850 38520893 PMC10979275

[402] Leighton T. The Acoustic Bubble. Amsterdam: Elsevier (1994). p. 613.

[403] Namakshenas P Mojra A. Efficient drug delivery to hypoxic tumors using thermosensitive liposomes with encapsulated anti-cancer drug under high intensity pulsed ultrasound. Int J Mech Sci. (2023) 237:107818. doi: 10.1016/j.ijmecsci.2022.107818

[404] Kupikowska-Stobba B Domagała J Kasprzak MM. Critical review of techniques for food emulsion characterization. Appl Sci. (2024) 14:1069. doi: 10.3390/app14031069

[405] Kentish S Wooster T Ashokkumar M Balachandran S Mawson R Simons L. The use of ultrasonics for nanoemulsion preparation. Innov Food Sci Emerg Technol. (2008) 9:170–5. doi: 10.1016/j.ifset.2007.07.005

[406] Buvaneshwaran M Radhakrishnan M Natarajan V. Influence of ultrasound-assisted extraction techniques on the valorization of agro-based industrial organic waste – a review. J Food Process Eng. (2023) 46:e14012. doi: 10.1111/jfpe.14012

[407] Tao Y Zhang Z Sun DW. Kinetic modeling of ultrasound-assisted extraction of phenolic compounds from grape marc: influence of acoustic energy density and temperature. Ultrason Sonochem. (2014) 21:1461–9. doi: 10.1016/j.ultsonch.2014.01.029 24613646

[408] Moawad S El-Kalyoubi M Khallaf M Mohammed DM Mahmoud KF Farouk A. Effect of spray-drying on the physical, sensory, and *in vivo* parameters of orange peel oil and limonene. Egypt J Chem. (2022) 65:353–68. doi: 10.21608/ejchem.2022.127785.5669

[409] Yang DL Liu RK Wei Y Sun Q Wang JX. Micro-sized nanoaggregates: spray-drying-assisted fabrication and applications. Particuology. (2024) 85:22–48. doi: 10.1016/j.partic.2023.03.013

[410] Gharsallaoui A Roudaut G Chambin O Voilley A Saurel R. Applications of spray-drying in microencapsulation of food ingredients: an overview. Food Res Int. (2007) 40:1107–21. doi: 10.1016/j.foodres.2007.07.004

[411] Akbarbaglu Z Peighambardoust SH Sarabandi K Jafari SM. Spray drying encapsulation of bioactive compounds within protein-based carriers: different options and applications. Food Chem. (2021) 359:129965. doi: 10.1016/j.foodchem.2021.129965 33975145

[412] Zhang WF Chen XG Li PW Liu CS He QZ. Preparation and characterization of carboxymethyl chitosan and β-cyclodextrin microspheres by spray drying. Dry Technol. (2007) 26:108–15. doi: 10.1080/07373930701781736

[413] Kandasamy S Naveen R. A review on the encapsulation of bioactive components using spray-drying and freeze-drying techniques. J Food Process Eng. (2022) 45:e14059. doi: 10.1111/jfpe.14059

[414] Caliskan G Dirim SN. The effects of the different drying conditions and the amounts of maltodextrin addition during spray-drying of sumac extract. Food Bioprod Process. (2013) 91:539–48. doi: 10.1016/j.fbp.2013.06.004

[415] Fathi F Ebrahimi SN Matos LC Oliveira MBPP Alves RC. Emerging drying techniques for food safety and quality: a review. Compr Rev Food Sci Food Saf. (2022) 21:1125–60. doi: 10.1111/1541-4337.12898 35080792

[416] Zuidam NJ Heinrich E. Encapsulation of aroma. In:Zuidam N Nedovic V, editors. Encapsulation Technologies for Active Food Ingredients and Food Processing. New York, NY: Springer (2010). p. 127–60. doi: 10.1007/978-1-4419-1008-0_5

[417] Eijkelboom NM van Boven AP Siemons I Wilms PF Boom RM Kohlus R . Particle structure development during spray drying from a single droplet to pilot-scale perspective. J Food Eng. (2023) 337:111222. doi: 10.1016/j.jfoodeng.2022.111222

[418] Dalmoro A Barba AA Lamberti G. d'Amore M. Intensifying the microencapsulation process: ultrasonic atomization as an innovative approach. Eur J Pharm Biopharm. (2012) 80:471–7. doi: 10.1016/j.ejpb.2012.01.006 22285525

[419] Singh A Van den Mooter G. Spray drying formulation of amorphous solid dispersions. Adv Drug Deliv Rev. (2016) 100:27–50. doi: 10.1016/j.addr.2015.12.010 26705850

[420] Drosou CG Krokida MK Biliaderis CG. Encapsulation of bioactive compounds through electrospinning/electrospraying and spray drying: a comparative assessment of food-related applications. Dry Technol. (2017) 35:139–62. doi: 10.1080/07373937.2016.1162797

[421] Sobulska M Wawrzyniak P Woo MW. Superheated steam spray drying as an energy-saving drying technique: a review. Energies. (2022) 15:8546. doi: 10.3390/en15228546

[422] Kemp IC. Fundamentals of energy analysis of dryers. In:Tsotsas E Mujumdar AS, editors. Modern Drying Technology. Weinheim: Wiley-VCH Verlag GmbH & Co. KGaA (2012). p. 1–45.

[423] Parikh DM. Handbook of Pharmaceutical Granulation Technology. Boca Raton, FL: Taylor & Francis (2005). p. 613. doi: 10.1201/9780849354953

[424] Samborska K Poozesh S Barańska A Sobulska M Jedlińska A Arpagaus C . Innovations in spray drying process for food and pharma industries. J Food Eng. (2022) 321:110960. doi: 10.1016/j.jfoodeng.2022.110960

[425] Ishwarya SP Anandharamakrishnan C Stapley AGF. Spray-freeze-drying: a novel process for the drying of foods and bioproducts. Trends Food Sci Technol. (2015) 41:161–81. doi: 10.1016/j.tifs.2014.10.008

[426] Banožić M Vladić J Banjari I Velić D Aladić K Jokić S. Spray drying as a method of choice for obtaining high-quality products from food wastes—A review. Food Rev Int. (2023) 39:1953–85. doi: 10.1080/87559129.2021.1938601

[427] Thakur C Kaushal M Vaidya D Verma AK Gupta A Sharma R. Unlocking the potential of spray drying for agro-products: exploring advanced techniques, carrier agents, applications, and limitations. Food Bioprod Process. (2025) 18:1181–220. doi: 10.1007/s11947-024-03544-4

[428] Bhandari BR Patel KC Chen XD. Spray drying of food materials—process and product characteristics. Dry Technol Food Process. (2008) 4:113–57.

[429] Askari Vaselabadi S Gharibzahedi SMT Greiner R Vale JM Ovenseri AC Rashidinejad A . Advancements in spray-drying for the microencapsulation of fat-soluble vitamins: stability, bioavailability, and applications. J Food Biochem. (2025) 2025:9974476. doi: 10.1155/jfbc/9974476

[430] Angardi V Ettehadi A Yücel Ö. Critical review of emulsion stability and characterization techniques in oil processing. J Energy Resour Technol. (2022) 144:040801. doi: 10.1115/1.4051571

[431] Langrish TAG Premarajah R. Antioxidant capacity of spray-dried plant extracts: experiments and simulations. Adv Powder Technol. (2013) 24:771–9. doi: 10.1016/j.apt.2013.03.020

[432] Mohit M Xu M Kurnia JC Mujumdar AS Sasmito AP. Spray freezing: an overview of applications and modeling. Dry Technol. (2025) 43:34–52. doi: 10.1080/07373937.2024.2361360

[433] Favaro-Trindade CS de Matos Junior FE Okuro PK Dias-Ferreira J Cano A Severino P . Encapsulation of active pharmaceutical ingredients in lipid micro/nanoparticles for oral administration by spray-cooling. Pharmaceutics. (2021) 13:1186. doi: 10.3390/pharmaceutics13081186 34452147 PMC8399666

[434] Gibbs BF Kermasha S Alli I Mulligan CN. Encapsulation in the food industry: a review. Int J Food Sci Nutr. (1999) 50:213–24. doi: 10.1080/096374899101256 10627837

[435] Laein SS Samborska K Karaca AC Mostashari P Akbarbaglu Z Sarabandi K . Strategies for further stabilization of lipid-based delivery systems with a focus on solidification by spray-drying. Trends Food Sci Technol. (2024) 146:104412. doi: 10.1016/j.tifs.2024.104412

[436] Zuidam NJ Shimoni E. Overview of microencapsulates for use in food products or processes and methods to make them. In:Zuidam N Nedovic V, editors. Encapsulation Technologies for Active Food Ingredients and Food Processing. New York, NY: Springer (2010). p. 3–29. doi: 10.1007/978-1-4419-1008-0_2

[437] Pardeshi S More M Patil P Pardeshi C Deshmukh P Mujumdar A . A meticulous overview on drying-based (spray-, freeze-, and spray-freeze) particle engineering approaches for pharmaceutical technologies. Dry Technol. (2021) 39:1447–91. doi: 10.1080/07373937.2021.1893330

[438] Elkalla E Khizar S Tarhini M Lebaz N Zine N Jaffrezic-Renault N . Core-shell micro/nanocapsules: from encapsulation to applications. J Microencapsul. (2023) 40:125–56. doi: 10.1080/02652048.2023.2178538 36749629

[439] Farinha S Sá JV Lino PR Galésio M Pires J Rodrigues MÂ . Spray freeze drying of biologics: a review and applications for inhalation delivery. Pharm Res. (2023) 40:1115–40. doi: 10.1007/s11095-022-03442-4 36456666

[440] Oxley J. Spray cooling and spray chilling for food ingredient and nutraceutical encapsulation. In:Garti N McClements DJ, editors. Encapsulation Technologies and Delivery Systems for Food Ingredients and Nutraceuticals. Amsterdam: Elsevier (2012). p. 110–30. doi: 10.1533/9780857095909.2.110

[441] Chhabra N Arora M Garg D Samota MK. Spray freeze drying—a synergistic drying technology and its applications in the food industry to preserve bioactive compounds. Food Control. (2024) 155:110099. doi: 10.1016/j.foodcont.2023.110099

[442] Risch SJ. Encapsulation: overview of uses and techniques. In:Risch SJ Reineccius G, editors. Encapsulation And Controlled Release of Food Ingredients. ACS Symposium Series. Washington, DC: ACS Publications (1995). p. 2–7. doi: 10.1021/bk-1995-0590.ch001

[443] Zhou K Yang Y Zheng B Yu Q Huang Y Zhang N . Enhancing pullulan soft capsules with a mixture of glycerol and sorbitol plasticizers: a multi-dimensional study. Polymers. (2023) 15:2247. doi: 10.3390/polym15102247 37242822 PMC10224083

[444] Dimick KP Benjamin M. Process for preparing a solid flavoring composition. US Patent 2,904,440 Google Patents. Sep 15 (1959).

[445] Sánchez-Osorno DM López-Jaramillo MC Caicedo Paz AV Villa AL Peresin MS Martínez-Galán JP. Recent advances in the microencapsulation of essential oils, lipids, and compound lipids through spray drying: a review. Pharmaceutics. (2023) 15:1490. doi: 10.3390/pharmaceutics15051490 37242731 PMC10224131

[446] Alvim ID Stein MA Koury IP Dantas FBH Cruz CLDCV. Comparison between the spray drying and spray chilling microparticles contain ascorbic acid in a baked product application. LWT Food Sci Technol. (2016) 65:689–94. doi: 10.1016/j.lwt.2015.08.049

[447] Günel Z Varhan E Koç M Topuz A Sahin-Nadeem H. Production of pungency-suppressed capsaicin microcapsules by spray chilling. Food Biosci. (2021) 40:100918. doi: 10.1016/j.fbio.2021.100918

[448] Hernández A González-Moya M Márquez A Acevedo L. Review microalgae drying: a comprehensive exploration from conventional air drying to microwave drying methods. Future Foods. (2024) 10:100420. doi: 10.1016/j.fufo.2024.100420

[449] de Matos-Jr FE Comunian TA Thomazini M Favaro-Trindade CS. Effect of feed preparation on the properties and stability of ascorbic acid microparticles produced by spray chilling. LWT Food Sci Technol. (2017) 75:251–60. doi: 10.1016/j.lwt.2016.09.006

[450] Athar Alli SM. Coating processes of pharmaceutical applicability: a glimpse. J Drug Deliv Therapeut. (2022) 12:126. doi: 10.22270/jddt.v12i2.5362

[451] Nascimento RF de França PRL Ferreira MA Kurozawa LE. Assessment of the protective potential of coated microparticles in a fluidized bed against the simulated digestion. Food Res Int. (2025) 208:116273. doi: 10.1016/j.foodres.2025.116273 40263813

[452] Hemati M Cherif R Saleh K Pont V. Fluidized bed coating and granulation: influence of process-related variables and physicochemical properties on the growth kinetics. Powder Technol. (2003) 130:18–34. doi: 10.1016/S0032-5910(02)00221-8

[453] Guignon B Duquenoy A Dumoulin ED. Fluid bed encapsulation of particles: principles and practice. Drying Technol. (2002) 20:419–47. doi: 10.1081/DRT-120002550

[454] Leya B Nivetha TU Freeda Blessie R Pragalyaashree MM. Edible coating deposition methods: dipping, spraying, fluidized bed, and panning. In:Sen M, editor. Food Coatings and Preservation Technologies. Beverly, MA: Scrivener Publishing LLC (2024). p. 485–514. doi: 10.1002/9781394237623.ch14

[455] Song Y Zhou T Bai R Zhang M Yang H. Review of CFD-DEM modeling of wet fluidized bed granulation and coating processes. Processes. (2023) 11:382. doi: 10.3390/pr11020382

[456] Desai KGH Park HJ. Encapsulation of vitamin C in tripolyphosphate cross-linked chitosan microspheres by spray drying. J Microencapsul. (2005) 22:179–92. doi: 10.1080/02652040400026533 16019903

[457] Lashaki MJ Sarbanha AA Movahedirad S. Overall particles flow pattern in a two-zone gas-solid fluidized bed with a secondary-gas stream. Chem Eng Res Des. (2022) 187:570–83. doi: 10.1016/j.cherd.2022.09.023

[458] Yang WC Keairns DL. Rate of particle separation in a gas fluidized bed. Ind Eng Chem Fundam. (1982) 21:228–35. doi: 10.1021/i100007a007

[459] Choudhury N Meghwal M Das K. Microencapsulation: an overview on concepts, methods, properties and applications in foods. Food Front. (2021) 2:426–42. doi: 10.1002/fft2.94

[460] Soni RK Chinthapudi EK Tripathy SK Bose M. Goswami PS. Review on the chemical reduction modelling of hematite iron ore to magnetite in fluidized bed reactor. Rev Chem Eng. (2023) 39:1299–342. doi: 10.1515/revce-2022-0021

[461] Ghosh SK. Functional coatings and microencapsulation: a general perspective. In:Ghosh SK, editor. Functional Coatings: By Polymer Microencapsulation. Weinheim: Wiley-VCH Verlag GmbH & Co KGaA (2006). p. 1–28. doi: 10.1002/3527608478.ch1

[462] Liu M Xiao R Li X Zhao Y Huang J. A comprehensive review of recombinant technology in the food industry: exploring expression systems, application, and future challenges. Compr Rev Food Sci Food Saf. (2025) 24:e70078. doi: 10.1111/1541-4337.70078 39970011

[463] Arshady R. Microcapsules for food. J Microencapsul. (1993) 10:413–35. doi: 10.3109/02652049309015320 8263672

[464] Ranjan A Adhikari P Verma RK Parthiban A Singh M Kumar A. Advances in pharmaceutical coatings and coating materials. In:Arya RK Verros DG Davim JP, editors. Functional Coatings for Biomedical, Energy, and Environmental Applications. Hoboken, NJ: John Wiley & Sons, Inc. (2024). p. 145–62. doi: 10.1002/9781394263172.ch7

[465] Kydonieus AF. Controlled Release Technologies: Methods, Theory, and Applications. Boca Raton, FL: CRC Press (1980). p. 280.

[466] Song Y Yuan Y Zhu J. A review on applications of fine particles integrated with fluidization technologies. Can J Chem Eng. (2025) 103:1474–93. doi: 10.1002/cjce.25260

[467] Kocira A Kozłowicz K Panasiewicz K Staniak M Szpunar-Krok E Hortyńska P. Polysaccharides as edible films and coatings: characteristics and influence on fruit and vegetable quality—A review. Agronomy. (2021) 11:813. doi: 10.3390/agronomy11050813

[468] Kester JJ Fennema O. Edible films and coatings: a review. Food Technol. (1986) 40:47–59.

[469] Lobel BT Baiocco D Al-Sharabi M Routh AF Zhang Z Cayre OJ. Current challenges in microcapsule designs and microencapsulation processes: a review. ACS Appl Mater Interfaces. (2024) 16:40326–55. doi: 10.1021/acsami.4c02462 39042830 PMC11311140

[470] Shilton N Niranjan K. Fluidization and its applications to food processing. Food Struct. (1993) 12:8.

[471] Gosavi AA Nandgude TD Mishra RK Puri DB. Exploring the potential of artificial intelligence as a facilitating tool for formulation development in fluidized bed processor: a comprehensive review. AAPS PharmSciTech. (2024) 25:111. doi: 10.1208/s12249-024-02816-8 38740666

[472] Zeng H Xu K Wang F Sun S Li D Zhang J. Preparation of adsorbent based on water treatment residuals and chitosan by homogeneous method with freeze-drying and its As (V) removal performance. Int J Biol Macromol. (2021) 184:313–24. doi: 10.1016/j.ijbiomac.2021.06.032 34118290

[473] Authelin JR Koumurian B Meagher K Walsh E Clavreul T Rellis B . A simple and cost-effective technique to monitor the sublimation flow during primary drying of freeze-drying using shelf inlet/outlet temperature difference or chamber to condenser pressure drop. J Pharm Sci. (2024) 113:1898–906. doi: 10.1016/j.xphs.2024.02.015 38369018

[474] Celli GB Ghanem A Brooks MSL. Bioactive encapsulated powders for functional foods—a review of methods and current limitations. Food Bioproc Technol. (2015) 8:1825–37. doi: 10.1007/s11947-015-1559-z

[475] Mohammed DM Salem MB Elzallat M Hammam OA Suliman AA. Moringa oleifera L. mediated zinc oxide nano-biofertilizer alleviates non-alcoholic steatohepatitis via modulating *de novo* lipogenesis pathway and miRNA-122 expression. Food Biosci. (2024) 60:104286. doi: 10.1016/j.fbio.2024.104286

[476] Mardani M Siahtiri S Besati M Baghani M Baniassadi M Nejad AM. Microencapsulation of natural products using spray drying; an overview. J Microencapsul. (2024) 41:649–78. doi: 10.1080/02652048.2024.2389136 39133055

[477] Mascarenhas WJ Akay HU Pikal MJ. A computational model for finite element analysis of the freeze-drying process. Comput Methods Appl Mech Eng. (1997) 148:105–24. doi: 10.1016/S0045-7825(96)00078-3

[478] Waghmare RB Kumar M Panesar PS. Freeze-drying: basic principles and processes. In:Waghmare RB Kumar M Panesar PS, editors. Freeze Drying of Food Products: Fundamentals, Processes and Applications. Hoboken, NJ: John Wiley & Sons Ltd (2024). p. 1–29. doi: 10.1002/9781119982098.ch1

[479] Ceballos AM Giraldo GI Orrego CE. Effect of freezing rate on quality parameters of freeze dried soursop fruit pulp. J Food Eng. (2012) 111:360–5. doi: 10.1016/j.jfoodeng.2012.02.010

[480] Muhoza B Uriho A. Freeze-dried essential oils encapsulated in biopolymeric matrices: design, formulation, and stability: a comprehensive review. Food Biophys. (2025) 20:1–16. doi: 10.1007/s11483-025-09974-7

[481] Young SL Sarda X Rosenberg M. Microencapsulating properties of whey proteins. 1 Microencapsulation of anhydrous milk fat. J Dairy Sci. (1993) 76:2868–77. doi: 10.3168/jds.S0022-0302(93)77625-0

[482] Liu Y Zhang Z Hu L. High efficient freeze-drying technology in food industry. Crit Rev Food Sci Nutr. (2022) 62:3370–88. doi: 10.1080/10408398.2020.1865261 33393368

[483] Arora S Dash SK Dhawan D Sahoo PK Jindal A Gugulothu D. Freeze-drying revolution: unleashing the potential of lyophilization in advancing drug delivery systems. Drug Deliv Transl Res. (2024) 14:1111–53. doi: 10.1007/s13346-023-01477-7 37985541

[484] Pardeshi SR Deshmukh NS Telange DR Nangare SN Sonar YY Lakade SH . Process development and quality attributes for the freeze-drying process in pharmaceuticals, biopharmaceuticals and nanomedicine delivery: a state-of-the-art review. Future J Pharm Sci. (2023) 9:99. doi: 10.1186/s43094-023-00551-8

[485] Parthasarathi S Anandharamakrishnan C. Enhancement of oral bioavailability of vitamin E by spray-freeze drying of whey protein microcapsules. Food Bioprocess Technol. (2016) 100:469–76. doi: 10.1016/j.fbp.2016.09.004

[486] Alberti A Zielinski AAF Zardo DM Demiate IM Nogueira A Mafra LI. Optimization of the extraction of phenolic compounds from apples using response surface methodology. Food Chem. (2014) 149:151–8. doi: 10.1016/j.foodchem.2013.10.086 24295689

[487] Putnik P Barba FJ Lorenzo JM Gabrić D Shpigelman A Cravotto G . An integrated approach to mandarin processing: food safety and nutritional quality, consumer preference, and nutrient bioaccessibility. Compr Rev Food Sci Food Saf. (2017) 16:1345–58. doi: 10.1111/1541-4337.12310 33371593

[488] Anticona M Lopez-Malo D Frigola A Esteve MJ Blesa J. Comprehensive analysis of polyphenols from hybrid mandarin peels by SPE and HPLC-UV. LWT Food Sci Technol. (2022) 165:113770. doi: 10.1016/j.lwt.2022.113770

[489] Jiménez-Aguilar DM López-Martínez JM Hernández-Brenes C Gutiérrez-Uribe JA Welti-Chanes J. Dietary fiber, phytochemical composition and antioxidant activity of Mexican commercial varieties of cactus pear. J Food Compos Anal. (2015) 41:66–73. doi: 10.1016/j.jfca.2015.01.017

[490] Chang SF Hsieh CL Yen GC. The protective effect of *Opuntia dillenii* Haw fruit against low-density lipoprotein peroxidation and its active compounds. Food Chem. (2008) 106:569–75. doi: 10.1016/j.foodchem.2007.06.017

[491] Galati EM Mondello MR Giuffrida D Dugo G Miceli N Pergolizzi S. Taviano MF. Chemical characterization and biological effects of Sicilian *Opuntia ficus indica* (L.) M. fruit juice: antioxidant and antiulcerogenic activity. J Agric Food Chem. (2003) 51:4903–8. doi: 10.1021/jf030123d 12903943

[492] Lorenzo JM Pateiro M Domínguez R Barba FJ Putnik P Kovačević DB . Berries extracts as natural antioxidants in meat products: a review. Food Res Int. (2018) 106:1095–104. doi: 10.1016/j.foodres.2017.12.005 29579903

[493] Lorenzo JM Munekata PE Putnik P Kovačević DB Muchenje V Barba FJ. Sources, chemistry, and biological potential of ellagitannins and ellagic acid derivatives. Stud Nat Prod Chem. (2019) 60:189–221. doi: 10.1016/B978-0-444-64181-6.00006-1

[494] Szajdek A Borowska EJ. Bioactive compounds and health-promoting properties of berry fruits: a review. Plant Foods Hum Nutr. (2008) 63:147–56. doi: 10.1007/s11130-008-0097-5 18931913

[495] Nishimura T Egusa AS Nagao A Odahara T Sugise T Mizoguchi N . Phytosterols in onion contribute to a sensation of lingering of aroma, a koku attribute. Food Chem. (2016) 192:724–8. doi: 10.1016/j.foodchem.2015.06.075 26304403

[496] Bisen SP Emerald M. Nutritional and therapeutic potential of garlic and onion (*Allium* sp.). Curr Nutr Food Sci. (2016) 12:190–9. doi: 10.2174/1573401312666160608121954

[497] Nile SH Nile AS Keum YS Sharma K. Utilization of quercetin and quercetin glycosides from onion (*Allium cepa* L.) solid waste as an antioxidant, urease and xanthine oxidase inhibitors. Food Chem. (2017) 235:119–26. doi: 10.1016/j.foodchem.2017.05.043 28554615

[498] Montesano D Rocchetti G Putnik P Lucini L. Bioactive profile of pumpkin: an overview on terpenoids and their health-promoting properties. Curr Opin Food Sci. (2018) 22:81–7. doi: 10.1016/j.cofs.2018.02.003

[499] Ribeiro-Santos R Carvalho-Costa D Cavaleiro C Costa HS Albuquerque TG Castilho MC . A novel insight on an ancient aromatic plant: the rosemary (*Rosmarinus officinalis* L.). Trends Food Sci Technol. (2015) 45:355–68. doi: 10.1016/j.tifs.2015.07.015

[500] Sueishi Y Sue M Masamoto H. Seasonal variations of oxygen radical scavenging ability in rosemary leaf extract. Food Chem. (2018) 245:270–4. doi: 10.1016/j.foodchem.2017.10.085 29287370

[501] Nieto G. Biological activities of three essential oils of the Lamiaceae family. Medicines. (2017) 4:63. doi: 10.3390/medicines4030063 28930277 PMC5622398

[502] Berdahl DR McKeague J. Rosemary and sage extracts as antioxidants for food preservation. In:Shahidi F, editor. Handbook of Antioxidants for Food Preservation. Cambridge: Woodhead Publishing (2015). p. 177–217. doi: 10.1016/B978-1-78242-089-7.00008-7

[503] Kolac UK Ustuner MC Tekin N Ustuner D Colak E Entok E. The anti-inflammatory and antioxidant effects of *Salvia officinalis* on lipopolysaccharide-induced inflammation in rats. J Med Food. (2017) 20:1193–200. doi: 10.1089/jmf.2017.0035 29131698

[504] Wu J Ge S Liu H Wang S Chen S Wang J . Properties and antimicrobial activity of silver carp (*Hypophthalmichthys molitrix*) skin gelatin-chitosan films incorporated with oregano essential oil for fish preservation. Food Packag Shelf Life. (2014) 2:7–16. doi: 10.1016/j.fpsl.2014.04.004

[505] Tohidi B Rahimmalek M Arzani A. Essential oil composition, total phenolic, flavonoid contents, and antioxidant activity of *Thymus* species collected from different regions of Iran. Food Chem. (2017) 220:153–61. doi: 10.1016/j.foodchem.2016.09.203 27855883

[506] Moghimi R Ghaderi L Rafati H Aliahmadi A McClements DJ. Superior antibacterial activity of nanoemulsion of *Thymus daenensis* essential oil against *E*. coli. Food Chem. (2016) 194:410–5. doi: 10.1016/j.foodchem.2015.07.139 26471573

[507] Forbes-Hernández TY Giampieri F Gasparrini M Mazzoni L Quiles JL Alvarez-Suarez JM . The effects of bioactive compounds from plant foods on mitochondrial function: a focus on apoptotic mechanisms. Food Chem Toxicol. (2014) 68:154–82. doi: 10.1016/j.fct.2014.03.017 24680691

[508] Sharma K Mahato N Cho MH Lee YR. Converting citrus wastes into value-added products: economic and environmentally friendly approaches. Nutrition. (2017) 34:29–46. doi: 10.1016/j.nut.2016.09.006 28063510

[509] Criado MN Barba FJ Frígola A Rodrigo D. Effect of *Stevia rebaudiana* on oxidative enzyme activity and its correlation with antioxidant capacity and bioactive compounds. Food Bioprocess Technol. (2014) 7:1518–25. doi: 10.1007/s11947-013-1208-3

[510] Koubaa M Roselló-Soto E Šic žlabur J Režek Jambrak A Brncic M Grimi N . Current and new insights in the sustainable and green recovery of nutritionally valuable compounds from *Stevia rebaudiana* Bertoni. J Agric Food Chem. (2015) 63:6835–46. doi: 10.1021/acs.jafc.5b01994 26172915

[511] Bulotta S Celano M Lepore SM Montalcini T Pujia A Russo D. Beneficial effects of the olive oil phenolic components oleuropein and hydroxytyrosol: focus on protection against cardiovascular and metabolic diseases. J Transl Med. (2014) 12:219. doi: 10.1186/s12967-014-0219-9 25086598 PMC4237885

[512] Toufektsian MC de Lorgeril M Nagy N Salen P Donati MB Giordano L . Chronic dietary intake of plant-derived anthocyanins protects the rat heart against ischemia-reperfusion injury. J Nutr. (2008) 138:747–52. doi: 10.1093/jn/138.4.747 18356330

[513] Afshari F Seraj H Sadat Hashemi Z Timajchi M Ensiyeh O Ladan G . The cytotoxic effects of eggplant peel extract on human gastric adenocarcinoma cells and normal cells. Mod Med Lab J. (2018) 1:77–83. doi: 10.30699/mmlj17.1.2.77

[514] Vanden Berghe W. Epigenetic impact of dietary polyphenols in cancer chemoprevention: lifelong remodeling of our epigenomes. Pharmacol Res. (2012) 65:565–76. doi: 10.1016/j.phrs.2012.03.007 22465217

[515] Sharma P McClees SF Afaq F. Pomegranate for prevention and treatment of cancer: an update. Molecules. (2017) 22:177. doi: 10.3390/molecules22010177 28125044 PMC5560105

[516] Rettig MB Heber D An J Seeram NP Rao JY Liu H . Pomegranate extract inhibits androgen-independent prostate cancer growth through a nuclear factor-κB-dependent mechanism. Mol Cancer Ther. (2008) 7:2662–71. doi: 10.1158/1535-7163.MCT-08-0136 18790748 PMC2858627

[517] Faria A Pestana D Teixeira D de Freitas V Mateus N Calhau C. Blueberry anthocyanins and pyruvic acid adducts: anticancer properties in breast cancer cell lines. Phytother Res. (2010) 24:1862–9. doi: 10.1002/ptr.3213 20564502

[518] Salminen A Lehtonen M Suuronen T Kaarniranta K Huuskonen J. Terpenoids: natural inhibitors of NF-κB signaling with anti-inflammatory and anticancer potential. Cell Mol Life Sci. (2008) 65:2979–99. doi: 10.1007/s00018-008-8103-5 18516495 PMC11131807

[519] Barik SK Russell WR Moar KM Cruickshank M Scobbie L Duncan G . The anthocyanins in black currants regulate postprandial hyperglycaemia primarily by inhibiting α-glucosidase while other phenolics modulate salivary α-amylase, glucose uptake and sugar transporters. J Nutr Biochem. (2020) 78:108325. doi: 10.1016/j.jnutbio.2019.108325 31952012

[520] Yang L Shu L Yao DD Jia XB Yu SM. Study on the glucose-lowering effect of puerarin in STZ-induced diabetic mice. Chin J Hosp Pharm. (2014) 34:1338–42.

[521] Anhê FF Roy D Pilon G Dudonné S Matamoros S Varin TV . polyphenol-rich cranberry extract protects from diet-induced obesity, insulin resistance and intestinal inflammation in association with increased *Akkermansia* sp. population in the gut microbiota of mice. Gut. (2015) 64:872–83. doi: 10.1136/gutjnl-2014-307142 25080446

[522] Gong L Cao W Chi H Wang J Zhang H Liu J . Whole cereal grains and potential health effects: Involvement of the gut microbiota. Food Res Int. (2018) 103:84–102. doi: 10.1016/j.foodres.2017.10.025 29389647

[523] Velderrain-Rodriguez GR Quero J Osada J Martin-Belloso O Rodríguez-Yoldi MJ. Phenolic-rich extracts from avocado fruit residues as functional food ingredients with antioxidant and antiproliferative properties. Biomolecules. (2021) 11:977. doi: 10.3390/biom11070977 34356601 PMC8301936

[524] Mirza B Croley CR Ahmad M Pumarol J Das N Sethi G. Bishayee A. Mango (*Mangifera indica* L.): a magnificent plant with cancer preventive and anticancer therapeutic potential. Crit Rev Food Sci Nutr. (2021) 61:2125–51. doi: 10.1080/10408398.2020.1771678 32506936

[525] Donga S Bhadu GR Chanda S. Antimicrobial, antioxidant and anticancer activities of gold nanoparticles green synthesized using *Mangifera indica* seed aqueous extract. Artif Cells Nanomed Biotechnol. (2020) 48:1315–25. doi: 10.1080/21691401.2020.1843470 33226851

[526] Agourram A Ghirardello D Rantsiou K Zeppa G Belviso S Romane A . Phenolic content, antioxidant potential, and antimicrobial activities of fruit and vegetable by-product extracts. Int J Food Prop. (2013) 16:1092–104. doi: 10.1080/10942912.2011.576446

[527] Sanz-Puig M Pina-Pérez MC Martínez-López A Rodrigo D. Escherichia coli O157:H7 and *Salmonella typhimurium* inactivation by the effect of mandarin, lemon, and orange by-products in reference medium and in oat-fruit juice mixed beverage. LWT Food Sci Technol. (2016) 66:7–14. doi: 10.1016/j.lwt.2015.10.012

[528] Coman MM Oancea AM Verdenelli MC Cecchini C Bahrim GE Orpianesi C . Polyphenol content and *in vitro* evaluation of antioxidant, antimicrobial and prebiotic properties of red fruit extracts. Eur Food Res Technol. (2018) 244:735–45. doi: 10.1007/s00217-017-2997-9

[529] Ramadan H Min B Tiwari AK Reddy G Adesiyun A Hinton A . Antibacterial activity of pomegranate, orange and lemon peel extracts against food-borne pathogens and spoilage bacteria *in vitro* and on poultry skin. Int J Poult Sci. (2015) 14:229–39. doi: 10.3923/ijps.2015.229.239

[530] Wu J Goodrich KM Eifert JD Jahncke ML O'Keefe SF Welbaum GE . Inhibiting foodborne pathogens *Vibrio parahaemolyticus* and *Listeria monocytogenes* using extracts from traditional medicine: Chinese gallnut, pomegranate peel, Baikal skullcap root and forsythia fruit. Open Agric. (2018) 3:163–70. doi: 10.1515/opag-2018-0017

[531] Hillier JK. Pacific seamount volcanism in space and time. Geophys J Int. (2007) 168:877–89. doi: 10.1111/j.1365-246X.2006.03250.x

[532] Mordi RC Fadiaro AE Owoeye TF Olanrewaju IO Uzoamaka GC Olorunshola SJ. Identification by GC-MS of the components of oils of banana peels extract, phytochemical and antimicrobial analyses. Res J Phytochem. (2016) 10:39–44. doi: 10.3923/rjphyto.2016.39.44

[533] Xu C Yagiz Y Hsu WY Simonne A Lu J. Marshall MR. Antioxidant, antibacterial, and antibiofilm properties of polyphenols from muscadine grape (*Vitis rotundifolia* Michx.) pomace against selected foodborne pathogens. J Agric Food Chem. (2014) 62:6640–9. doi: 10.1021/jf501073q 24865879

[534] Casquete R Castro SM Martin A Ruiz-Moyano S Saraiva JA Cordoba MG . Evaluation of the effect of high pressure on total phenolic content, antioxidant and antimicrobial activity of citrus peels. Innov Food Sci Emerg Technol. (2015) 31:37–44. doi: 10.1016/j.ifset.2015.07.005

[535] de Almeida Rochelle SL Sardi JDCO Freires IA de Carvalho Galvão LC Lazarini JG de Alencar SM . The anti-biofilm potential of commonly discarded agro-industrial residues against opportunistic pathogens. Ind Crops Prod. (2016) 87:150–60. doi: 10.1016/j.indcrop.2016.03.044

[536] Gaafar AA Asker MS Salama ZA Bagato O Ali MA. In vitro, antiviral, antimicrobial and antioxidant potential activity of tomato pomace. Int J Pharm Sci Rev Res. (2015) 32:262–72.

[537] Liu A Hou A Chai L. Assessing human and environmental health in global diets from a perspective of economic growth. Sustain Prod Consum. (2024) 45:306–15. doi: 10.1016/j.spc.2024.01.011

[538] Vignesh A Amal TC Sarvalingam A Vasanth K. A review on the influence of nutraceuticals and functional foods on health. Food Chem Adv. (2024) 5:100749. doi: 10.1016/j.focha.2024.100749

[539] Kaur S Das M. Functional foods: an overview. Food Sci Biotechnol. (2011) 20:861. doi: 10.1007/s10068-011-0121-7

[540] Dutt S Manjul AS Chauhan M Changan SS Raigond P Singh B . Biotechnology for nutritional and associated processing quality improvement in potato. In:Jaiwal P Chhillar A Chaudhary D Jaiwal R, editors. Nutritional quality improvement in plants. Concepts and Strategies in Plant Sciences. Cham: Springer (2019). p. 429–43. doi: 10.1007/978-3-319-95354-0_15

[541] Gupta E Mishra P. Functional food with some health benefits, so-called superfood: a review. Curr Nutr Food Sci. (2021) 17:144–66. doi: 10.2174/1573401316999200717171048

[542] Shahidi F. Functional foods: their role in health promotion and disease prevention. J Food Sci. (2004) 69:R146–9. doi: 10.1111/j.1365-2621.2004.tb10727.x

[543] Kumar A Mosa KA Ji L Kage U Dhokane D Karre S Madalageri D . Metabolomics-assisted biotechnological interventions for developing plant-based functional foods and nutraceuticals. Crit Rev Food Sci Nutr. (2018) 58:1791–807. doi: 10.1080/10408398.2017.1285752 28272908

[544] Gong X Ji M Xu J Zhang C Li M. Hypoglycemic effects of bioactive ingredients from medicine food homology and medicinal health food species used in China. Crit Rev Food Sci Nutr. (2020) 60:2303–26. doi: 10.1080/10408398.2019.1634517 31309854

[545] Ren L Zhang J Zhang T. Immunomodulatory activities of polysaccharides from Ganoderma on immune effector cells. Food Chem. (2021) 340:127933. doi: 10.1016/j.foodchem.2020.127933 32882476

[546] Kim JH Doo EH Jeong M Kim S Lee YY Yang J . Enhancing immunomodulatory function of red ginseng through fermentation using *Bifidobacterium animalis* Subsp. lactis LT 19-2. Nutrients. (2019) 11:1481. doi: 10.3390/nu11071481 31261829 PMC6682942

[547] Jiang L Zhang G Li Y Shi G Li M. Potential application of plant-based functional foods in the development of immune boosters. Front Pharmacol. (2021) 12:637782. doi: 10.3389/fphar.2021.637782 33959009 PMC8096308

[548] Huang Q Li LY Liu QQ Wang Z. Advances in immunoregulation effects of *Ganoderma lucidum* polysaccharide and/or *Polyporus umbellatus* polysaccharide. Shipin Kexue/Food Sci. (2020) 41:275–82.

[549] Sun B Yu S Zhao D Guo S Wang X Zhao K. Polysaccharides as vaccine adjuvants. Vaccine. (2018) 36:5226–34. doi: 10.1016/j.vaccine.2018.07.040 30057282

[550] He Y Hu Z Li A Zhu Z Yang N Ying Z . Recent advances in biotransformation of saponins. Molecules. (2019) 24:2365. doi: 10.3390/molecules24132365 31248032 PMC6650892

[551] Wijesekara T Luo J Xu B. Critical review on anti-inflammation effects of saponins and their molecular mechanisms. Phytother Res. (2024) 38:2007–22. doi: 10.1002/ptr.8164 38372176

[552] Rajput ZI Hu SH Xiao CW Arijo AG. Adjuvant effects of saponins on animal immune responses. J Zhejiang Univ Sci B. (2007) 8:153–61. doi: 10.1631/jzus.2007.B0153 17323426 PMC1810383

[553] Liu J Wang X Yong H Kan J Jin C. Recent advances in flavonoid-grafted polysaccharides: Synthesis, structural characterization, bioactivities and potential applications. Int J Biol Macromol. (2018) 116:1011–25. doi: 10.1016/j.ijbiomac.2018.05.149 29800657

[554] Jiang LL Gong X Ji MY Wang CC Wang JH Li MH. Bioactive compounds from plant-based functional foods: a promising choice for the prevention and management of hyperuricemia. Foods. (2020) 9:973. doi: 10.3390/foods9080973 32717824 PMC7466221

[555] Song DX Jiang JG. Hypolipidemic components from medicine food homology species used in China: pharmacological and health effects. Arch Med Res. (2017) 48:569–81. doi: 10.1016/j.arcmed.2018.01.004 29452699

[556] Vilas-Boas AA Pintado M Oliveira AL. Natural bioactive compounds from food waste: toxicity and safety concerns. Foods. (2021) 10:1564. doi: 10.3390/foods10071564 34359434 PMC8304211

[557] Veneziani G Novelli E Esposto S Taticchi A Servili M. Applications of recovered bioactive compounds in food products. In:Galanakis CM, editor. Olive Mill Waste. Amsterdam: Elsevier (2017). p. 231–53. doi: 10.1016/B978-0-12-805314-0.00011-X

[558] Fidelis M de Oliveira SM Santos JS Escher GB Rocha RS Cruz AG. do Carmo MAV, Azevedo L, Kaneshima T, Oh WY, Shahidi F. From byproduct to a functional ingredient: Camu-camu (*Myrciaria dubia*) seed extract as an antioxidant agent in a yogurt model. J Dairy Sci. (2020) 103:1131–40. doi: 10.3168/jds.2019-17173 31759605

[559] Özalp Özen B Eren M Pala A Özmen I Soyer A. Effect of plant extracts on lipid oxidation during frozen storage of minced fish muscle. Int J Food Sci Technol. (2011) 46:724–31. doi: 10.1111/j.1365-2621.2010.02541.x

[560] Peng X Ma J Cheng KW Jiang Y Chen F Wang M. The effects of grape seed extract fortification on the antioxidant activity and quality attributes of bread. Food Chem. (2010) 119:49–53. doi: 10.1016/j.foodchem.2009.05.083

[561] Deolindo CTP Monteiro PI Santos JS Cruz AG da Silva MC Granato D. Phenolic-rich Petit Suisse cheese manufactured with organic Bordeaux grape juice, skin, and seed extract: technological, sensory, and functional properties. LWT Food Sci Technol. (2019) 115:108493. doi: 10.1016/j.lwt.2019.108493

[562] Zamuz S López-Pedrouso M Barba FJ Lorenzo JM Domínguez H Franco D. Application of hull, bur and leaf chestnut extracts on the shelf-life of beef patties stored under MAP: evaluation of their impact on physicochemical properties, lipid oxidation, antioxidant, and antimicrobial potential. Food Res Int. (2018) 112:263–73. doi: 10.1016/j.foodres.2018.06.053 30131137

[563] Lorenzo JM González-Rodríguez RM Sánchez M Amado IR Franco D. Effects of natural (grape seed and chestnut extract) and synthetic antioxidants (butylated hydroxytoluene, BHT) on the physical, chemical, microbiological and sensory characteristics of dry cured sausage “chorizo”. Food Res Int. (2013) 54:611–20. doi: 10.1016/j.foodres.2013.07.064

[564] Turgut SS Soyer A Işikçi F. Effect of pomegranate peel extract on lipid and protein oxidation in beef meatballs during refrigerated storage. Meat Sci. (2016) 116:126–32. doi: 10.1016/j.meatsci.2016.02.011 26878610

[565] Choe JH Kim HY Kim CJ. Effect of persimmon peel (*Diospyros kaki* Thumb.) extracts on lipid and protein oxidation of raw ground pork during refrigerated storage. Korean J Food Sci Anim Resour. (2017) 37:254. doi: 10.5851/kosfa.2017.37.2.254 28515649 PMC5434212

[566] Ergezer H Serdaroğlu M. Antioxidant potential of artichoke (*Cynara scolymus* L.) byproducts extracts in raw beef patties during refrigerated storage. J Food Meas Charact. (2018) 12:982–91. doi: 10.1007/s11694-017-9713-0

[567] Andres AI Petron MJ Delgado-Adamez J Lopez M Timon M. Effect of tomato pomace extracts on the shelf-life of modified atmosphere-packaged lamb meat. J Food Process Preserv. (2017) 41:e13018. doi: 10.1111/jfpp.13018

[568] Basiri S Shekarforoush SS Aminlari M Akbari S. The effect of pomegranate peel extract (PPE) on the polyphenol oxidase (PPO) and quality of Pacific white shrimp (*Litopenaeus vannamei*) during refrigerated storage. LWT Food Sci Technol. (2015) 60:1025–33. doi: 10.1016/j.lwt.2014.10.043

[569] Ebied AS Morshdy AEM Abd-El-Salam E Hussein MA Elewa ES. Effect of pomegranate peel powder on the hygienic quality of beef sausage. J Microbiol Biotechnol Food Sci. (2017) 6:1300–4. doi: 10.15414/jmbfs.2017.6.6.1300-1304

[570] Nishad J Koley TK Varghese E Kaur C. Synergistic effects of nutmeg and citrus peel extracts in imparting oxidative stability in meat balls. Food Res Int. (2018) 106:1026–36. doi: 10.1016/j.foodres.2018.01.075 29579894

[571] Biswas AK Beura CK Yadav AS Pandey NK Mendiratta SK Kataria JM. Influence of novel bioactive compounds from selected fruit by-products and plant materials on the quality and storability of microwave-assisted cooked poultry meat wafer during ambient temperature storage. LWT Food Sci Technol. (2015) 62:727–33. doi: 10.1016/j.lwt.2014.09.024

[572] Abid Y Azabou S Jridi M Khemakhem I Bouaziz M Attia H. Storage stability of traditional Tunisian butter enriched with antioxidant extract from tomato processing by-products. Food Chem. (2017) 233:476–82. doi: 10.1016/j.foodchem.2017.04.125 28530601

[573] Bertolino M Belviso S Dal Bello B Ghirardello D Giordano M Rolle L . Influence of the addition of different hazelnut skins on the physicochemical, antioxidant, polyphenol and sensory properties of yogurt. LWT Food Sci Technol. (2015) 63:1145–54. doi: 10.1016/j.lwt.2015.03.113

[574] Sah BNP Vasiljevic T McKechnie S Donkor ON. Effect of refrigerated storage on probiotic viability and the production and stability of antimutagenic and antioxidant peptides in yogurt supplemented with pineapple peel. J Dairy Sci. (2015) 98:5905–16. doi: 10.3168/jds.2015-9450 26142843

[575] Ortiz L Dorta E Gloria Lobo M Antonio González-Mendoza L Díaz C González M. Use of banana peel extract to stabilize antioxidant capacity and sensory properties of orange juice during pasteurization and refrigerated storage. Food Bioprocess Technol. (2017) 10:1883–91. doi: 10.1007/s11947-017-1964-6

[576] Zaky AA Hussein AS Mostafa S Abd El-Aty AM. Impact of sunflower meal protein isolate supplementation on pasta quality. Separations. (2022) 9:429. doi: 10.3390/separations9120429

[577] Kampuse S Ozola L Straumite E Galoburda R. Quality parameters of wheat bread enriched with pumpkin (*Cucurbita moschata*) by-products. Acta Univ Cibin Ser E Food Technol. (2015) 19:3–14. doi: 10.1515/aucft-2015-0010

[578] Šporin M Avbelj M Kovač B Možina SS. Quality characteristics of wheat flour dough and bread containing grape pomace flour. Food Sci Technol Int. (2018) 24:251–63. doi: 10.1177/1082013217745398 29207886

[579] Zaky AA Asiamah E El-Faham S Ashour M Sharaf A. Utilization of grape pomace extract as a source of natural antioxidant in biscuits. Eur Acad Res. (2020) 108–26.

[580] Arun KB Persia F Aswathy PS Chandran J Sajeev MS Jayamurthy P . Plantain peel – a potential source of antioxidant dietary fiber for developing functional cookies. J Food Sci Technol. (2015) 52:6355–64. doi: 10.1007/s13197-015-1727-1 26396380 PMC4573141

[581] Rowayshed G Sharaf AM El-Faham SY Ashour M Zaky AA. Utilization of potato peels extract as source of phytochemicals in biscuits. J Basic Appl Res Int. (2015) 8:190–201.

[582] El-Faham S Mohsen M Sharaf A Zaky A. Utilization of mango peels as a source of polyphenolic antioxidants. Curr Sci Int. (2016) 5:529–42.

[583] Mildner-Szkudlarz S Bajerska J Górnaś P Seglina D Pilarska A Jesionowski T. Physical and bioactive properties of muffins enriched with raspberry and cranberry pomace powder: a promising application of fruit by-products rich in biocompounds. Plant Foods Hum Nutr. (2016) 71:165–73. doi: 10.1007/s11130-016-0539-4 27037934 PMC4891392

[584] Hidalgo A Brandolini A Canadanović-Brunet J Cetković G Šaponjac VT. Microencapsulates and extracts from red beetroot pomace modify antioxidant capacity, heat damage and colour of pseudocereals-enriched einkorn water biscuits. Food Chem. (2018) 268:40–8. doi: 10.1016/j.foodchem.2018.06.062 30064775

[585] Mir SA Bosco SJD Shah MA Santhalakshmy S Mir MM. Effect of apple pomace on quality characteristics of brown rice based cracker. J Saudi Soc Agric Sci. (2017) 16:25–32. doi: 10.1016/j.jssas.2015.01.001

[586] Tańska M Roszkowska B Czaplicki S Borowska EJ Bojarska J Dabrowska A. Effect of fruit pomace addition on shortbread cookies to improve their physical and nutritional values. Plant Foods Hum Nutr. (2016) 71:307–13. doi: 10.1007/s11130-016-0561-6 27319014 PMC4996867

[587] Essien SO Udugama I Young B Baroutian S. Recovery of bioactives from kānuka leaves using subcritical water extraction: techno-economic analysis, environmental impact assessment and technology readiness level. J Supercrit Fluids. (2021) 169:105119. doi: 10.1016/j.supflu.2020.105119

[588] Lopeda-Correa M Valdés-Duque BE Osorio-Tobón JF. Ultrasound-assisted extraction of phenolic compounds from *Adenaria floribunda* stem: economic assessment. Foods. (2022) 11:2904. doi: 10.3390/foods11182904 36141034 PMC9498893

[589] Kumar SJ Kumar GV Dash A Scholz P Banerjee R. Sustainable green solvents and techniques for lipid extraction from microalgae: a review. Algal Res. (2017) 21:138–47. doi: 10.1016/j.algal.2016.11.014

[590] Putnik P Lorenzo JM Barba FJ Roohinejad S ReŽek Jambrak A Granato D . Novel food processing and extraction technologies of high-added value compounds from plant materials. Foods. (2018) 7:106. doi: 10.3390/foods7070106 29976906 PMC6069231

[591] Zawistowski J. Regulation of functional foods in selected Asian countries in the Pacific Rim. In:Bagchi D, editor. Nutraceutical and Functional Food Regulations in The United States and Around the World. Amsterdam: Elsevier (2008). p. 365–401. doi: 10.1016/B978-012373901-8.00024-X

[592] Zaky AA Akram MU Rybak K Witrowa-Rajchert D Nowacka M. Bioactive compounds from plants and by-products: Novel extraction methods, applications, and limitations. AIMS Mol Sci. (2024) 11:150–88. doi: 10.3934/molsci.2024010

[593] Beya MM Netzel ME Sultanbawa Y Smyth H Hoffman LC. Plant-based phenolic molecules as natural preservatives in comminuted meats: a review. Antioxidants. (2021) 10:263. doi: 10.3390/antiox10020263 33572049 PMC7915777

[594] Intrasook J Tsusaka TW Anal AK. Trends and current food safety regulations and policies for functional foods and beverages containing botanicals. J Food Drug Anal. (2024) 32:112. doi: 10.38212/2224-6614.3499 38934687 PMC11210467

[595] Cámara M Fernández-Ruiz V Díaz LD Hurtado RMC Mata MDCS. Global concepts and regulations in functional foods. In:Chhikara N Panghal A Chaudhary G, editors. Functional Foods. Beverly, MA: Scrivener Publishing LLC (2022). p. 511–54. doi: 10.1002/9781119776345.ch15

[596] Brody T. Food and dietary supplement package labeling—guidance from FDA's warning letters and Title 21 of the code of federal regulations. Compr Rev Food Sci Food Saf. (2016) 15:92–129. doi: 10.1111/1541-4337.12172 33371576

[597] Sgroi F Sciortino C Baviera-Puig A Modica F. Analyzing consumer trends in functional foods: a cluster analysis approach. J Agric Food Res. (2024) 15:101041. doi: 10.1016/j.jafr.2024.101041

[598] Dos Santos MS Wancura JH Oro CE Dallago RM Tres MV. Opportunities and challenges of plant bioactive compounds for food and agricultural-related areas. Phyton. (2022) 91:1105. doi: 10.32604/phyton.2022.020913

[599] Rashidinejad A. The road ahead for functional foods: promising opportunities amidst industry challenges. Future Postharvest Food. (2024) 1:266–73. doi: 10.1002/fpf2.12022

[600] Gómez Gómez CV Castillo Cortéz IG Martínez Montenegro I Ibañez San Martín L. The regulatory status of functional foods in the economic integration organizations of Latin America and the Caribbean. Arch Lat Am Nutr. (2023) 73:297–312. doi: 10.37527/2023.73.4.005

[601] Pai S Hebbar A Selvaraj S. A critical look at challenges and future scopes of bioactive compounds and their incorporations in the food, energy, and pharmaceutical sector. Environ Sci Pollut Res. (2022) 29:35518–41. doi: 10.1007/s11356-022-19423-4 35233673 PMC9079019

[602] Rakha A Shehzad A Khan K. Plant bioactives: challenges of extraction and processing. Front Nutr. (2024) 11:1357925. doi: 10.3389/fnut.2024.1357925 38292247 PMC10826700

[603] Safdar MN Kausar T Jabbar S Mumtaz A Ahad K Saddozai AA. Extraction and quantification of polyphenols from kinnow (*Citrus reticulate* L.) peel using ultrasound and maceration techniques. J Food Drug Anal. (2017) 25:488–500. doi: 10.1016/j.jfda.2016.07.010 28911634 PMC9328816

[604] Ferarsa S Zhang W Moulai-Mostefa N Ding L Jaffrin MY Grimi N. Recovery of anthocyanins and other phenolic compounds from purple eggplant peels and pulps using ultrasonic-assisted extraction. Food Bioprocess Technol. (2018) 109:19–28. doi: 10.1016/j.fbp.2018.02.006

[605] Skenderidis P Mitsagga C Giavasis I Petrotos K Lampakis D Leontopoulos S . The *in vitro* antimicrobial activity assessment of ultrasound assisted *Lycium barbarum* fruit extracts and pomegranate fruit peels. J Food Meas Charact. (2019) 13:2017–31. doi: 10.1007/s11694-019-00123-6

[606] Londoño-Londoño J de Lima VR Lara O Gil A Pasa TBC Arango GJ . Clean recovery of antioxidant flavonoids from citrus peel: Optimizing an aqueous ultrasound-assisted extraction method. Food Chem. (2010) 119:81–7. doi: 10.1016/j.foodchem.2009.05.075

[607] Zhu J Kou X Wu C Fan G Li T Dou J . Enhanced extraction of bioactive natural products using ultrasound-assisted aqueous two-phase system: application to flavonoids extraction from jujube peels. Food Chem. (2022) 395:133530. doi: 10.1016/j.foodchem.2022.133530 35777209

[608] Raj GB Dash KK. Ultrasound-assisted extraction of phytocompounds from dragon fruit peel: optimization, kinetics and thermodynamic studies. Ultrason Sonochem. (2020) 68:105180. doi: 10.1016/j.ultsonch.2020.105180 32502959

[609] Medina-Meza IG Barbosa-Cánovas GV. Assisted extraction of bioactive compounds from plum and grape peels by ultrasonics and pulsed electric fields. J Food Eng. (2015) 166:268–75. doi: 10.1016/j.jfoodeng.2015.06.012

[610] Sulejmanović M Milić N Mourtzinos I Nastić N Kyriakoudi A Drljača J . Ultrasound-assisted and subcritical water extraction techniques for maximal recovery of phenolic compounds from raw ginger herbal dust toward *in vitro* biological activity investigation. Food Chem. (2024) 437:137774. doi: 10.1016/j.foodchem.2023.137774 37866343

[611] Kumcuoglu S Yilmaz T Tavman S. Ultrasound assisted extraction of lycopene from tomato processing wastes. J Food Sci Technol. (2014) 51:4102–7. doi: 10.1007/s13197-013-0926-x 25477688 PMC4252437

[612] Saleh IA Vinatoru M Mason TJ Abdel-Azim NS Aboutabl EA Hammouda FM . A possible general mechanism for ultrasound-assisted extraction (UAE) suggested from the results of UAE of chlorogenic acid from *Cynara scolymus* L. (artichoke) leaves. Ultrason Sonochem. (2016) 31:330–6. doi: 10.1016/j.ultsonch.2016.01.002 26964956

[613] Altemimi A Lightfoot DA Kinsel M Watson DG. Employing response surface methodology for the optimization of ultrasound assisted extraction of lutein and β-carotene from spinach. Molecules. (2015) 20:6611–25. doi: 10.3390/molecules20046611 25875040 PMC6272631

[614] El Kantar S Rajha HN Maroun RG Louka N. Intensification of polyphenols extraction from orange peels using infrared as a novel and energy saving pretreatment. J Food Sci. (2020) 85:414–20. doi: 10.1111/1750-3841.15016 31968404

[615] Ivanović M Alañón ME Arráez-Román D Segura-Carretero AJFRI. Enhanced and green extraction of bioactive compounds from *Lippia citriodora* by tailor-made natural deep eutectic solvents. Food Res Int. (2018) 111:67–76. doi: 10.1016/j.foodres.2018.05.014 30007731

[616] Simsek M Sumnu G Sahin S. Microwave assisted extraction of phenolic compounds from sour cherry pomace. Sep Sci Technol. (2012) 47:1248–54. doi: 10.1080/01496395.2011.644616

[617] Asghari J Ondruschka B Mazaheritehrani M. Extraction of bioactive chemical compounds from the medicinal Asian plants by microwave irradiation. J Med Plant Res. (2011) 5:495–506.

[618] Brahim M Gambier F Brosse N. Optimization of polyphenols extraction from grape residues in water medium. Ind Crops Prod. (2014) 52:18–22. doi: 10.1016/j.indcrop.2013.10.030

[619] Ciriminna R Fidalgo A Avellone G Danzì C Timpanaro G Locatelli M . Integral extraction of *Opuntia ficus-indica* peel bioproducts via microwave-assisted hydrodiffusion and hydrodistillation. ACS Sustain Chem Eng. (2019) 7:7884–91. doi: 10.1021/acssuschemeng.9b00502

[620] Liazid A Guerrero RF Cantos E Palma M Barroso CG. Microwave assisted extraction of anthocyanins from grape skins. Food Chem. (2011) 124:1238–43. doi: 10.1016/j.foodchem.2010.07.053

[621] Torres-León C Rojas R Serna-Cock L Belmares-Cerda R Aguilar CN. Extraction of antioxidants from mango seed kernel: optimization assisted by microwave. Food Bioprocess Technol. (2017) 105:188–96. doi: 10.1016/j.fbp.2017.07.005

[622] Araújo RG Rodriguez-Jasso RM Ruiz HA Govea-Salas M Pintado ME Aguilar CN. Process optimization of microwave-assisted extraction of bioactive molecules from avocado seeds. Ind Crops Prod. (2020) 154:112623. doi: 10.1016/j.indcrop.2020.112623

[623] Yu X Bals O Grimi N Vorobiev E. A new way for the oil plant biomass valorization: polyphenols and proteins extraction from rapeseed stems and leaves assisted by pulsed electric fields. Ind Crops Prod. (2015) 74:309–18. doi: 10.1016/j.indcrop.2015.03.045

[624] Wang L Boussetta N Lebovka N Vorobiev E. Cell disintegration of apple peels induced by pulsed electric field and efficiency of bio-compound extraction. Food Bioprocess Technol. (2020) 122:13–21. doi: 10.1016/j.fbp.2020.03.004

[625] Parniakov O Roselló-Soto E Barba FJ Grimi N Lebovka N Vorobiev E. New approaches for the effective valorization of papaya seeds: extraction of proteins, phenolic compounds, carbohydrates, and isothiocyanates assisted by pulsed electric energy. Food Res Int. (2015) 77:711–7. doi: 10.1016/j.foodres.2015.03.031

[626] Koubaa M Barba FJ Grimi N Mhemdi H Koubaa W Boussetta N . Recovery of colorants from red prickly pear peels and pulps enhanced by pulsed electric field and ultrasound. Innov Food Sci Emerg Technol. (2016) 37:336–44. doi: 10.1016/j.ifset.2016.04.015

[627] Luengo E Álvarez I Raso J. Improving the pressing extraction of polyphenols of orange peel by pulsed electric fields. Innov Food Sci Emerg Technol. (2013) 17:79–84. doi: 10.1016/j.ifset.2012.10.005

[628] Corrales M Toepfl S Butz P Knorr D Tauscher B. Extraction of anthocyanins from grape by-products assisted by ultrasonics, high hydrostatic pressure or pulsed electric fields: a comparison. Innov Food Sci Emerg Technol. (2008) 9:85–91. doi: 10.1016/j.ifset.2007.06.002

[629] Gómez-Mejía E Rosales-Conrado N León-González ME Madrid Y. Citrus peels waste as a source of value-added compounds: extraction and quantification of bioactive polyphenols. Food Chem. (2019) 295:289–99. doi: 10.1016/j.foodchem.2019.05.136 31174761

[630] Benito-Román Ó Alvarez VH Alonso E Cocero MJ Saldaña MD. Pressurized aqueous ethanol extraction of β-glucans and phenolic compounds from waxy barley. Food Res Int. (2015) 75:252–9. doi: 10.1016/j.foodres.2015.06.006 28454954

[631] Markom M Hasan M Daud WRW Singh H. Jahim JM. Extraction of hydrolysable tannins from *Phyllanthus niruri* Linn.: effects of solvents and extraction methods. Sep Purif Technol. (2007) 52:487–96. doi: 10.1016/j.seppur.2006.06.003

[632] Mohd Jusoh NH Subki A Yeap SK Yap KC Jaganath IB. Pressurized hot water extraction of hydrosable tannins from *Phyllanthus tenellus* Roxb. BMC Chem. (2019) 13:134. doi: 10.1186/s13065-019-0653-0 31891160 PMC6925506

[633] Montañés F Catchpole OJ Tallon S Mitchell KA Scott D Webby RF. Extraction of apple seed oil by supercritical carbon dioxide at pressures up to 1300 bar. J Supercrit Fluids. (2018) 141:128–36. doi: 10.1016/j.supflu.2018.02.002

[634] Pavlić B Bera O Teslić N Vidović S Parpinello G Zeković Z. Chemical profile and antioxidant activity of sage herbal dust extracts obtained by supercritical fluid extraction. Ind Crops Prod. (2018) 120:305–12. doi: 10.1016/j.indcrop.2018.04.044

[635] Kitrytė V Laurinavičienė A Syrpas M Pukalskas A. Venskutonis PR. Modeling and optimization of supercritical carbon dioxide extraction for isolation of valuable lipophilic constituents from elderberry (*Sambucus nigra* L.) pomace. J CO_2_ *Util*. (2020) 35:225–35. doi: 10.1016/j.jcou.2019.09.020

[636] He L Zhang X Xu H Xu C Yuan F Knez Ž . Gao Y. Subcritical water extraction of phenolic compounds from pomegranate (*Punica granatum* L.) seed residues and investigation into their antioxidant activities with HPLC–ABTS+ assay. Food Bioprocess Process. (2012) 90:215–23. doi: 10.1016/j.fbp.2011.03.003

[637] Singh PP Saldaña MD. Subcritical water extraction of phenolic compounds from potato peel. Food Res Int. (2011) 44:2452–8. doi: 10.1016/j.foodres.2011.02.006

[638] Aliakbarian B Fathi A Perego P Dehghani F. Extraction of antioxidants from winery wastes using subcritical water. J Supercrit Fluids. (2012) 65:18–24. doi: 10.1016/j.supflu.2012.02.022

[639] Jiao G Kermanshahipour A. Extraction of anthocyanins from haskap berry pulp using supercritical carbon dioxide: influence of co-solvent composition and pretreatment. LWT Food Sci Technol. (2018) 98:237–44. doi: 10.1016/j.lwt.2018.08.042

[640] Natolino A Da Porto C. Supercritical carbon dioxide extraction of pomegranate (*Punica granatum* L.) seed oil: kinetic modelling and solubility evaluation. J Supercrit Fluids. (2019) 151:30–9. doi: 10.1016/j.supflu.2019.05.002

[641] e Santos DN de Souza LL Ferreira NJ de Oliveira AL. Study of supercritical extraction from Brazilian cherry seeds (*Eugenia uniflora* L.) with bioactive compounds. Food Bioprocess Process. (2015) 94:365–74. doi: 10.1016/j.fbp.2014.04.005

[642] Grzelak-Blaszczyk K Karlinska E Grzeda K Roj E Kolodziejczyk K. Defatted strawberry seeds as a source of phenolics, dietary fiber and minerals. LWT Food Sci Technol. (2017) 84:18–22. doi: 10.1016/j.lwt.2017.05.014

[643] Abdelmoez W Nage SM Bastawess A Ihab A Yoshida H. Subcritical water technology for wheat straw hydrolysis to produce value added products. J Cleaner Prod. (2014) 70:68–77. doi: 10.1016/j.jclepro.2014.02.011

[644] Rodrigues LGG Mazzutti S Vitali L Micke GA Ferreira SRS. Recovery of bioactive phenolic compounds from papaya seeds agro-industrial residue using subcritical water extraction. Biocat Agric Biotechnol. (2019) 22:101367. doi: 10.1016/j.bcab.2019.101367

[645] Pereira MG Hamerski F Andrade EF. Scheer AdP, Corazza ML. Assessment of subcritical propane, ultrasound-assisted and Soxhlet extraction of oil from sweet passion fruit (*Passiflora alata* Curtis) seeds. J Supercrit Fluids. (2017) 128:338–48. doi: 10.1016/j.supflu.2017.03.021

[646] Duba KS Casazza AA Mohamed HB Perego P Fiori L. Extraction of polyphenols from grape skins and defatted grape seeds using subcritical water: experiments and modeling. Food Bioprocess Technol. (2015) 94:29–38. doi: 10.1016/j.fbp.2015.01.001

[647] Fitri ZA Ahmadi F Islam MA Ponnampalam EN Dunshea FR Suleria HA . A systematic review of extraction methods, phytochemicals, and food applications of *Moringa oleifera* leaves using PRISMA methodology. Food Sci Nutr. (2025) 13:e70138. doi: 10.1002/fsn3.70138 40302917 PMC12037701

[648] Teixeira BA Vidigal MCTR Stringheta PC. Optimization of ultrasound-assisted extraction of anthocyanins from purple tomatoes. Ciencia Rural. (2023) 54:e20220604. doi: 10.1590/0103-8478cr20220604

[649] Xie Y Zhang L Wu W Xie J Gao B Xiao Y. Du Zhu. Sustainable and green extraction of citrus peel essential oil using intermittent solvent-free microwave technology. Bioresour Bioprocess. (2025) 12:48. doi: 10.1186/s40643-025-00885-6 40439974 PMC12122996

[650] Roobab U Aadil RM Kurup SS Maqsood S. Comparative evaluation of ultrasound-assisted extraction with other green extraction methods for sustainable recycling and processing of date palm bioresources and by-products: a review of recent research. Ultrason Sonochem. (2025) 114:107252. doi: 10.1016/j.ultsonch.2025.107252 39985822 PMC11904522

[651] Shahid S Pantakani M Binder L Fischer A Pantakani K Asif AR. Small molecule BRD4 inhibitors apabetalone and JQ1 rescues endothelial cells dysfunction, protects monolayer integrity and reduces midkine expression. Molecules. (2022) 27:7453. doi: 10.3390/molecules27217453 36364277 PMC9692972

[652] Kumar KA Gomez S. Microwave-assisted extraction of bioactives in fruits and vegetables: a comprehensive review. J Food Bioact. (2024) 28:41–9. doi: 10.26599/JFB.2024.95028394

[653] Cavalluzzi MM Lamonaca A Rotondo NP Miniero DV Muraglia M Gabriele P . Microwave-assisted extraction of bioactive compounds from lentil wastes: antioxidant activity evaluation and metabolomic characterization. Molecules. (2022) 27:7471. doi: 10.3390/molecules27217471 36364300 PMC9655545

[654] Vu VNH Cao TQ Nguyen TTH Nguyen LTN Le PH Nguyen V. Extraction of bioactive compounds from cocoa pod husk (*Theobroma cacao* L.) using deep eutectic solvent assisted with ultrasound. Nat Prod Commun. (2025) 20:1934578X251333026. doi: 10.1177/1934578X251333026

[655] Manach C Scalbert A Morand C Rémésy C Jiménez L. Polyphenols: food sources and bioavailability. Am J Clin Nutr. (2004) 79:727–47. doi: 10.1093/ajcn/79.5.727 15113710

[656] Bennett RN Shiga TM Hassimotto NM Rosa EA Lajolo FM. Cordenunsi BR. Phenolics and antioxidant properties of fruit pulp and cell wall fractions of postharvest banana (*Musa acuminata* Juss) cultivars. J Agric Food Chem. (2010) 58:7991–8003. doi: 10.1021/jf1008692 20553046

[657] Singh B Singh JP Kaur A Singh N. Bioactive compounds in banana and their associated health benefits–A review. Food Chem. (2016) 206:1–11. doi: 10.1016/j.foodchem.2016.03.033 27041291

[658] Erlund I. Review of the flavonoids quercetin, hesperetin, and naringenin. Dietary sources, bioactivities, bioavailability, and epidemiology. Nutr Res. (2004) 24:851–74. doi: 10.1016/j.nutres.2004.07.005

[659] Liggins J Bluck L Runswick S Atkinson C Coward W Bingham S. Daidzein and genistein contents of vegetables. Br J Nutr. (2000) 84:717–25. doi: 10.1017/S0007114500002075 11177186

[660] Cheng DM Pogrebnyak N Kuhn P Poulev A Waterman C Rojas-Silva P . Polyphenol-rich Rutgers Scarlet lettuce improves glucose metabolism and liver lipid accumulation in diet-induced obese C57BL/6 mice. Nutrition. (2014) 30:S52–8. doi: 10.1016/j.nut.2014.02.022 24985107 PMC4082798

[661] Ghofrani S Joghataei MT Mohseni S Baluchnejadmojarad T Bagheri M Khamse S . Naringenin improves learning and memory in an Alzheimer's disease rat model: insights into the underlying mechanisms. Eur J Pharmacol. (2015) 764:195–201. doi: 10.1016/j.ejphar.2015.07.001 26148826

[662] Nyman NA Kumpulainen JT. Determination of anthocyanidins in berries and red wine by high-performance liquid chromatography. J Agric Food Chem. (2001) 49:4183–7. doi: 10.1021/jf010572i 11559107

[663] Sorrenti V Burò I Consoli V Vanella L. Recent advances in health benefits of bioactive compounds from food wastes and by-products: biochemical aspects. Int J Mol Sci. (2023) 24:2019. doi: 10.3390/ijms24032019 36768340 PMC9916361

[664] Gumul D Ziobro R Korus J Kruczek M. Apple pomace as a source of bioactive polyphenol compounds in gluten-free breads. Antioxidants. (2021) 10:807. doi: 10.3390/antiox10050807 34069723 PMC8161145

[665] Martí R Roselló S Cebolla-Cornejo J. Tomato as a source of carotenoids and polyphenols targeted to cancer prevention. Cancers. (2016) 8:58. doi: 10.3390/cancers8060058 27331820 PMC4931623

[666] Yang D Jiang Y Wang Y Lei Q Zhao X Yi R . Improvement of flavonoids in lemon seeds on oxidative damage of human embryonic kidney 293T cells induced by H2O2. Oxid Med Cell Longev. (2020) 2020:3483519. doi: 10.1155/2020/3483519 32377296 PMC7189339

[667] Chhikara N Kushwaha K Sharma P Gat Y Panghal A. Bioactive compounds of beetroot and utilization in food processing industry: a critical review. Food Chem. (2019) 272:192–200. doi: 10.1016/j.foodchem.2018.08.022 30309532

[668] Wani TA Majid D Dar B Makroo HA Allai FM. Utilization of novel techniques in extraction of polyphenols from grape pomace and their therapeutic potential: a review. J Food Meas Charact. (2023) 17:5412–25. doi: 10.1007/s11694-023-02040-1

[669] Zhu J Lu Y He Q. Recent advances on bioactive compounds, health benefits, and potential applications of jujube (*Ziziphus jujuba* Mill): a perspective of by-products valorization. Trends Food Sci Technol. (2024) 145:104368. doi: 10.1016/j.tifs.2024.104368

[670] Pirzadeh M Caporaso N Rauf A Shariati MA Yessimbekov Z Khan MU . Pomegranate as a source of bioactive constituents: a review on their characterization, properties and applications. Crit Rev Food Sci Nutr. (2021) 61:982–99. doi: 10.1080/10408398.2020.1749825 32314615

[671] Amagase H Petesch BL Matsuura H Kasuga S Itakura Y. Intake of garlic and its bioactive components. J Nutr. (2001) 131:955S−62S. doi: 10.1093/jn/131.3.955S 11238796

[672] Fahey JW Zhang Y Talalay P. Broccoli sprouts: an exceptionally rich source of inducers of enzymes that protect against chemical carcinogens. Proc Natl Acad Sci USA. (1997) 94:10367–72. doi: 10.1073/pnas.94.19.10367 9294217 PMC23369

[673] Al-Madhagy S Ashmawy NS Mamdouh A Eldahshan OA Farag MA. A comprehensive review of the health benefits of flaxseed oil in relation to its chemical composition and comparison with other omega-3-rich oils. Eur J Med Res. (2023) 28:240. doi: 10.1186/s40001-023-01203-6 37464425 PMC10353157

[674] Ananingsih VK Sharma A Zhou W. Green tea catechins during food processing and storage: a review on stability and detection. Food Res Int. (2013) 50:469–79. doi: 10.1016/j.foodres.2011.03.004

[675] Samanta S Sarkar T Chakraborty R. Multifunctional applications of natural colorants: preservative, functional ingredient, and sports supplements. Biocatal Agric Biotechnol. (2024) 56:103026. doi: 10.1016/j.bcab.2024.103026

[676] Messina M. Soyfoods, soybean isoflavones, and bone health: a brief overview. J Ren Nutr. (2000) 10:63–8. doi: 10.1016/S1051-2276(00)90001-3 10757817

